# Genome-wide identification of Brassicaceae histone modification genes and their responses to abiotic stresses in allotetraploid rapeseed

**DOI:** 10.1186/s12870-023-04256-1

**Published:** 2023-05-11

**Authors:** Lin-Lin Hu, Li-Wei Zheng, Xin-Lei Zhu, Sheng-Jie Ma, Kai-Yan Zhang, Ying-Peng Hua, Jin-Yong Huang

**Affiliations:** 1 School of Agricultural Sciences, Zhengzhou University, Zhengzhou, 450001 China; 2 Zhengzhou Key Laboratory of Quality Improvement and Efficient Nutrient Use for Main Economic Crops, Henan, China; 3 School of Life Sciences, Zhengzhou University, Zhengzhou, 450001 China

**Keywords:** Brassicaceae, Allotetraploid rapeseed, Histone modification, Abiotic stress

## Abstract

**Background:**

Histone modification is an important epigenetic regulatory mechanism and essential for stress adaptation in plants. However, systematic analysis of histone modification genes (*HMs*) in Brassicaceae species is lacking, and their roles in response to abiotic stress have not yet been identified.

**Results:**

In this study, we identified 102 *AtHMs*, 280 *BnaHMs*, 251 *BcHMs*, 251 *BjHMs*, 144 *BnHMs*, 155 *BoHMs*, 137 *BrHMs*, 122 *CrHMs*, and 356 *CsHMs* in nine Brassicaceae species, respectively. Their chromosomal locations, protein/gene structures, phylogenetic trees, and syntenies were determined. Specific domains were identified in several Brassicaceae *HMs*, indicating an association with diverse functions. Syntenic analysis showed that the expansion of Brassicaceae *HMs* may be due to segmental and whole-genome duplications. Nine key *BnaHMs* in allotetraploid rapeseed may be responsible for ammonium, salt, boron, cadmium, nitrate, and potassium stress based on co-expression network analysis. According to weighted gene co-expression network analysis (WGCNA), 12 *BnaHMs* were associated with stress adaptation. Among the above genes, *BnaPRMT11* simultaneously responded to four different stresses based on differential expression analysis, while *BnaSDG46*, *BnaHDT10*, and *BnaHDA1* participated in five stresses. *BnaSDG46* was also involved in four different stresses based on WGCNA, while *BnaSDG10* and *BnaJMJ58* were differentially expressed in response to six different stresses. In summary, six candidate genes for stress resistance (*BnaPRMT11*, *BnaSDG46*, *BnaSDG10*, *BnaJMJ58*, *BnaHDT10*, and *BnaHDA1*) were identified.

**Conclusions:**

Taken together, these findings help clarify the biological roles of Brassicaceae *HMs*. The identified candidate genes provide an important reference for the potential development of stress-tolerant oilseed plants.

**Supplementary Information:**

The online version contains supplementary material available at 10.1186/s12870-023-04256-1.

## Background

Histone modification (HM) is an epigenetic regulatory mechanism that plays crucial roles in various aspects of plant growth and stress response by activating or silencing gene expression [1–4]. HM genes (*HMs*) include histone methyltransferases (*HMTs*), histone demethylases (*HDMs*), histone acetylases (*HATs*), and histone deacetylases (*HDACs*) [5–8].

*HMTs* are encoded by the SET-domain group (*SDG*) and protein arginine methyltransferase (*PRMT*) genes and catalyze HM [9]. Methylation and environmental factors are related to stress, which affects gene expression by changing methylation levels and stress resistance [10]. Several processes, such as fungal pathogen resistance, shoot and root branching, circadian cycle, hormone regulation, abscisic acid (ABA) morphogenesis, and salt stress, are affected by *HMTs* [11, 12]. For example, *AtSDG8* is involved in the regulation of shoot meristem activity, while *AtSDG26* and *AtPRMT10* are involved in the regulation of flowering in *Arabidopsis* [13–15]. Histone modification can be erased by *HDMs*, including lysine-specific demethylase 1 (LSD1) and Jumonji C (JmjC) domain containing proteins [16–18]. *HDMs* function in brassinosteroid (BR) signaling, pollen development, chromatin regulation, floral induction, and floral organ formation [19, 20]. In *Arabidopsis*, *JMJ30* expression changes in response to environmental stimuli, e.g., enhancement by salt and heat stress [21, 22], and flower repressor *JMJ13* can be affected by temperature and photoperiod [23]. *HATs* and *HDACs* catalyze the transfer of acetyl groups from acetyl-CoA to lysine residues [24, 25]. *HATs* and *HDACs* participate in the regulation of developmental transition, environmental signal responses, reproductive development, and gene silencing [26–28]. For example, *HAC1* inactivation affects both vegetative and reproductive development in *Arabidopsis* [29], *AtSRT2* regulates salt tolerance during seed germination [30], and *AtHDT4* participates in abiotic stress responses [31]. Previous studies have suggested that modifications affect functions, including transcriptional regulation of other genes in yeast [32].

Abiotic stress, such salinity, inappropriate nutrition, and metal toxicity, can adversely affect crop growth and yield [33, 34]. Nutrient imbalances, membrane damage, and dysfunctional antioxidant system can occur under soil salinization [35]. Various nutrients are essential for optimal plant growth and yield. Nitrogen (N) is a macronutrient “life element” that strongly affects plant growth and development [36], while excess ammonium (NH_4_^+^), an inorganic N nutrient, is toxic to plants [37]. Phosphorus (P) shares essential roles in regulating plant energy metabolism, and its deficiency can reduce cell division and elongation in grass leaves [38]. Potassium (K) is a vital macronutrient for plant growth and organ development, and participates in many physiological processes, such as osmoregulation. Moreover, K + transport participates in abiotic stress responses [39, 40]. Boron (B) is a micronutrient essential for the transport of carbohydrates, although both excess and deficiency can adversely impact crop growth and yield [41, 42]. Plants can also be affected by non-essential heavy metals, such as cadmium (Cd), which is highly biotoxic and easily absorbed by plants through sewage effluent, industrial waste, and agricultural run-off [43].

Brassicaceae plants are important and economically valuable crops, noted for their oil production [44, 45]. Given their immobility, plants are unable to avoid abiotic and biotic stresses, which can impair growth, development, and production. However, plants can adapt to stress by activating a series of physiological and molecular mechanisms, such as HM [46, 47]. Therefore, improving stress resistance and yield in Brassicaceae plants is a key goal of breeding [48]. To date, however, few studies have explored the regulation of gene expression related to stress resistance or conducted systematic study of *HMs* in Brassicaceae species. Here, we conducted a comprehensive study of *HMs* in nine Brassicaceae species, including *Arabidopsis thaliana*, *Brassica napus*, *Brassica carinata*, *Brassica juncea*, *Brassica nigra*, *Brassica oleracea*, *Brassica rapa*, *Capsella rubella*, and *Camelina sativa*. We further determined their chromosomal locations, conserved domains, gene structures, phylogenetic relationships, and syntenies. The responses of *HMs* to NH_4_^+^ toxicity, B deficiency and excess, Cd exposure, K shortage, N limitation, P starvation, and salt stress were explored in allotetraploid rapeseed. Potential candidate *BnaHMs* that responded to the above stresses were also identified. This study provides important clues for understanding the Brassicaceae *HM* gene family.

## Results

### Genome-wide identification of Brassicaceae ***HMs*** and their phylogenetic analysis

In the present study, we identified 1 798 *HMs*, including 102, 280, 251, 251, 144, 155, 137, 122, and 356 in *(A) thaliana*, *(B) napus*, *B. carinata*, *B. juncea*, *B. nigra*, *B. oleracea*, *B. rapa*, *(C) rubella*, and *C. sativa* (Figure S1 and Table S1). The number of *HMTs*, *HDMs*, *HATs*, and *HDACs* varied among species, with 2.7-, 2.5-, 2.5-, and 3.5-fold as many *BnaHMs*, *BcaHMs*, *BjuHMs*, and *CsHMs* as *AtHMs*, respectively (Figure S1a). There were 47–159 *SDGs*, 7–27 *PRMTs*, 2–8 *HDMAs*, 20–77 *JMJs*, 3–10 *HAGs*, 1–7 *HAMs*, 4–10 *HACs*, 1–4 *HAFs*, 12–40 *HDAs*, 2–8 *SRTs*, and 4–16 *HDTs* in the above Brassicaceae species, respectively (Figure S1b), named according on their chromosomal position in each species (Figure S2).

To elucidate the evolutionary relationships among *HMs*, unrooted phylogenetic trees were constructed. Generally, each type of *HAT*, *HDAC*, *HDM*, and *HMT* shared relatively close relationships in distinct groups, with some exceptions (Figure S3). For example, in terms of *HATs*, all *HACs* were in group a, most *HAGs* were in group b, and *HAFs* and *HAMs* were in groups c and d, respectively (Figure S3-1).

### Conserved domain, structure, and synteny analysis of ***HMs***

Diverse conserved domains were identified in the different *HMs* (Figure S4) and the number of conserved motifs was determined in the *Arabidopsis HMs* (Figure S4-1). Most conserved domains in the *Arabidopsis HMs* were also present in the non-model plants (*B. napus*, *B. carinata*, *B. juncea*, *B. nigra*, *B. oleracea*, *B. rapa*, *C. rubella*, and *C. sativa*). However, several distinct domains were identified in the non-model Brassicaceae *HMs* (Figure S4), including the SHOCT domain in *BjHDA10* and *BjHDT11*, which may bind to itself to perform important functions as an oligomerization domain or bind to other protein domains/motifs and nucleic acids [49]. *BjHDA24* shares a domain with the CYCLIN superfamily, which functions in the cell cycle and transcriptional control (Figure S4-8). In general, each class of *HM* shared a similar gene structure. Of note, several *HMs*, including *BoJMJ23*, *BoSDG24*, and *BcJMJ54*, contained long introns (Figure S4).

To determine the expansion patterns of *HMs*, duplication events within gene pairs were investigated in duplicated blocks of each Brassicaceae genome. In total, 1 176 gene pairs were identified, including 11, 256, 194, 215, 49, 55, 42, 15, and 339 pairs in *(A) thaliana*, *(B) napus*, *B. carinata*, *B. juncea*, *B. nigra*, *B. oleracea*, *B. rapa*, *(C) rubella*, and *C. sativa*, respectively (Figure S5 and Table S2). To understand the potential roles of unknown Brassicaceae *HM* genes, collinearity analysis was performed between *Arabidopsis* and non-model Brassicaceae species. In total, 151, 157, 178, 101, 89, 216, 83, and 109 gene pairs were identified in *A. thaliana-B. carinata*, *A. thaliana-B. juncea*, *A. thaliana-B. napus*, *A. thaliana-B. oleracea*, *A. thaliana-B. rapa*, *A. thaliana-C. sativa*, *A. thaliana-C. rubella*, and *A. thaliana-B. nigra*, respectively (Figure S6 and Table S3).

### Effects of NH4+, salt, B, and Cd on expression patterns of ***BnaHMs***

**Fig. 1:**
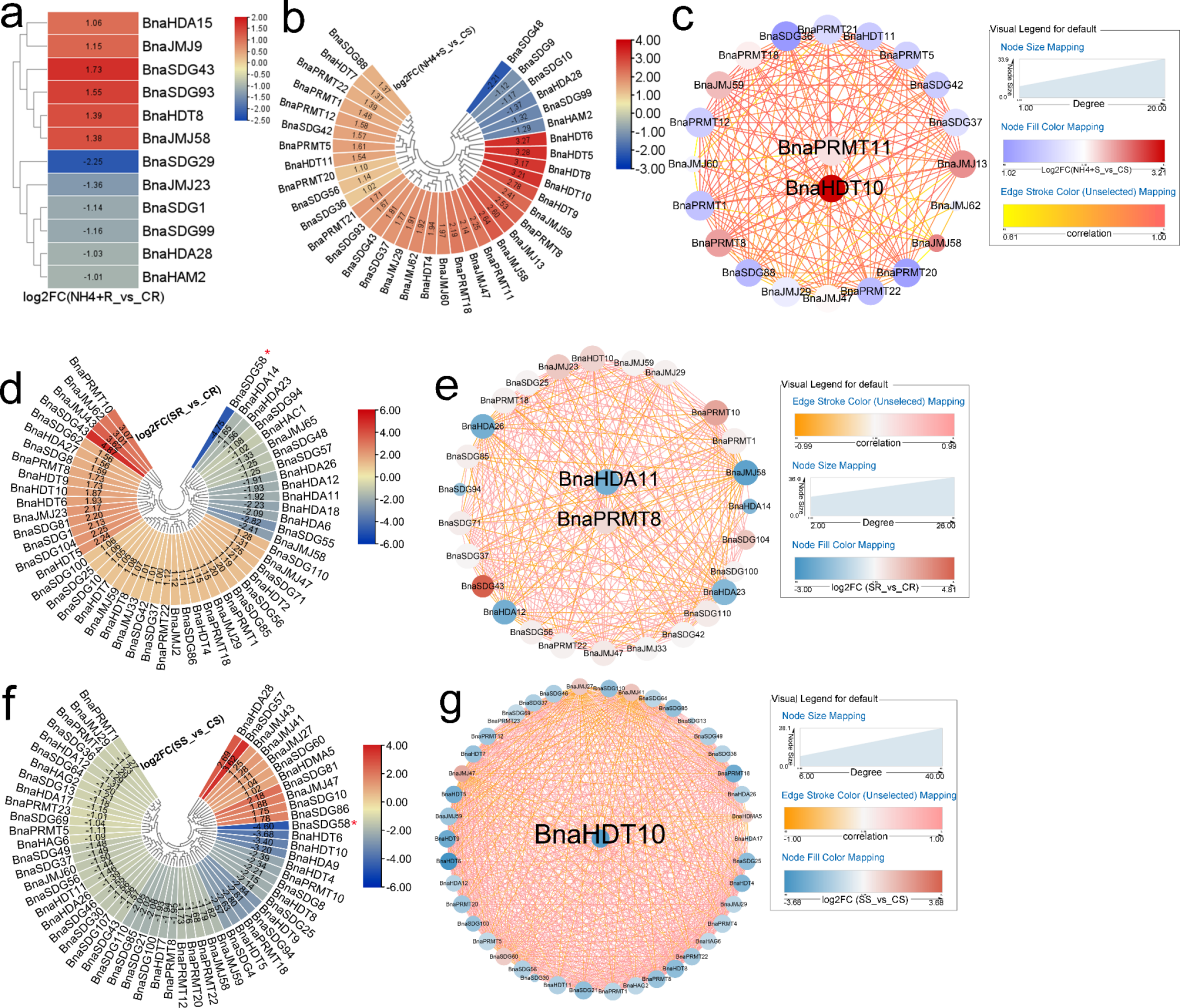
Expression profiles of *BnaHMs* in response to NH_4_^+^ and salt. Cycle nodes represent genes and size of node represents power of the inter-relationship among nodes by degree value; colors of nodes represent log2FC value, red indicates up-regulated genes and blue indicates down-regulated genes; edges between nodes represent correlation. (**a)** Expression analysis of *BnaHMs* in response to NH_4_^+^ toxicity in roots. (**b**) Expression analysis of *BnaHMs* in response to NH_4_^+^ toxicity in shoots. **(c)** Co-expression network analysis of differentially expressed *BnaHMs* in response to NH_4_^+^ toxicity in shoots. **(d)** Expression analysis of *BnaHMs* in response to salt toxicity in roots. **(e)** Co-expression network analysis of differentially expressed *BnaHMs* in response to salt toxicity in roots. **(f)** Expression analysis of *BnaHMs* in response to salt toxicity in shoots. **(g)** Co-expression network analysis of differentially expressed *BnaHMs* in response to salt toxicity in shoots. NH_4_^+^ R: NH_4_^+^-treated roots; CR: control roots; NH_4_^+^ S: NH_4_^+^-treated shoots; CS: control shoots; SR: salt-treated roots; CR: control roots; SS: salt-treated shoots; CS: control shoots; FC: fold-change

Although NH_4_^+^ is the main N source for plants, excess can cause toxicity to crops and reduce grain yields [50, 51]. Here, the expression profiles of *BnaHM* genes were investigated to predict their potential involvement in NH_4_^+^ toxicity resistance. In roots, 12 *BnaHMs* were differentially expressed after excess NH_4_^+^ treatment, half of which were up-regulated (Fig. 1a). In shoots, 37 *BnaHMs* were differentially expressed, six of which showed low levels in the NH_4_^+^-treated group (Fig. 1b). Among these differentially expressed genes (DEGs), based on gene co-expression network analysis (GCNA), *BnaPRMT11* and *HDT10* may be critical genes in response to NH_4_^+^ toxicity (Fig. 1c). In roots, 15 and 38 *BnaHMs* were suppressed and induced by salt treatment, respectively (Fig. 1d), with *BnaHDA11* and *BnaPRMT8* potentially playing roles in salt adaptation (Fig. 1e). In shoots, 48 *BnaHMs*, especially *BnaSDG58*, were markedly regulated by salt exposure (Fig. 1f). According to GCNA, *BnaHDT10* was identified as a hub gene in response to salt stress (Fig. 1g).

**Fig. 2:**
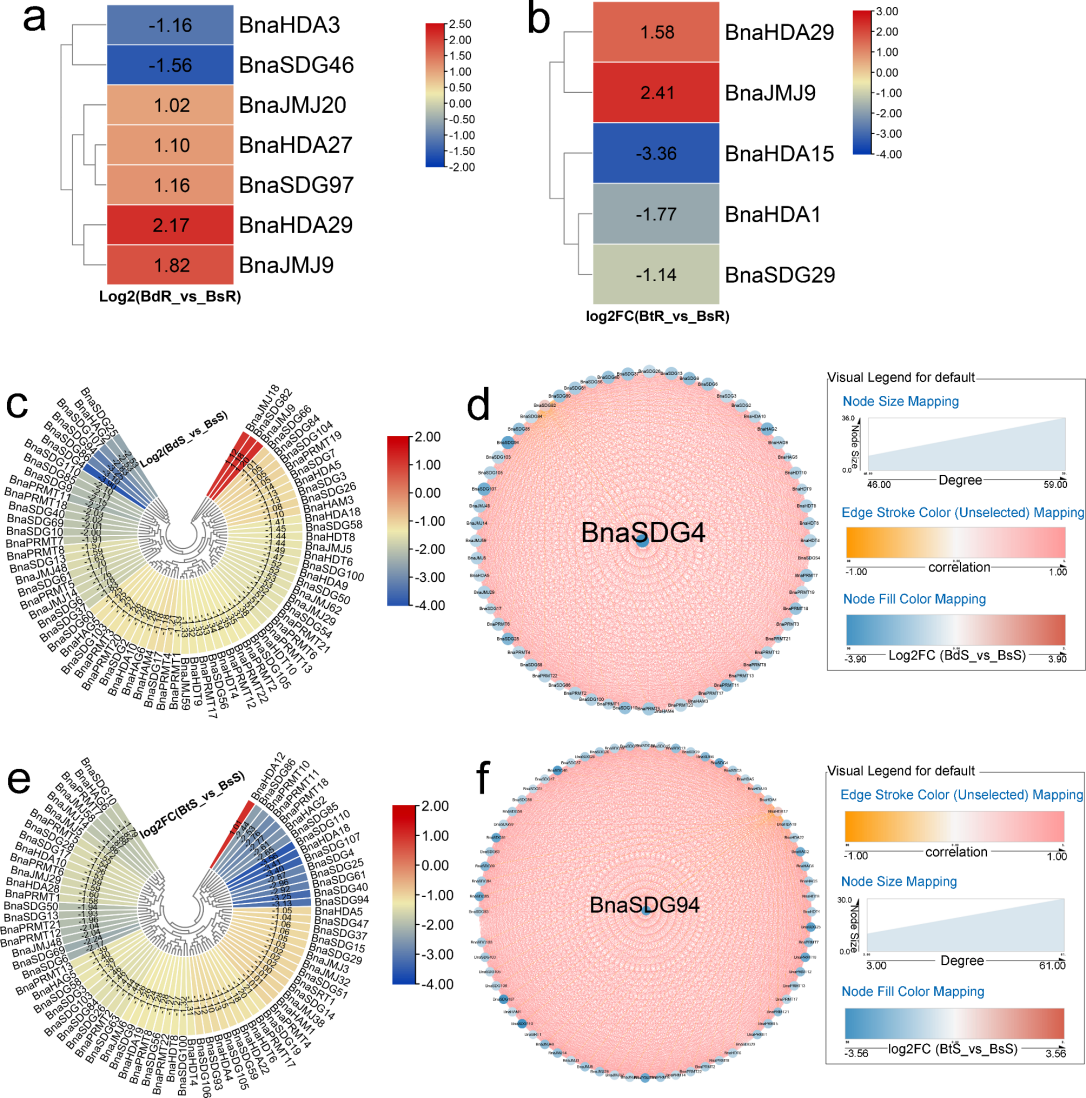
Expression profiles of *BnaHM* genes in response to low and excess B. Cycle nodes represent genes and size of node represents power of the inter-relationship among nodes by degree value; colors of nodes represent log2FC value; red indicates up-regulated genes and blue indicates down-regulated genes; edges between nodes represent correlation. (**a**) Expression analysis of *BnaHMs* under low and normal B supply levels in roots. (**b**) Expression analysis of *BnaHMs* under excess and normal B supply levels in roots. (**c**) Expression analysis of *BnaHMs* under low and normal B supply levels in shoots. (**d**) Co-expression network analysis of differentially expressed *BnaHMs* under low and normal B supply levels in shoots. (**e**) Expression analysis of *BnaHMs* under excess and normal B supply levels in shoots. (**f**) Co-expression network analysis of differentially expressed *BnaHMs* under excess and normal B supply levels in shoots. BdR: low B-treated roots; BsR: control roots; BdS: low B-treated shoots; BsS: control shoots; BtR: excess B-treated roots; BtS: excess B-treated shoots; FC: fold-change

Both B deficiency and toxicity can have adverse effects on plant growth and development [52]. However, whether *BnaHMs* are involved in B-mediated plant growth is unclear. Our results identified several *BnaHMs* that were differentially expressed after B treatment (Fig. 2). In roots, *BnaHDA3* and *BnaSDG46* were inhibited by B deficiency, while five *BnaHMs* were induced (Fig. 2a). B toxicity also altered the expression patterns of *BnaHMs* (Fig. 2b, e). In the B deficiency group, *BnaJMJ18*, *BnaSDG82*, and *BnaJMJ9* were up-regulated in shoots, while 69 *BnaHMs* were down-regulated (Fig. 2c). *BnaSDG4* was identified as a key gene (Fig. 2d). In shoots, only *BnaHDA12* increased in response to excess B, while the remaining *BnaHMs* were reduced (Fig. 2e). Among them, *BnaSDG94* was identified as a potential hub gene (Fig. 2f).

**Fig. 3:**
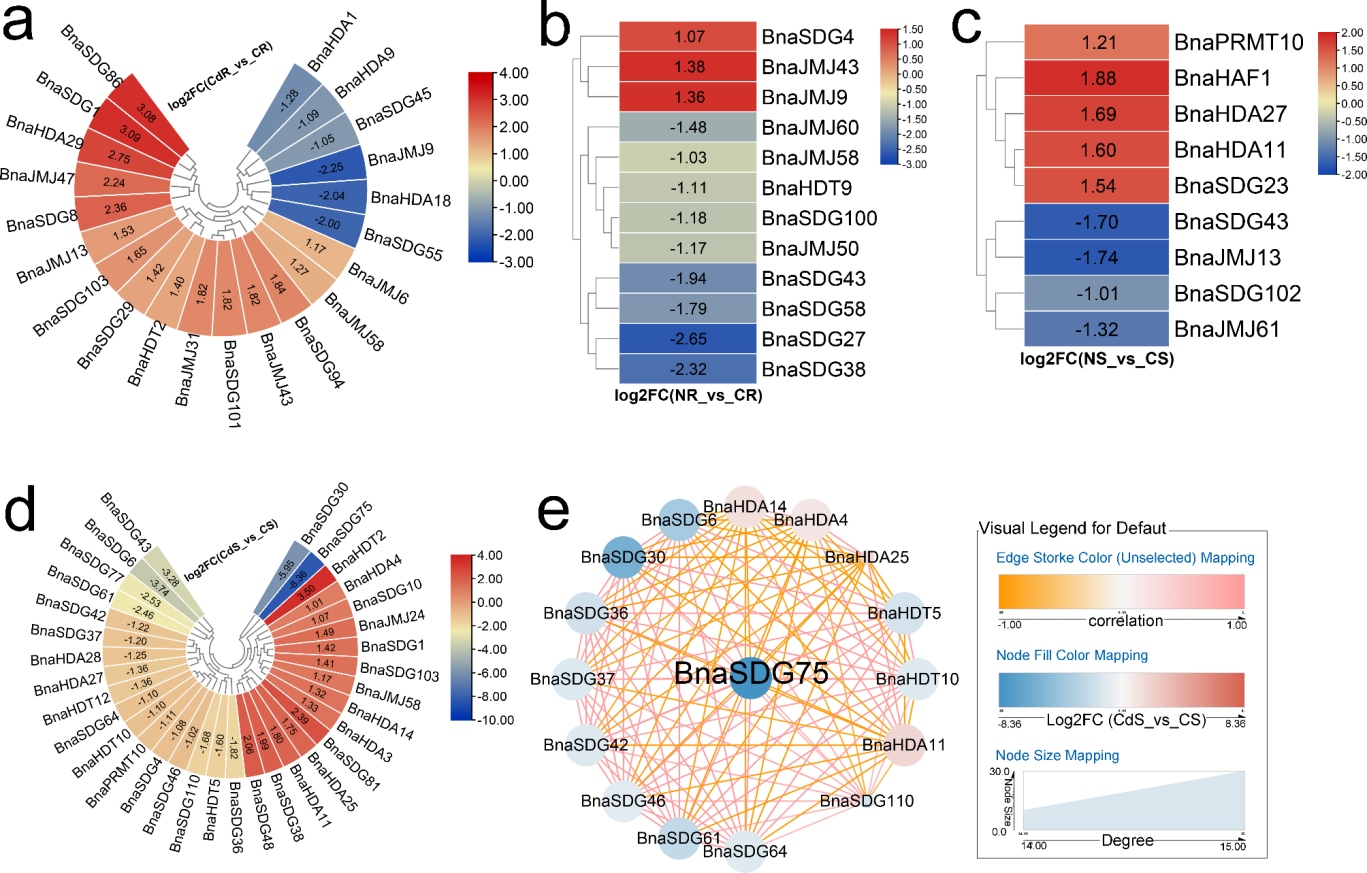
Expression profiles of *BnaHMs* in response to Cd toxicity and N shortage. Cycle nodes represent genes and size of node represents power of the inter-relationship among nodes by degree value; colors of nodes represent log2FC value; red indicates up-regulated genes and blue indicates down-regulated genes; edges between nodes represent correlation. (**a**) Expression analysis of *BnaHMs* in response to Cd toxicity in roots. (**b**) Expression analysis of *BnaHMs* in response to N shortage in roots. (**c**) Expression analysis of *BnaHMs* in response to N shortage in shoots. (**d**) Expression analysis of *BnaHMs* in response to Cd toxicity in shoots. (**e**) Co-expression network analysis of differentially expressed *BnaHMs* in response to Cd toxicity in shoots. CdR: Cd-treated roots; CR: control roots; CdS: Cd-treated shoots; CS: control shoots; NR: N-treated roots; CR: control roots; NS: N-treated shoots; CS: control shoots; FC: fold-change

Cd is a non-essential heavy metal toxic for plant growth [53]. In roots, 15 and six *BnaHMs* exhibited higher and lower expression, respectively, in the Cd-treated group compared with the control group (Fig. 3a). In shoots, *BnaSDG30* and *BnaSDG75* were significantly inhibited by Cd, while *BnaHDT2* was induced (Fig. 3d). *BnaSDG75* was also identified as a key gene in the co-expression network (Fig. 3e).

### Effects of N, K, and P on expression patterns of ***BnaHMs***

As an essential macronutrient, N is required for rapeseed growth and development [54]. To investigate the response of *BnaHMs* to N limitation, we identified their expression profiles. *BnaSDG4*, *BnaJMJ9*, and *BnaJMJ43* were up-regulated in the N-treated roots, while nine other genes were down-regulated (Fig. 3b). In shoots, *BnaPRMT10*, *BnaHAF1*, *BnaHDA27*, *BnaHDA11*, and *BnaSDG23* were substantially induced by N deficiency, while *BnaSDG43*, *BnaJMJ13*, *BnaSDG102*, and *BnaJMJ61* were repressed (Fig. 3c).

**Fig. 4:**
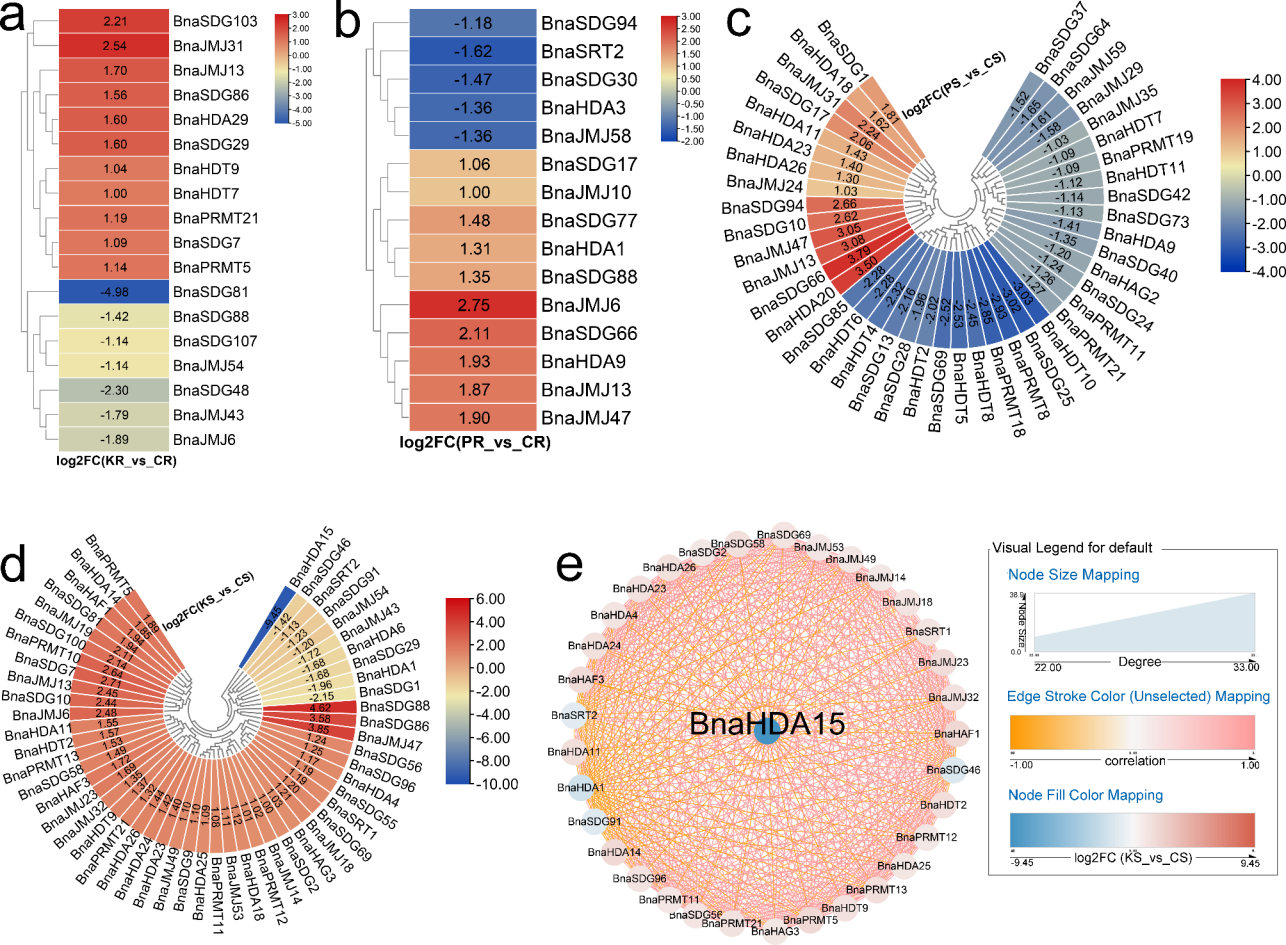
Expression profiles of *BnaHMs* in response to K and P starvation. Cycle nodes represent genes and size of node represents power of the inter-relationship among nodes by degree value; colors of nodes represent log2FC value; red indicates up-regulated genes and blue indicates down-regulated genes; edges between nodes represent correlation. (**a**) Expression analysis of *BnaHMs* in response to K starvation in roots. (**b**) Expression analysis of *BnaHMs* in response to P starvation in roots. (**c**) Expression analysis of *BnaHMs* in response to P starvation in shoots. (**d**) Expression analysis of *BnaHMs* in response to K starvation in shoots. (**e**) Co-expression network analysis of differentially expressed *BnaHMs* in response to K starvation in shoots. KR: K-treated roots; CR: control roots; KS: K-treated shoots; CS: control shoots; PR: P-treated roots; CR: control roots; PS: P-treated shoots; CS: control shoots; FC: fold-change

Previous studies have shown that K can also cause stress to plants [55, 56]. Our results showed that limited K induced 11 *BnaHMs* and suppressed seven *BnaHMs* in the roots, especially *BnaSDG81* (Fig. 4a). In shoots, 10 *BnaHMs* (e.g., *BnaHDA15*, *BnaSDG46*, and *BnaSDG1*) were decreased after K treatment, while 52 *BnaHMs*, especially *BnaJMJ47*, *BnaSDG86*, and *BnaSDG88*, were increased (Fig. 4d). *BnaHDA15* was identified as a key gene according to GCNA (Fig. 4e). Given its close involvement in photosynthesis, P is an essential nutrient for plant growth and development [57]. Here, in response to P stress, the expression levels of several *BnaHMs*, especially *BnaJMJ6*, increased in roots, whereas five *BnaHMs* were markedly suppressed (Fig. 4b). In shoots, 14 *BnaHMs* showed higher expression levels after P treatment, while 29 were inhibited by P stress (Fig. 4c).

### Identification of weighted gene co-expression network analysis (WGCNA) modules and hub genes associated with target traits

All genes in the RNA sequencing (RNA-seq) data, not just DEGs, were analyzed for significant associations with phenotypes using WGCNA based on previous methods [58]. WGCNA was established to analyze hub genes in response to A, salt, Cd, N, and K stress.

**Fig. 5:**
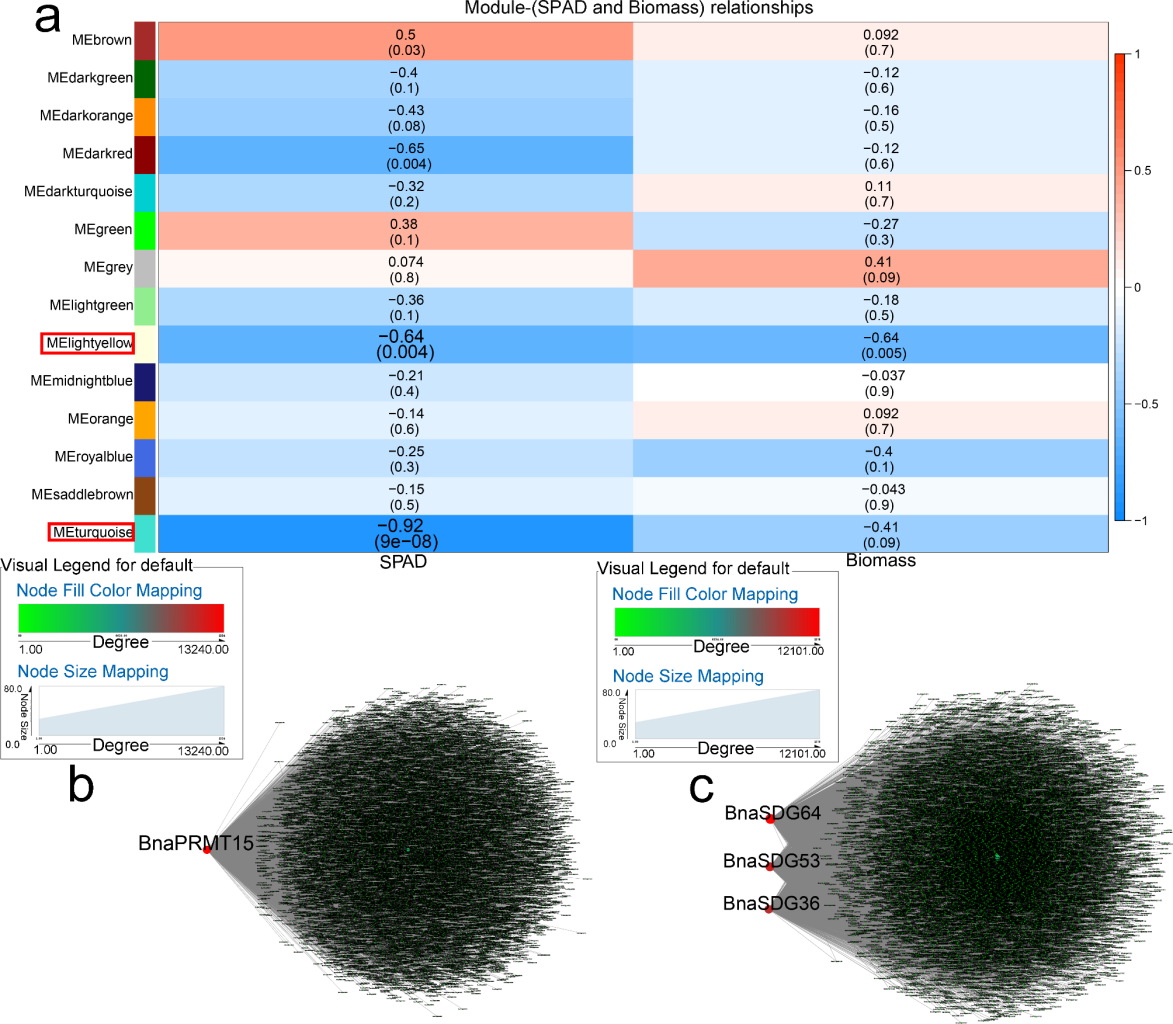
WGCNA of rapeseed genes in response to NH4 + toxicity. (**a**) Module-trait correlation showing significance of module eigengene correlation with trait (SPAD and biomass). Left panel shows modules. (**b**) Cytoscape representation of relationship of *BnaHMs* in “lightyellow” module. (**c**) Cytoscape representation of relationship of *BnaHMs* in “turquoise” module. Key genes are represented by large red circles

The “lightyellow” (r = -0.64, *p* < 0.01) and “turquoise” (r = -0.92, *p* < 0.01) modules were negatively correlated with chlorophyll content (SPAD) after NH_4_^+^ toxicity treatment (Fig. 5a). Two co-expression networks were constructed to identify core genes. In the “lightyellow” and “turquoise” modules, *BnaPRMT15*, and *BnaSDG64*, *BnaSDG53*, and *BnaSDG36* were respectively identified in response to NH_4_^+^ exposure (Fig. 5b, c). In total, 37 genes in the “lightyellow” module and 40 genes in the “turquoise” module are involved in various stresses, such as oxidative stress, and interact with core genes (Table S5-1).

**Fig. 6:**
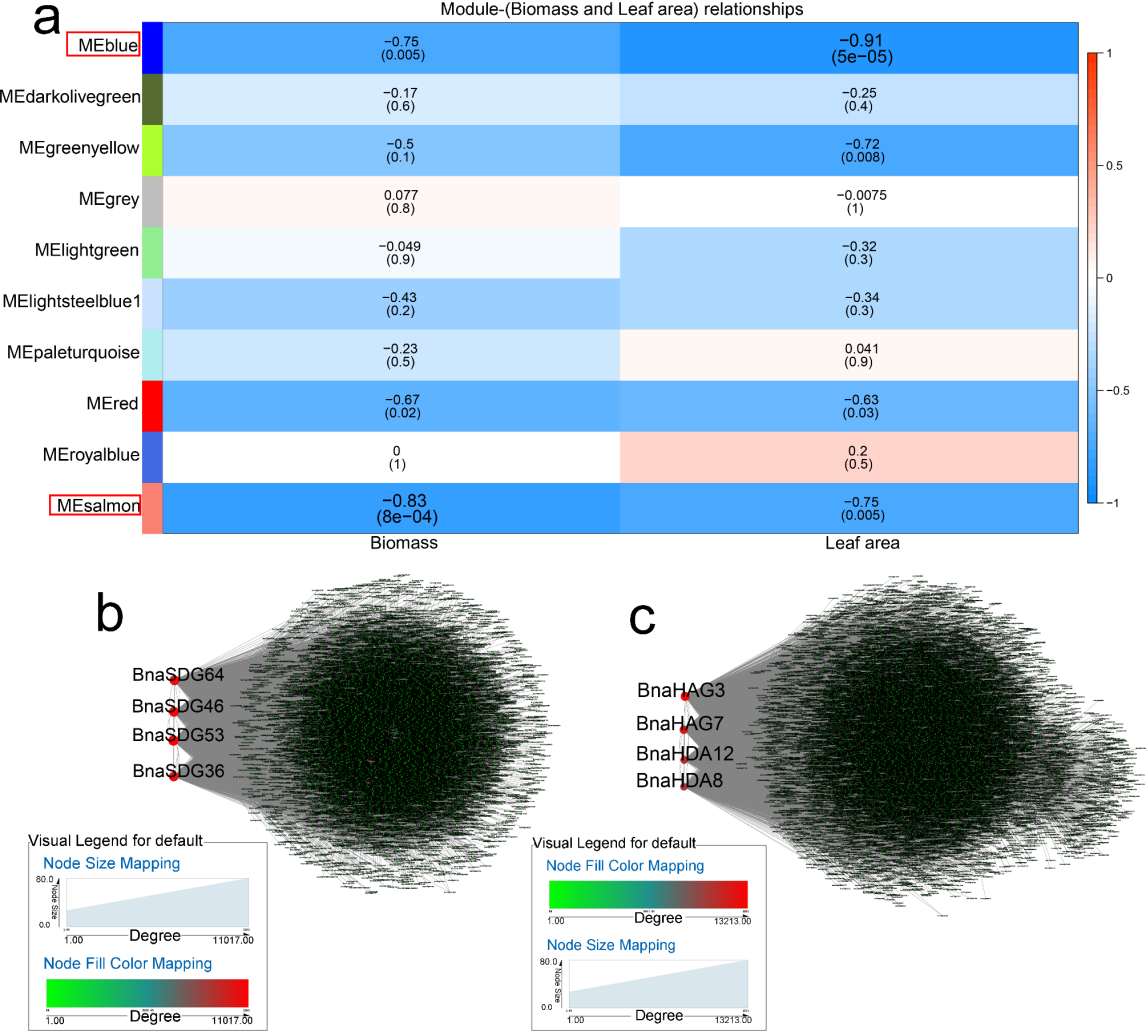
WGCNA of rapeseed genes in response to salt. (**a**) Module-trait correlation showing significance of module eigengene correlation with trait (biomass and leaf area). Left panel shows modules. (**b**) Cytoscape representation of relationship of *BnaHMs* in “blue” module. (**c**) Cytoscape representation of relationship of *BnaHMs* in “salmon” module. Key genes are represented by large red circles

WGCNA was also performed to evaluate the relationship between modules and salinity (Fig. 6). The “salmon” (r = -0.83, *p* < 0.05) and “blue” (r = -0.91, *p* < 0.05) modules were negatively correlated with biomass and leaf area, respectively (Fig. 6a). Four *BnaHMs* (*BnaSDG53*, *BnaSDG36*, *BnaSDG46*, and *BnaSDG64*) were identified as important genes in the “blue” module (Fig. 6b). *BnaHAG3*, *BnaHDA12*, *BnaHDA8*, and *BnaHAG7* were identified as hub genes in the “salmon” module (Fig. 6c). In addition, seven and nine genes in the “salmon” and “blue” modules, respectively, were salt-responsive and associated with core genes (Table S5-2).

**Fig. 7:**
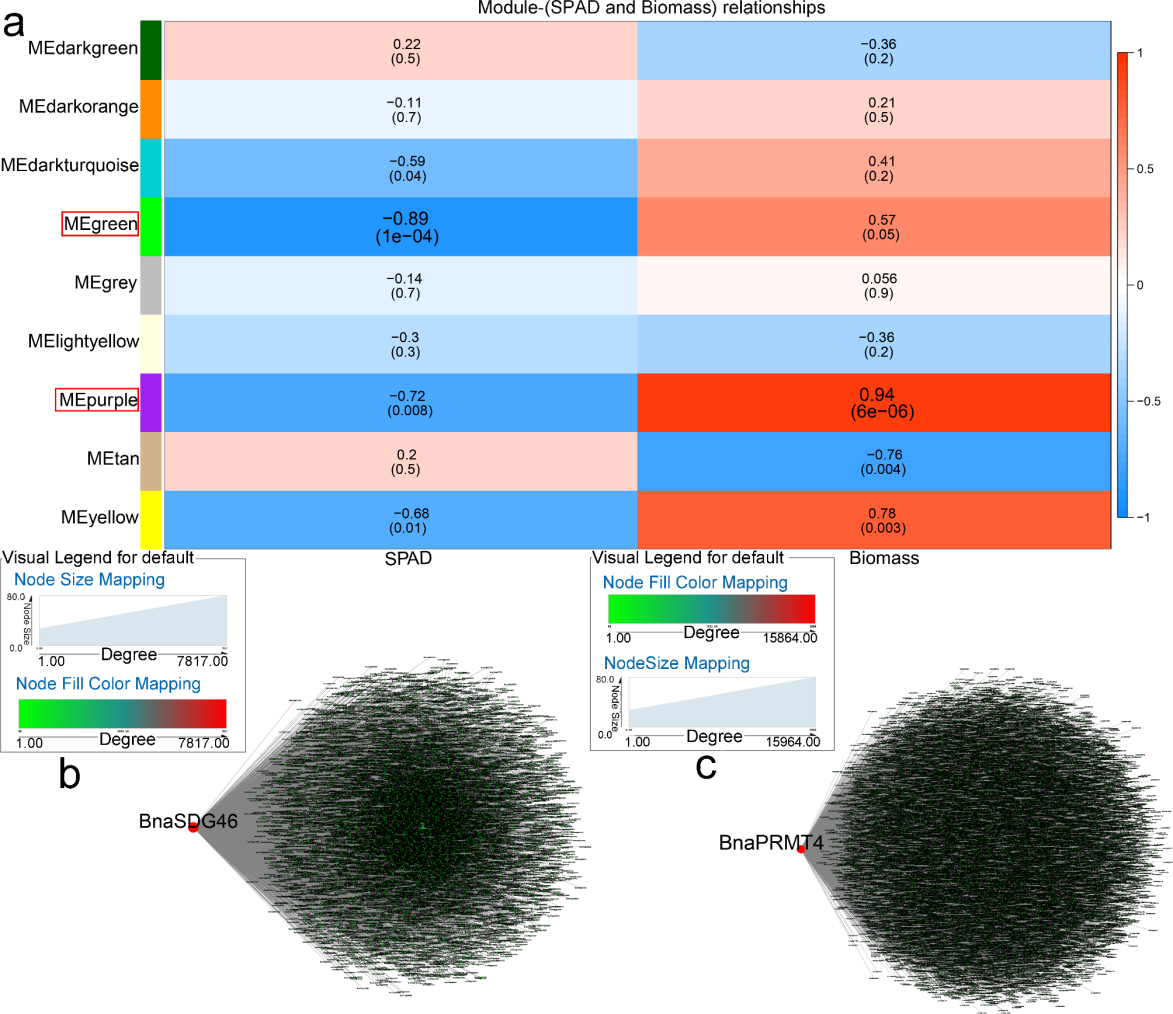
WGCNA of rapeseed genes in response to Cd stress. (**a**) Module-trait correlation showing significance of module eigengene correlation with trait (SPAD and biomass). Left panel shows modules. (**b**) Cytoscape representation of relationship of *BnaHMs* in “green” module. (**c**) Cytoscape representation of relationship of *BnaHMs* in “purple” module. Key genes are represented by large red circles

Using WGCNA, core genes associated with Cd stress were identified. As shown in Fig. 7, both “green” and “purple” module were negatively correlated with SPAD and positively correlated with biomass (Fig. 7a). “Yellow” module was too, while “dark turquoise” was negatively correlated with SPAD and “purple” was positively correlated with biomass (Fig. 7a). Gene interaction networks were established for these two modules, and two key genes were identified (*BnaSDG46* and *BnaPRMT4*, respectively) (Fig. 7b, c). In both modules, several Cd-resistance genes were identified and were associated with core genes (Table S5-3).

**Fig. 8:**
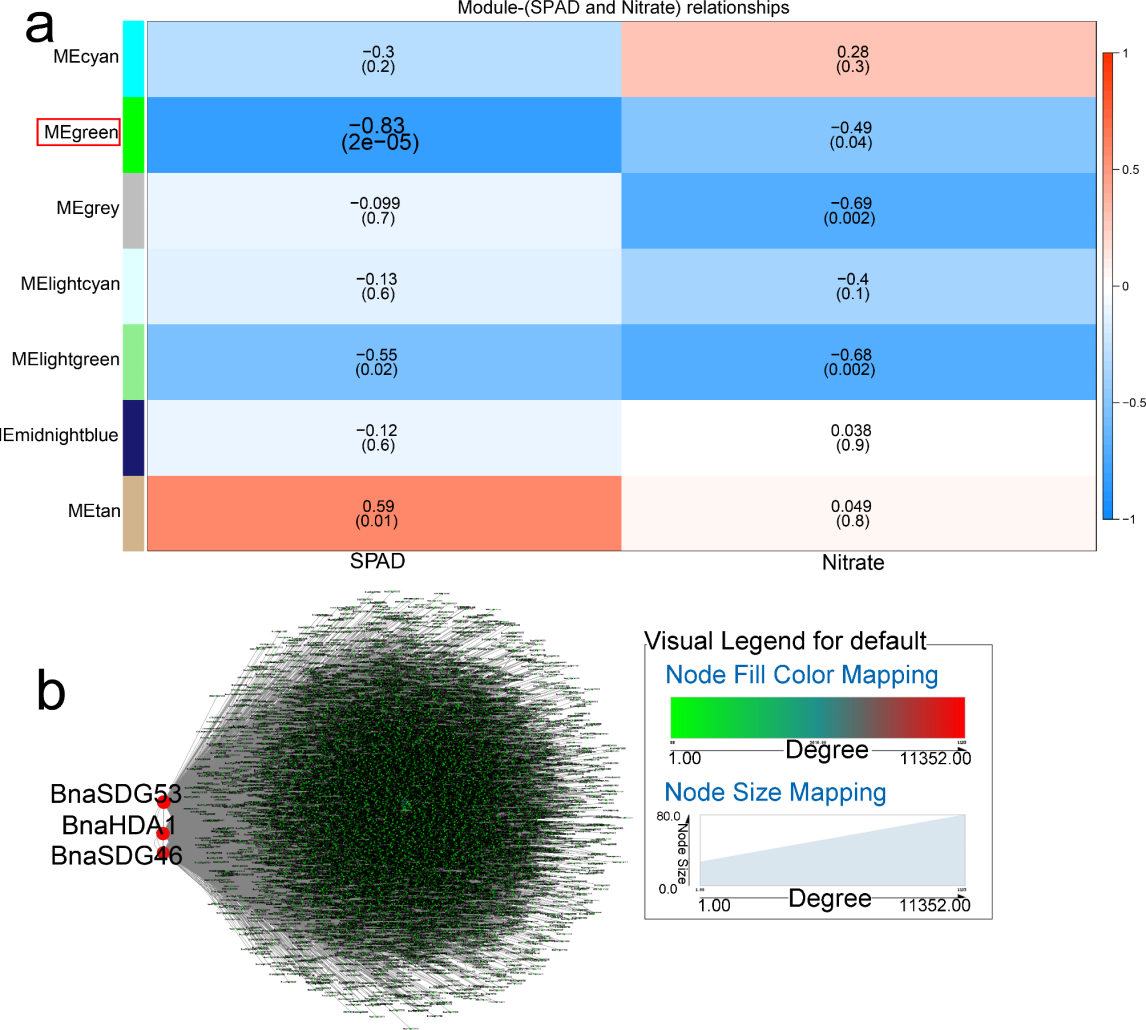
WGCNA of rapeseed genes in response to N starvation. (**a**) Module-trait correlation showing significance of module eigengene correlation with trait (SPAD and Nitrate). Left panel shows modules. (**b**) Cytoscape representation of relationship of *BnaHMs* in “green” module. Key genes are represented by large red circles

The relationship between WGCNA modules and N shortage was also explored. All genes were clustered into seven modules, and genes in the “green” module (r = -0.83, *p* < 0.05) were significantly correlated with SPAD (Fig. 8a). Three hub genes (*BnaSDG53*, *BnaHDA1*, and *BnaSDG46*) were screened from co-expression gene network mapping (Fig. 8b) and may play additional roles in adaptation to various stresses. Furthermore, several genes in the “green” module play roles in stress adaptation and interact with the three hub genes (Table S5-4).

**Fig. 9:**
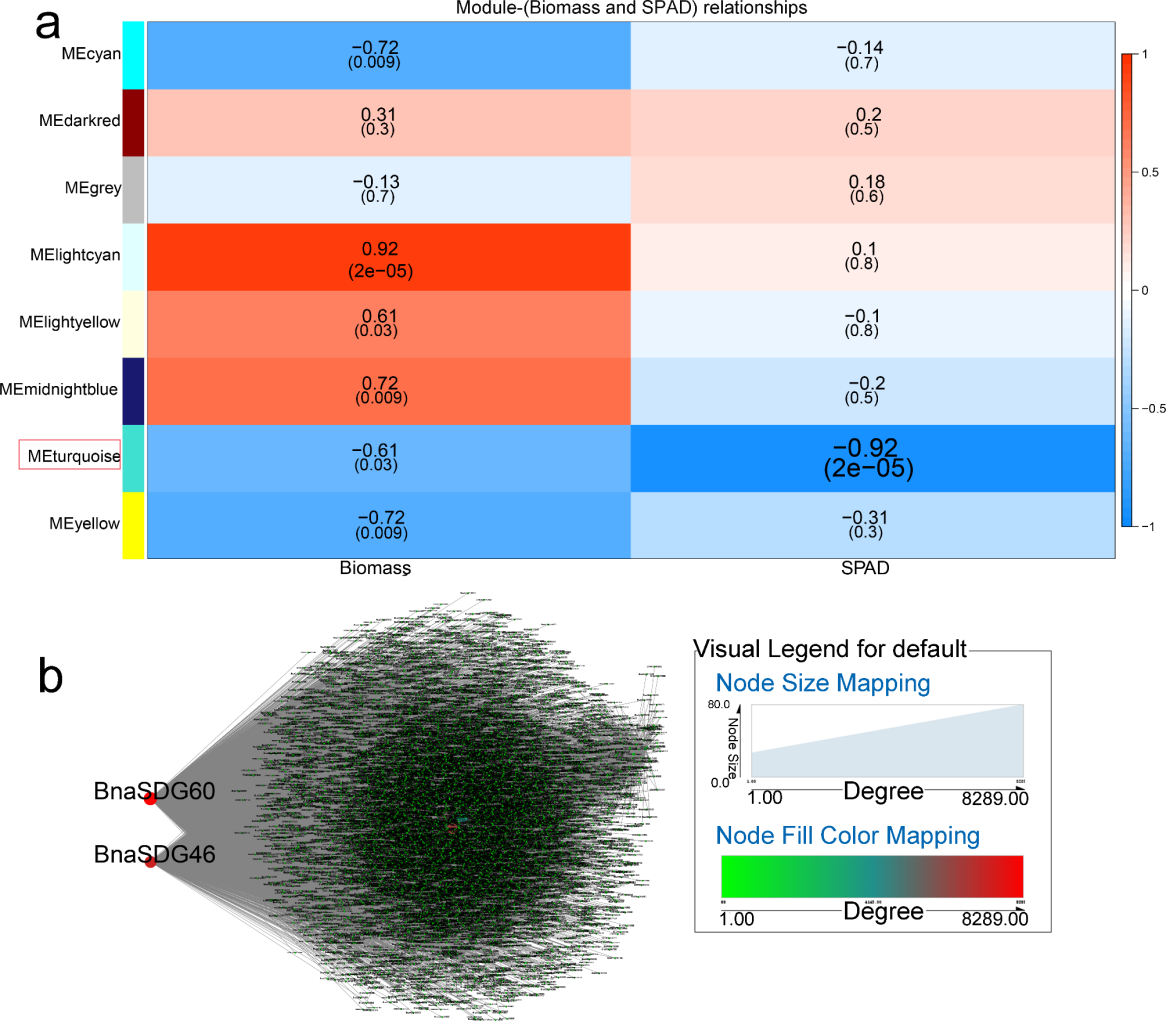
WGCNA of rapeseed genes in response to K starvation. (**a**) Module-trait correlation showing significance of module eigengene correlation with trait (biomass and SPAD). Left panel shows modules. (**b**) Cytoscape representation of relationship of *BnaHMs* in “turquoise” module. Key genes are represented by large red circles

In response to K stress, eight WGCNA modules were obtained. The “turquoise” module (r = -0.92, *p* < 0.05) showed a negative correlation with SPAD (Fig. 9a). *BnaSDG60* and *BnaSDG46* were identified as critical genes in this module (Fig. 9b). In addition, stress-related and K-transport genes in the ‘turquoise” module were associated with *BnaSDG60* and *BnaSDG46* (Table S5-5).

### Diverse responses of ***BnaHMs*** to nutrient stresses

**Fig. 10:**
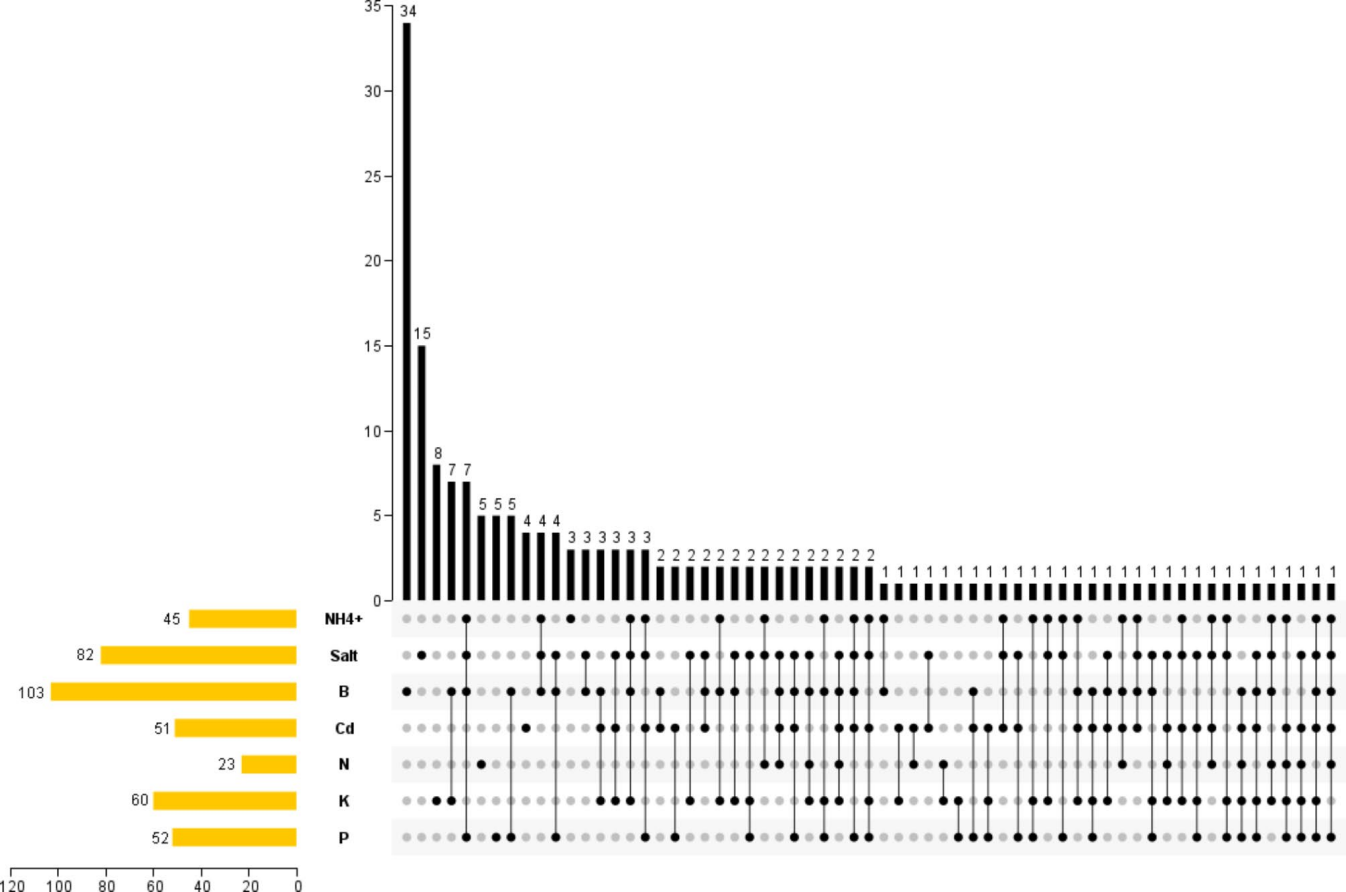
Venn diagram showing transcriptional responses of *BnaHMs* to diverse stresses. The number of differentially expressed *BnaHMs* of Brassica napus under diverse nutrient stresses

To investigate whether *BnaHMs* responded to diverse stresses simultaneously, we constructed a Venn diagram. Results showed that most *BnaHMs* were affected by more than one stress (Fig. 10 and Table S4). For example, 27 *BnaHMs* were simultaneously under the control of two stresses; 31 *BnaHMs* simultaneously responded to three stress signals; 32 *BnaHMs* simultaneously responded to four stresses; 11 *BnaHMs* were controlled by five stresses; and two genes responded to six stresses.

## Discussion

*HMs* play essential roles in plant growth and stress responses and have been successfully identified in many plants, such as *Arabidopsis*, wheat, and maize [59]. However, information on Brassicaceae *HMs* remains limited. In this study, we systematically characterized *HMs* in nine Brassicaceae species and identified 1 798 *HMs*, including 102 *AtHMs*, 280 *BnaHMs*, 251 *BcHMs*, 251 *BjHMs*, 144 *BnHMs*, 155 *BoHMs*, 137 *BrHMs*, 122 *CrHMs*, and 356 *CsHMs*. We further analyzed their phylogeny, conserved domains, gene structure, and synteny, as well as their expression profiles in response to NH_4_^+^, B, salt, Cd, N, P, and K stress. These results will contribute to a comprehensive understanding of Brassicaceae *HM* genes.

### Comparison of ***HMs*** among nine Brassicaceae species

We identified 280, 251, 251, 144, 155, 137, 122, and 356 *HMs* in *B. napus*, *B. carinata*, *B. juncea*, *B. nigra*, *B. oleracea*, *B. rapa*, *Capsella rubella*, and *Camelina sativa*, respectively (Figure S1 and Table S1). We also found significantly more *BnaHMs*, *BcHMs*, *BjHMs*, *BnHMs*, *BoHMs*, *BrHMs*, *CrHMs*, and *CsHMs* than *AtHMs* (2.7-, 2.4-, 2.4-, 1.4-, 1.5-,1.3-, 1.1-, and 3.4-fold higher, respectively) (Figure S1 and Table S1). Orthologous *HMs* were found based on synteny analysis. We identified 11 *AtHM*, 256 *BnaHM*, 194 *BcHM*, 215 *BjHM*, 49 *BnHM*, 55 *BoHM*, 42 *BrHM*,15 *CrHM*, and 339 *CsHM* pairs (Figure S5 and Table S2). Results showed that more segmental duplications of *HMs* were found in non-model Brassicaceae species than in *Arabidopsis*, which may induce the expression of non-model Brassicaceae *HMs*. Whole-genome replication is known to occur in *B. napus*, *B. carinata*, *B. juncea*, and *Camelina sativa* [60–64]. Therefore, segmental and whole-genome duplications may have contributed to the expansion and evolution of *HMs* in the above species.

Synteny analysis between duplicated blocks of *Arabidopsis-B. carinata*, *Arabidopsis-B. juncea*, *Arabidopsis-B. napus*, *Arabidopsis-B. oleracea*, *Arabidopsis-B. rapa*, *Arabidopsis-C.sativa*, *Arabidopsis-C. rubella*, and *Arabidopsis-B. nigra* was also performed, yielding 151, 157, 178, 101, 89, 216, 83 and 109 gene pairs, respectively (Figure S6 and Table S3). These gene pairs are considered to have originated from common ancestors with *AtHMs* [60–64], suggesting that they may have similar functions to the corresponding *Arabidopsis* genes. Thus, the functions of non-model Brassicaceae *HMs* were predicted based on homologous *Arabidopsis HMs*. Several *AtHMs* are involved in stress responses [10, 65, 66]. Although many unknown non-model Brassicaceae *HMs* could be inferred from orthologous *Arabidopsis* genes, these comparisons require further experiments.

Conserved domains are associated with gene function [67]. We identified typical domains in the *HMs* (Figure S4). Most Brassicaceae *HMs* with conserved domains shared similar functions, but several distinct domains were identified in several non-model Brassicaceae *HMs*, such as the FYVE_like_SF superfamily domain in *BnaJMJ65*, which plays an important role in vesicular traffic and signal transduction (Figure S4-2). Novel functions may be predicted from unique domains, and thus greater attention should be paid to genes with special elements in the future.

### Putative functions of ***BnaHMs*** in stress response

*HMs* are important in plant defense. Here, the expression patterns of *BnaHMs* were determined to explore their function under various stresses. In roots and shoots, 79 and 81 *BnaHMs* were up-regulated or down-regulated by B deficiency and toxicity (Fig. 2) and *BnaHM* expression patterns were changed by NH_4_^+^ and N deficiency (Figs. 1a-c and 3b-c). More than 50 *BnaHMs* showed differential expression in response to P shortage, and many *BnaHMs* were influenced by K deficiency stress (Fig. 4). These findings indicate that *BnaHMs* play essential roles in the stress response. Various abiotic stresses, including drought, salinity, and cold, adversely affect plant growth and development. *HMs* share important roles in regulating stress adaptation. For example, *AtHDA6* and *AtHDA19* are involved in ABA responses and are required for salt tolerance [59, 68]. Here, many *BnaHM*s responded to Cd and salt stress, with altered expression in the roots or shoots (Figs. 1d-g and 3d-e). These findings suggest that *BnaHMs* and methylation play essential roles in rapeseed resistance to diverse stresses.

Candidate *BnaHMs* were determined through DEG co-expression analysis and WGCNA. *BnaPRMT15*, *BnaSDG36*, *BnaSDG53*, *BnaSDG64*, *BnaHDT10*, and *BnaPRMT11* were identified in response to NH_4_^+^ toxicity (Figs. 1c and 5). *BnaHDA11*, *BnaHDT10*, *BnaPRMT8*, *BnaHAG3*, *BnaHAG7*, *BnaSDG36*, *BnaSDG46*, *BnaSDG53*, *BnaHDA12*, *BnaHDA8*, and *BnaSDG64* were associated with plant survival under salt stress (Fig. 1e and g, and Fig. 6). *BnaPRMT4*, *BnaSDG46*, and *BnaSDG75* were identified as Cd-related genes (Figs. 3e and 7). *BnaSDG4* and *BnaSDG94* were identified as B stress candidate genes (Fig. 2d and f). *BnaSDG46*, *BnaSDG53*, and *BnaHDA1* were identified as N-deficiency candidate genes (Fig. 8). *BnaHDA15*, *BnaSDG46*, and *BnaSDG60* were identified as K limitation-related genes (Fig. 9). Based on orthologous gene analysis, the ortholog of *AtHDA6*, which responds to drought stress [69], was identified as *BnaHDA8*, and the ortholog of *AtHDA14*, which functions in regulating stress responses [70–74], was identified as *BnaHDA1*. In addition, according to WGCNA, several downstream genes identified in modules that may be involved in various stresses, such as low temperature and salt, interacted with the core genes, indicating that these core genes may participate in stress tolerance by interacting with downstream stress-related genes (Table S5). These results suggest that *HMs* play an important role in stress response. As such, future studies should pay attention to the above candidate genes.

This study also found that many differentially expressed *BnaHMs* responded to different stresses at the same time (Table S4). For example, two *BnaHMs* (*BnaSDG10* and *BnaJMJ58*) were simultaneously regulated by six stresses, and 11 *BnaHMs* (e.g., *BnaHDT10*, *BnaSDG46*, *BnaPRMT10*) were simultaneously regulated by five stresses. However, certain genes were only impacted by a single stress signal, implying that many *BnaHMs* may participant in different stresses, while others only play a core role under a specific stress.

Previous studies have shown that several *HMs* in rice may participate in stress adaptations. For example, *OsHDT701* and *OsHDT702* in rice are repressed by drought and salt simultaneously [75, 76]. Here, several key genes identified by co-expression analysis or WGCNA also responded to more than three different types of stress. For instance, *BnaPRMT11*and *BnaHDA1* were differentially expressed under four and five types of stress, respectively (Fig. 10 and Table S4) and *BnaSDG10* and *BnaJMJ58* simultaneously responded to six different stresses. The salt stress-correlated core gene *BnaHDT10* also responded to four other stresses (i.e., A, B, Cd, and P). In addition, *BnaSDG46* was identified as a salt-, B-, Cd- and K-related key gene by WGCNA. These results suggest that the above hub *BnaHMs* may play critical roles in resistance to multiple stressors, and that they may show different functions under different stress. Therefore, future studies should focus on the potential functions of these genes.

## Methods

### ***HM*** gene identification, phylogenetic relationship, chromosomal location, conserved domains, gene structure, and synteny

Known *AtHM* protein sequences were used as queries and the *B. napus*, *B. carinata*, *B. juncea*, *B. nigra*, *B. oleracea*, *B. rapa*, *C. rubella*, and *C. sativa* protein databases were searched using “Blast Several Sequences to a Big Database” in TBtools [77] with an e-value of e^-5^. After aligning the full-length protein sequences by ClustalW with default parameters, MEGA X was used to construct the phylogenetic tree with the maximum-likelihood method [78].

Using chromosome length and gene position files, the chromosomal distributions of *HMs* were acquired and visualized using “Gene Location Visualize (Advanced)” in TBtools. The conserved domains in *HMs* were confirmed using the Batch Web CD-Search Tool (https://www.ncbi.nlm.nih.gov/Structure/bwrpsb/bwrpsb.cgi) [77]. The conserved domains were visualized using “Visualize NCBI CDD Domain Pattern” in TBtools [77]. The Visualize Gene Structure (Basic) tool was used to draw the gene structure map based on generic feature format v3 (gff3) files of the *HMs*.

We used “One Step MCScanX” in TBtools to analyze *HM* duplication events with genome sequences and gff3 files. “Table Row Extract or Filter”, “File Transformat for Microsynteny Viewer and Advanced Circos”, “Fasta stats”, and “File Merge for MCScanX” in TBtools were used to visualize the syntenic relationships of *HM* genes based on previous studies [77].

### Transcriptome analysis, GCNA, and WGCNA of ***BnaHMs***

The transcriptome data can be found in previously published papers [79–83]. All data required to reproduce these findings can be obtained by contacting the correlation authors. Fastp software (v0.20.1) was used to evaluate the overall sequencing quality of the raw reads and low-quality reads were removed. Alignment of high-quality reads with *B. napus* reference genome sequences (http://cbi.hzau.edu.cn/cgi-bin/rape/download_ext, accessed on 15 May 2022) was performed using Hisat2 (v2.1.0) and SAMtools (v1.6) software. Stringtie (v1.3.3b) was used to calculate the expression levels of high-confidence genes in each sample. The R package “edgeR”, with *p* < 0.05, false-discovery rate (FDR) < 0.05, and |log2(fold-change)| ≥ 1, was used to define DEGs. GCNA was performed using the cor.test function in R (v4.1), and network visualized using Cytoscape (v3.8.2, https://cytoscape.org/download.html, accessed on 13 April 2022) [56]. The R WGCNA package (v1.51) was used to complete WGCNA with high-quality genes. Significant module-trait relationships with target traits were determined by calculating modular trait gene values. Gene co-expression network maps were generated using Cytoscape (v3.8.2, https://cytoscape.org/download.html, accessed on 13 April 2022). The gene with high | log2 (a fold - change) | and degree are selected as hub gene, and was placed in the middle of the network.

### Plant materials and treatments

Uniform 7-day-old *B*. *napus* (Zhongshuang 11) seedings were transplanted into black plastic containers containing Hoagland nutrient solution (5.0 mM KNO_3_, 1.0 mM KH_2_PO_4_, 2.0 mM MgSO_4_·7H_2_O, 5.0 mM Ca (NO_3_)_2_·4H_2_O, 0.10 µM Na_2_MoO_4_·2H_2_O, 0.050 mM EDTA-Fe, 0.80 µM ZnSO_4_·7H_2_O, 9.0 µM MnCl_2_·4H_2_O, 0.30 µM CuSO_4_·5H_2_O, and 46 µM H_3_BO_3_). Before treatments, the *B. napus* seedlings were cultivated for 10 days (d) in a chamber under 25 °C daytime/22°C night-time temperature, 300–320 µmol m^− 2^ s^− 1^ light intensity, 16-h light/8-h dark photoperiod, and 70% relative humidity. **B deficiency and toxicity treatments**: We cultivated 17-day-old seedlings for 10 d in B-deficient (0.25 µM H_3_BO_3_) and B-excess (1 500 µM H_3_BO_3_) treatment groups; **N, P, and K depletion treatments**: We cultivated 17-day-old *B. napus* seedlings in Hoagland nutrient solution (consisting of 0.30 mM N, 5 mΜ P, and 0.30 mM K) for 3 d; **NH**^**4+**^**toxicity treatment**: We cultivated 17-day-old uniform Zhongshuang 11 seedlings in Hoagland nutrient solution (consisting of normal nitrate) for 10 d, followed by transfer to a N-free solution for 3 d, and final exposure to 9.0 mM NH_4_^+^ (excess NH_4_^+^) for 6 h; **Cd toxicity and salt treatments**: For Cd- and salt-treatment, we cultivated 17-day-old Zhongshuang 11 seedlings in 10 µM CdCl_2_ and 200 mM NaCl for 12 h and 1 d, respectively. The seedlings in the control groups were cultivated in a normal solution for the appropriate times based on the aforementioned treatments. Transcriptome sequencing was performed using roots and shoots from control and stress-treated plants as described above [84–86].

## Conclusions

In this study, 1 798 *HM* genes were systematically identified in nine Brassicaceae species. Their chromosomal locations, protein/gene structure, and phylogenetic and syntenic relationships were characterized. The *BnaHMs* responding to A, salt, Cd, N, and K stress were investigated through differential expression analysis (GCNA and WGCNA). Taken together, *BnaPRMT11*, *BnaJMJ58*, *BnaSDG46*, *BnaHDA1*, *BnaSDG10*, and *BnaHDT10*, were identified as potential hub genes, especially *BnaSDG46* and *BnaHDT10*. Our results suggest that *BnaHMs* may be crucial for regulating stress adaptation in rapeseed. The candidate genes identified here should be validated in future studies.

## Electronic supplementary material

Below is the link to the electronic supplementary material.

Supplementary Material 1

Supplementary Material 2

Supplementary Material 3

Supplementary Material 4

Supplementary Material 5

Supplementary Material 6

Supplementary Material 7

Supplementary Material 8

Supplementary Material 9

Supplementary Material 10

Supplementary Material 11

## Data Availability

The raw transcriptome sequencing data were submitted to the National Centre for Biotechnology Information (NCBI) (http://www.ncbi.nlm.nih.gov/) under BioProject PRJNA340053, PRJNA718104, and PRJCA001323. The datasets used and/or analyzed in the current study are available from the corresponding author upon reasonable request.

## Abbreviations

*HMs*: Histone modification genes
NH_4_^+^: Ammonium
Cd: Cadmium
B: Boron
N: Nitrogen
K: Potassium
P: Phosphate
DEGs: Differentially expressed genes
WGCNA: Weighted gene co-expression network analysis
GCNA: Gene co-expression network analysis.
