# Supplementary material for: Genome-wide identification of Brassicaceae histone modification genes and their responses to abiotic stresses in allotetraploid rapeseed
Source: BMC Plant Biol. 2023 May 11;23:248. doi: 10.1186/s12870-023-04256-1 (PMC10173674; doi:10.1186/s12870-023-04256-1)
Supplement: Supplementary file 1 — Supplementary Material 1 [file 12870_2023_4256_MOESM1_ESM.docx]

**Supplemental Figure 1. Number of *HMs* among different Brassicaceae species.**


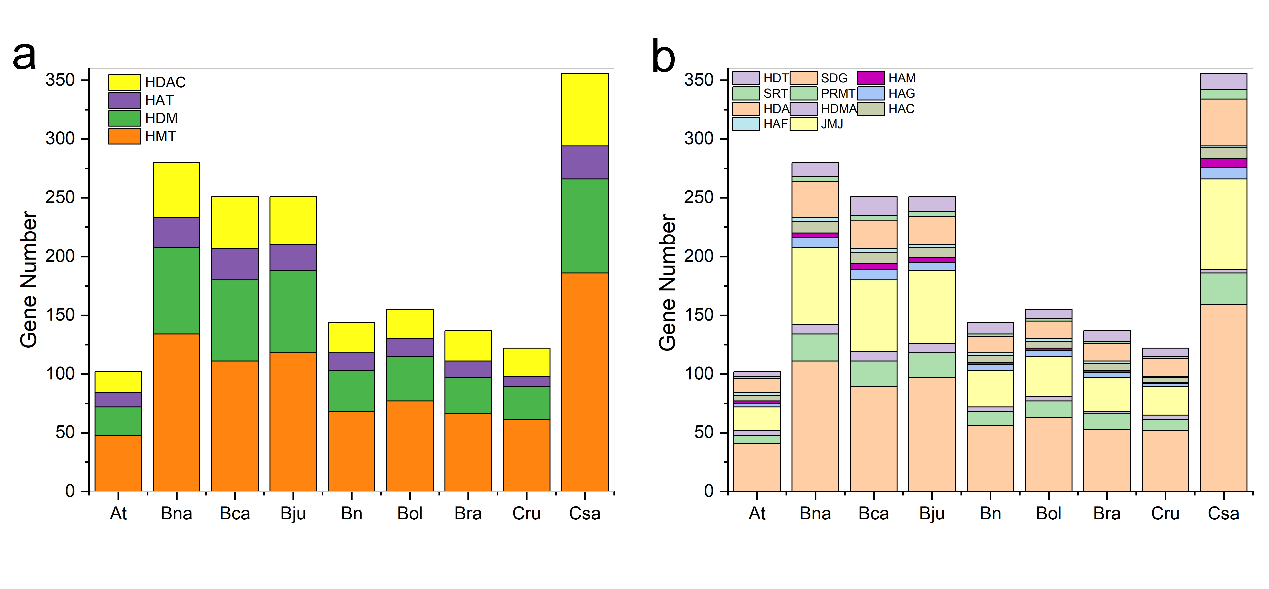


(**a**) Gene number of each *HM* family in *Arabidopsis thaliana* (At), *Brassica napus* (Bna), *Brassica carinata* (Bca), *Brassica juncea* (Bju), *Brassica nigra* (Bn), *Brassica oleracea* (Bol), *Brassica rapa* (Bra), *Capsella rubella* (Cru), *Camelina sativa* (Csa). (**b)** Gene number of each HM subfamily in *Arabidopsis thaliana* (At), *Brassica napus* (Bna), *Brassica carinata* (Bca), *Brassica juncea* (Bju), *Brassica nigra* (Bn), *Brassica oleracea* (Bol), *Brassica rapa* (Bra), *Capsella rubella* (Cru), *Camelina sativa* (Csa)
