# Supplementary material for: Genome-wide identification of Brassicaceae histone modification genes and their responses to abiotic stresses in allotetraploid rapeseed
Source: BMC Plant Biol. 2023 May 11;23:248. doi: 10.1186/s12870-023-04256-1 (PMC10173674; doi:10.1186/s12870-023-04256-1)

**Supplemental Figure 2. Chromosomal location of *HMs* in nine Brassicaceae species.**

**Fig. S2-1 The location of *Arabidopsis* *HMs* on chromosomes.**


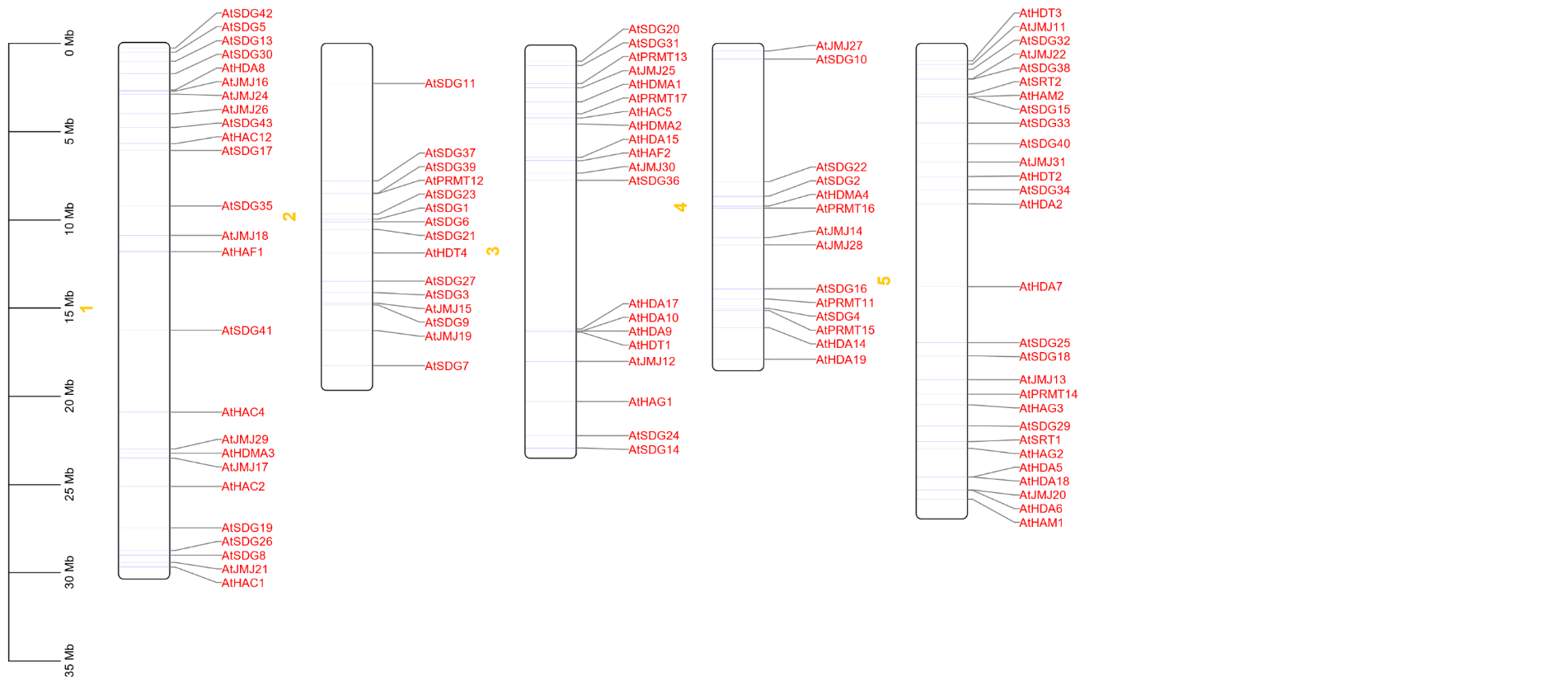


**Fig. S2-2 The location of *Brassica napus* *HMs* on chromosomes.**


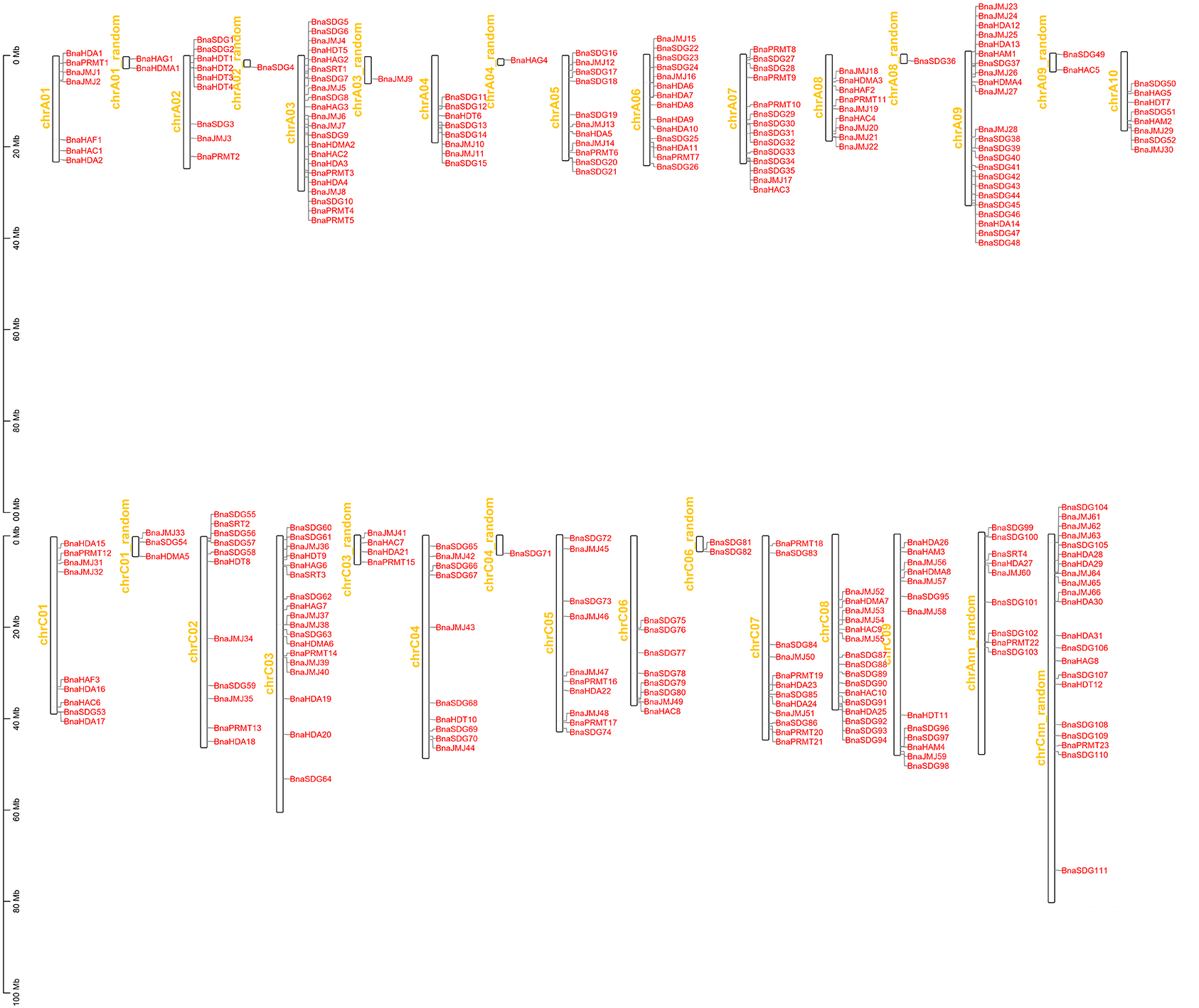


**Fig. S2-3 The location of *Brassica carinata* *HMs* on chromosomes.**


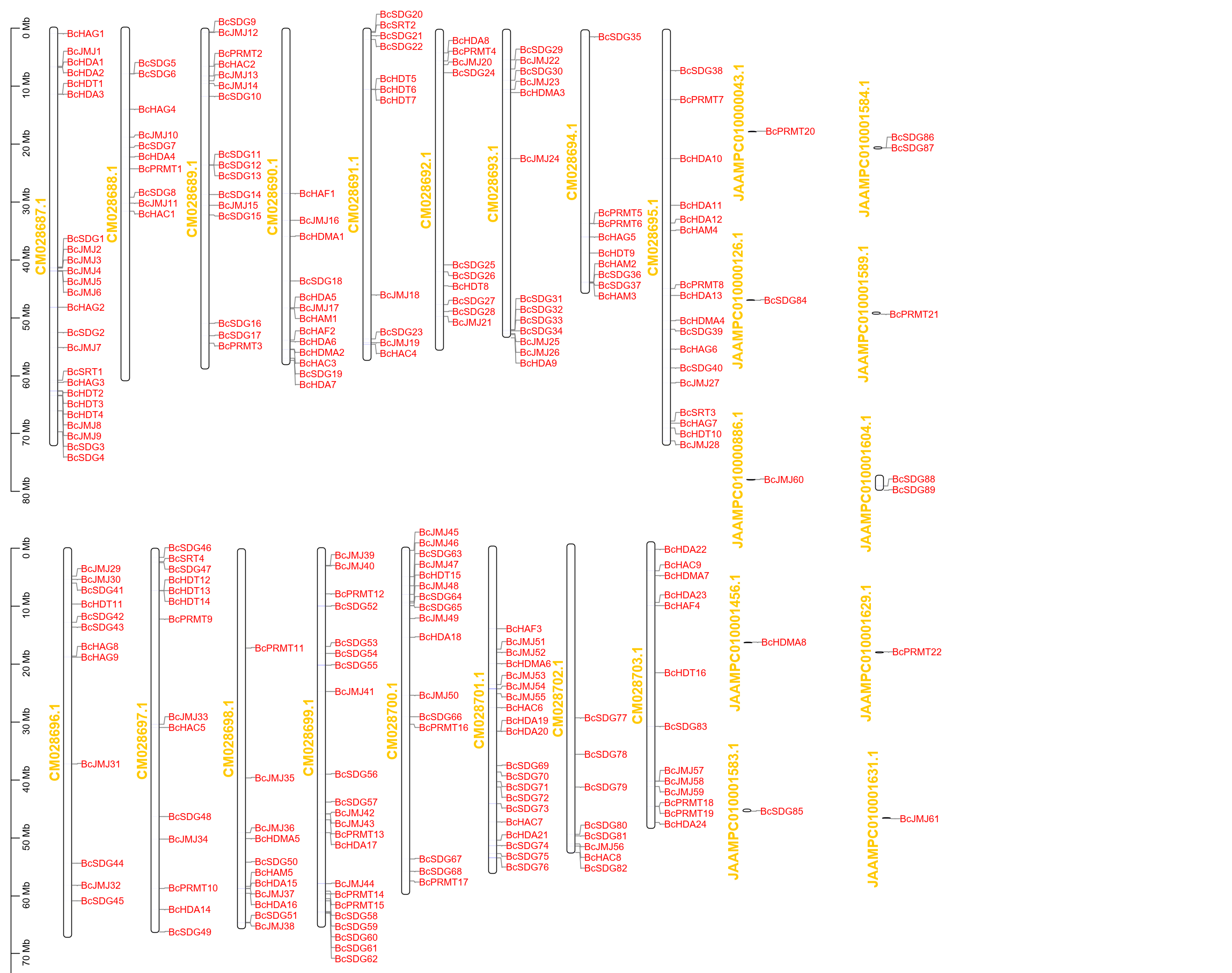


**Fig. S2-4 The location of *Brassica juncea* *HMs* on chromosomes.**


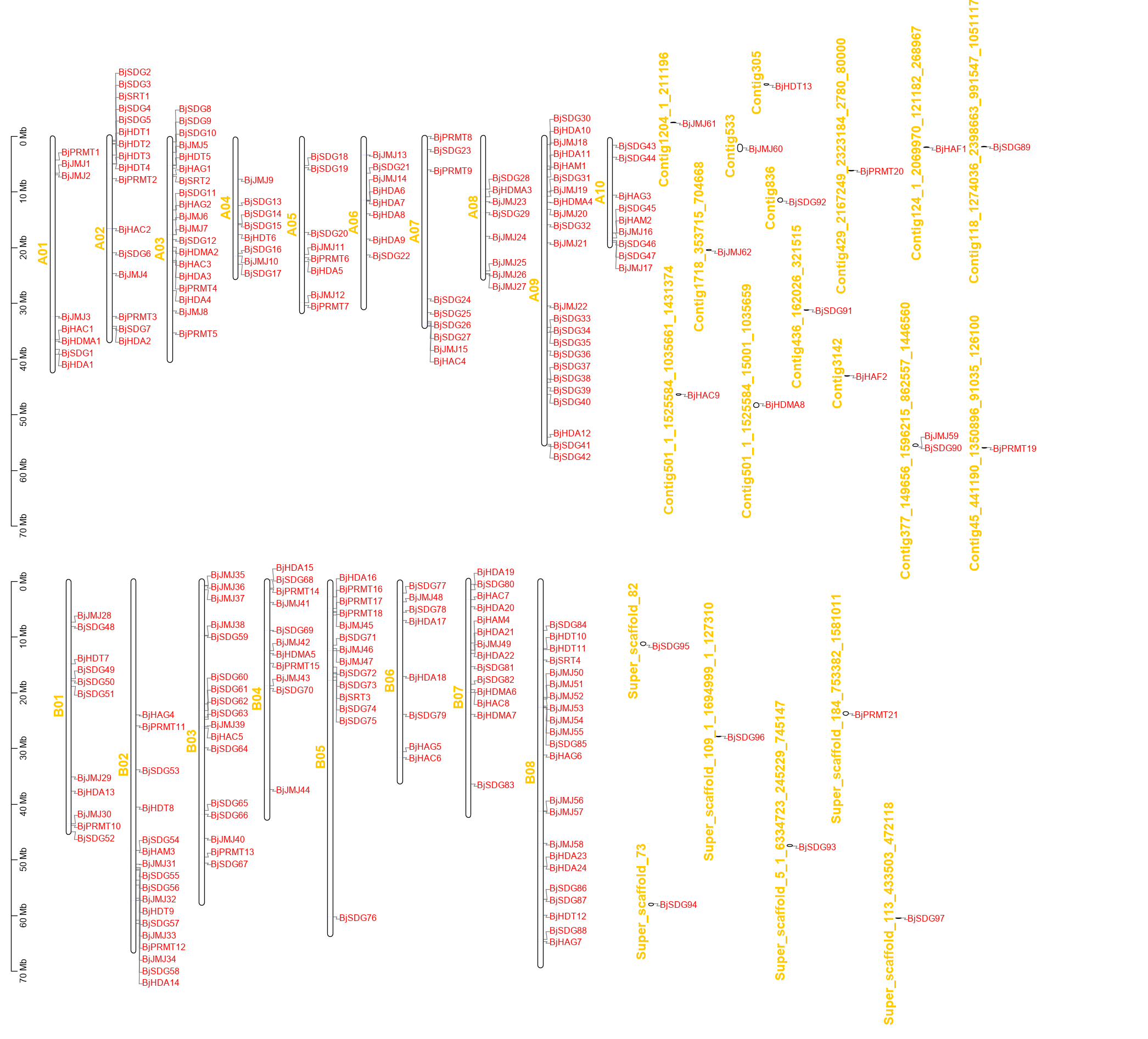


**Fig. S2-5 The location of *Brassica nigra* *HMs* on chromosomes.**


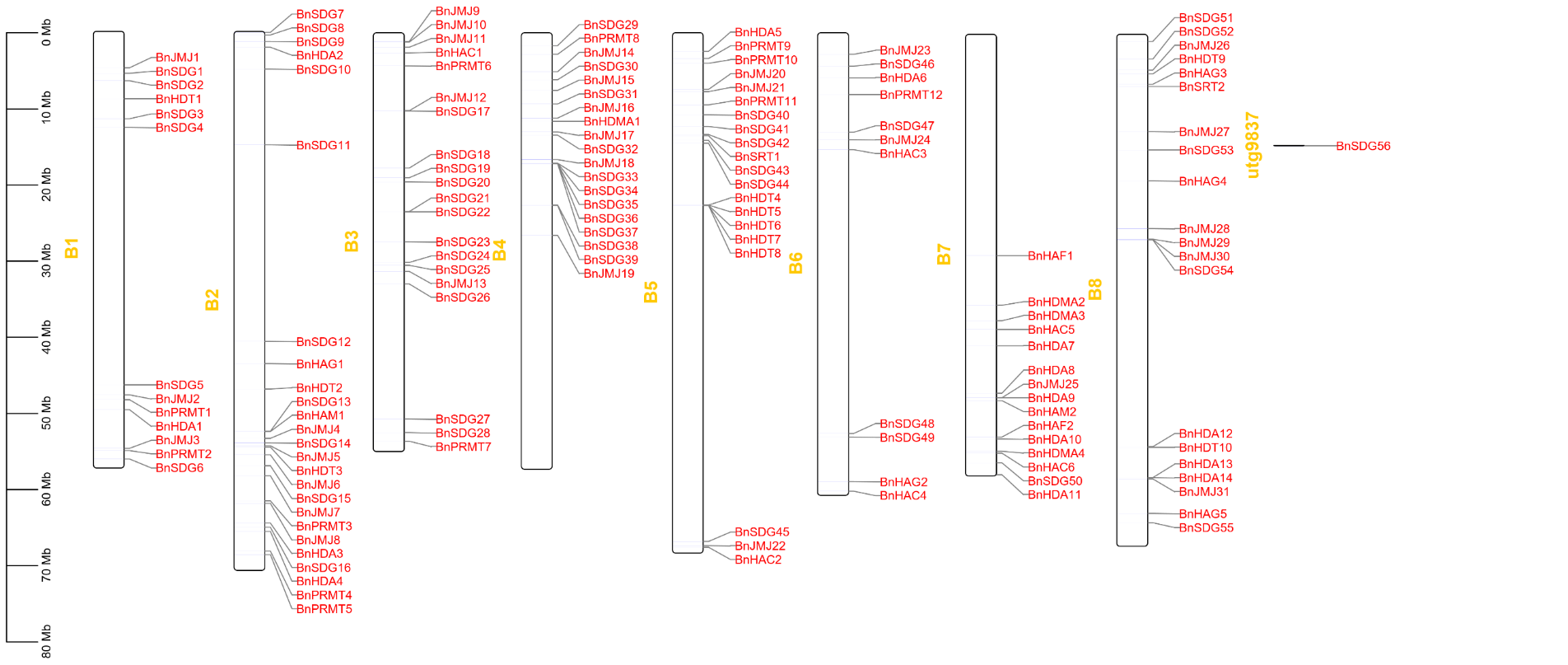


**Fig. S2-6 The location of *Brassica oleracea* *HMs* on chromosomes.**


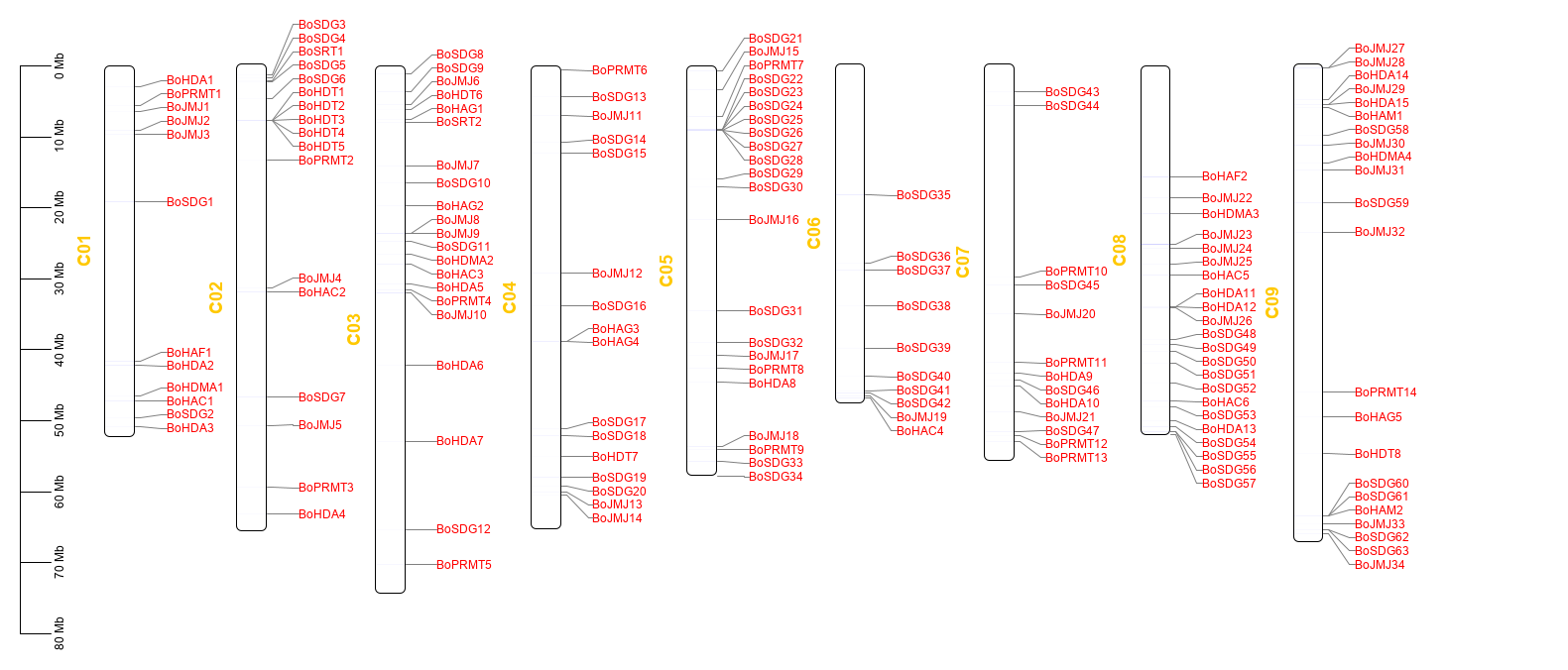


**Fig. S2-7 The location of *Brassica rapa* *HMs* on chromosomes.**


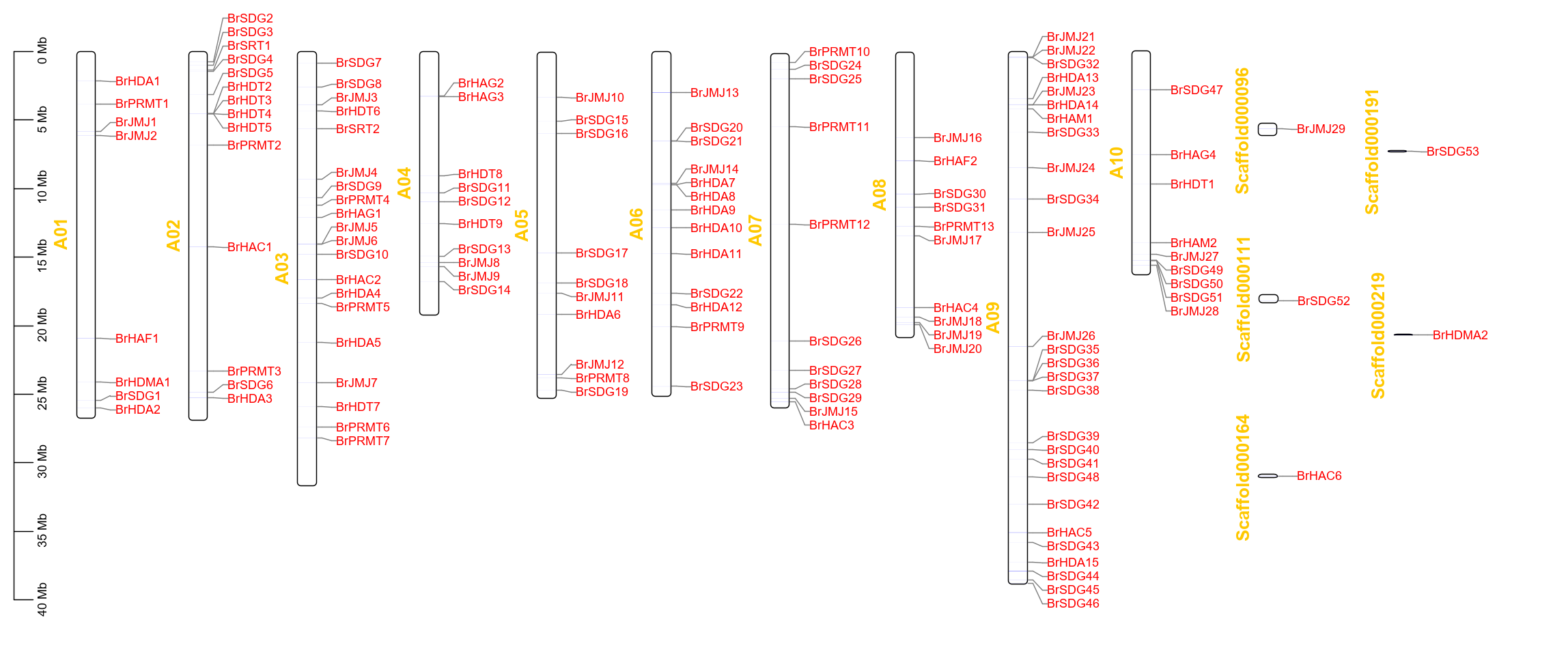


**Fig. S2-8 The location of *Camelina sativa* *HMs* on chromosomes.**


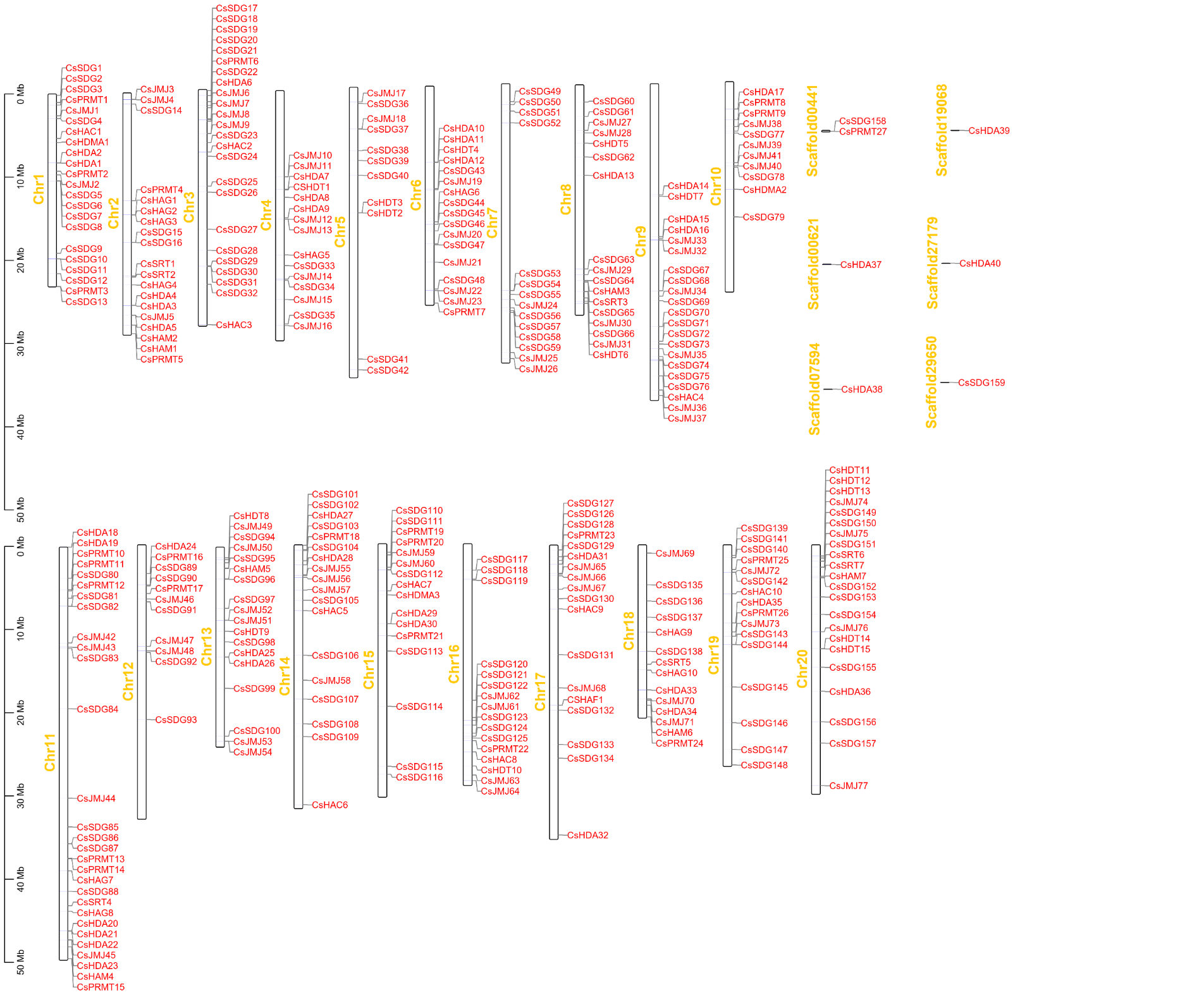


**Fig. S2-9 The location of *Capsella rubella* *HMs* on chromosomes.**


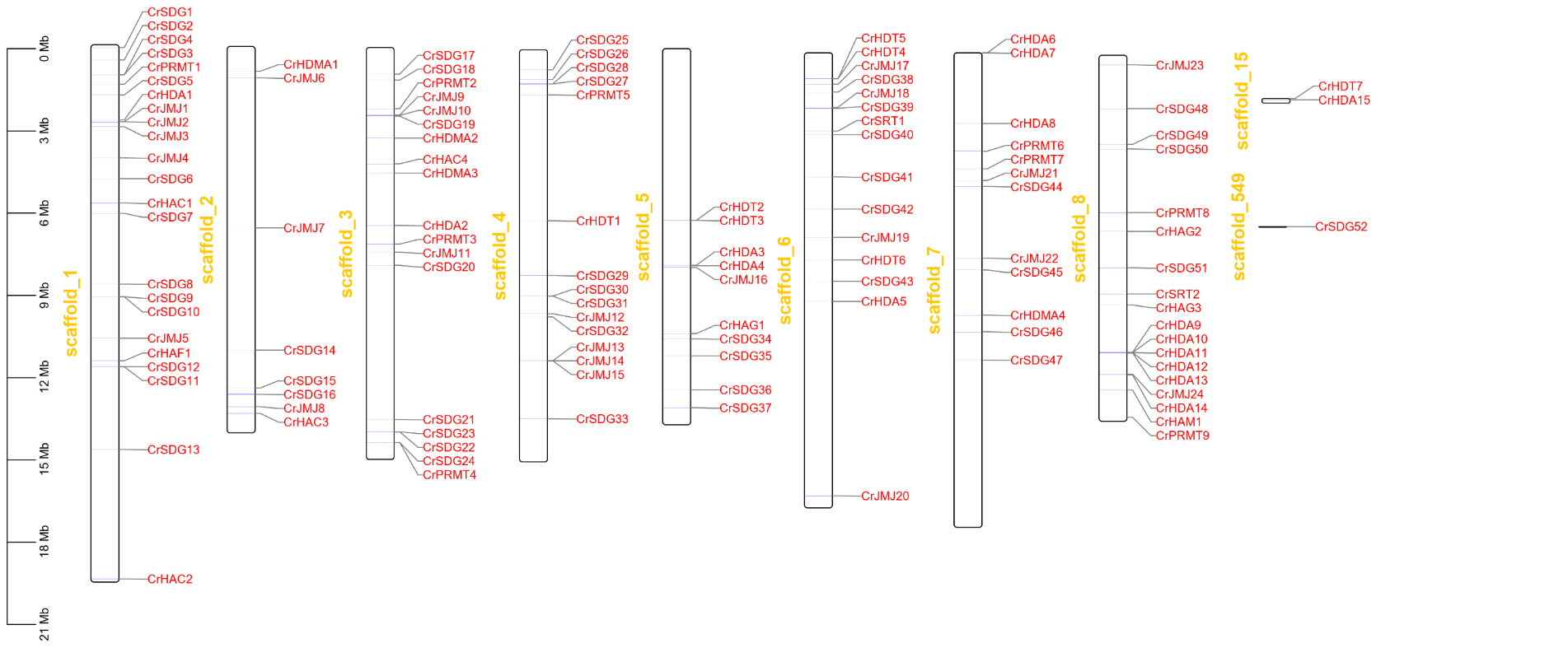

Supplement: Supplementary file 2 — Supplementary Material 2 [file 12870_2023_4256_MOESM2_ESM.docx]
