# Supplementary material for: Genome-wide identification of Brassicaceae histone modification genes and their responses to abiotic stresses in allotetraploid rapeseed
Source: BMC Plant Biol. 2023 May 11;23:248. doi: 10.1186/s12870-023-04256-1 (PMC10173674; doi:10.1186/s12870-023-04256-1)

**Supplemental Figure 3. Phylogenetic analysis of *HM* genes.**

**Fig. S3-1 Phylogenetic analysis of *HAT* genes.**


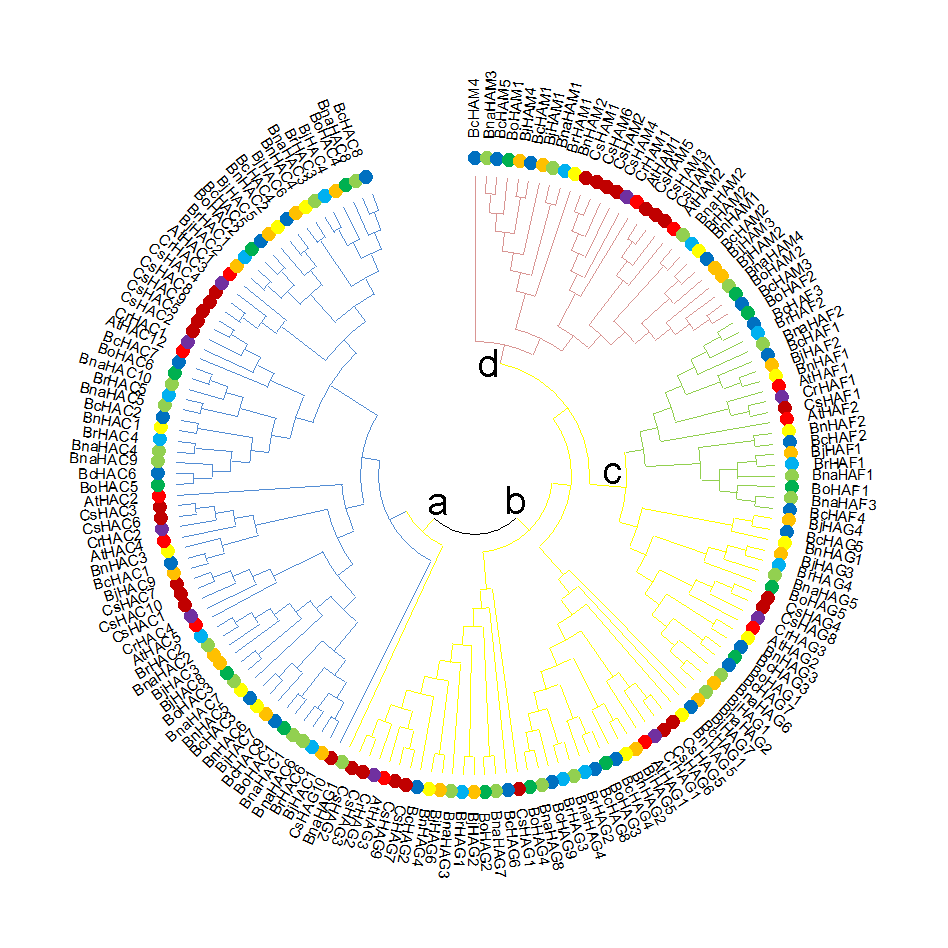


**Fig. S3-2 Phylogenetic analysis of *HDAC* genes.**


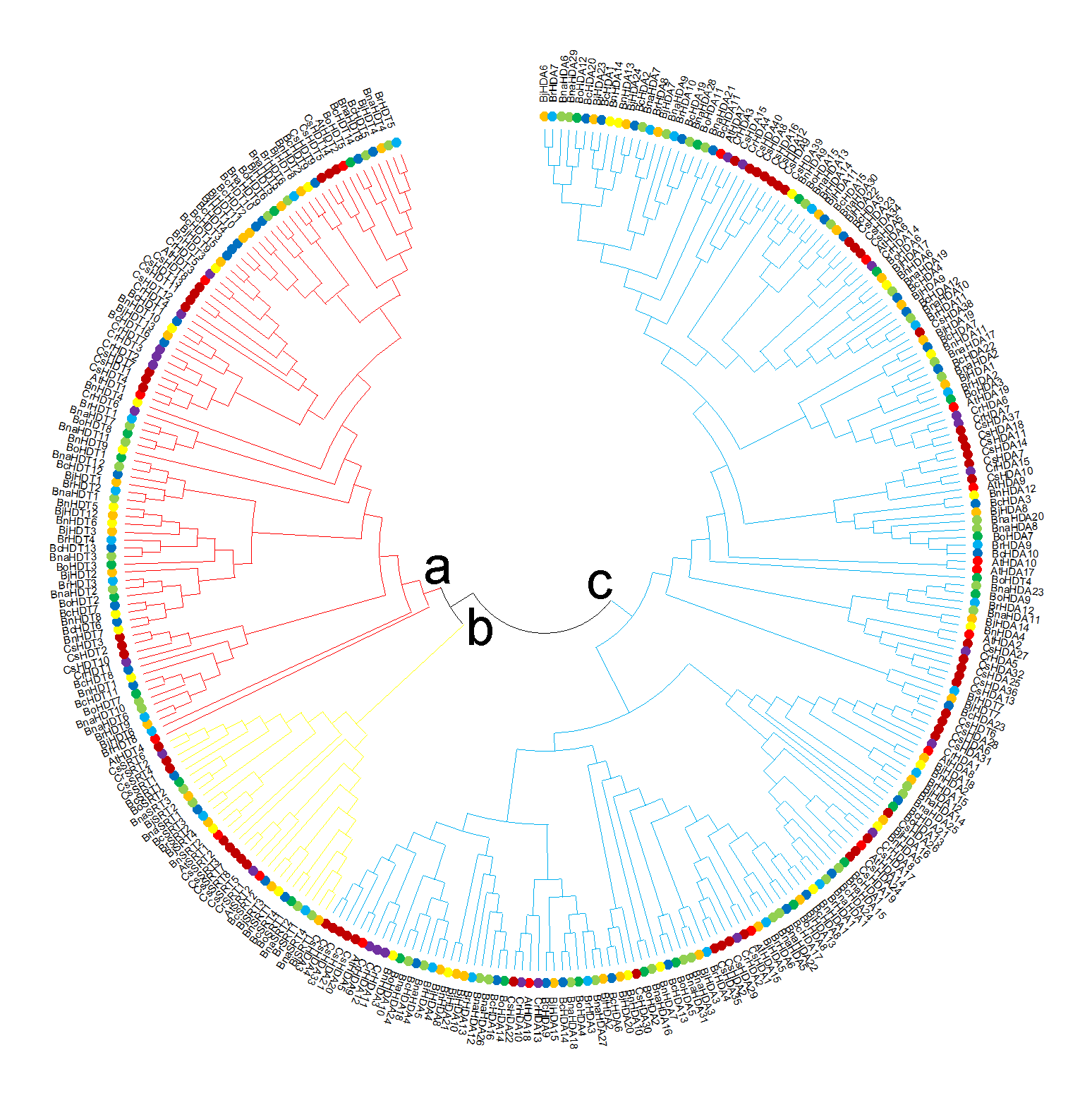


**Fig. S3-3 Phylogenetic analysis of HDM genes.**


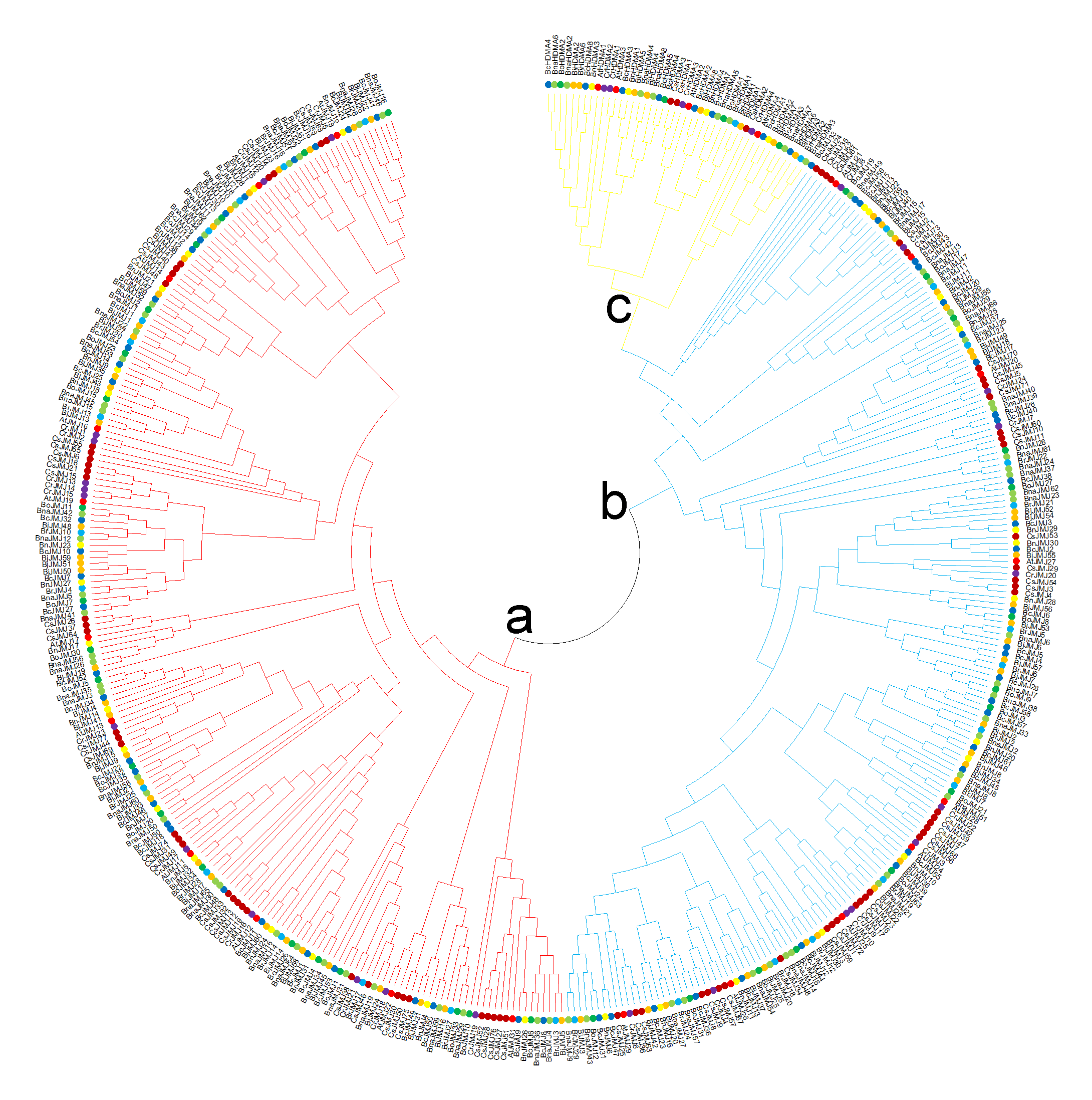


**Fig. S3-4 Phylogenetic analysis of *HMT* genes.**


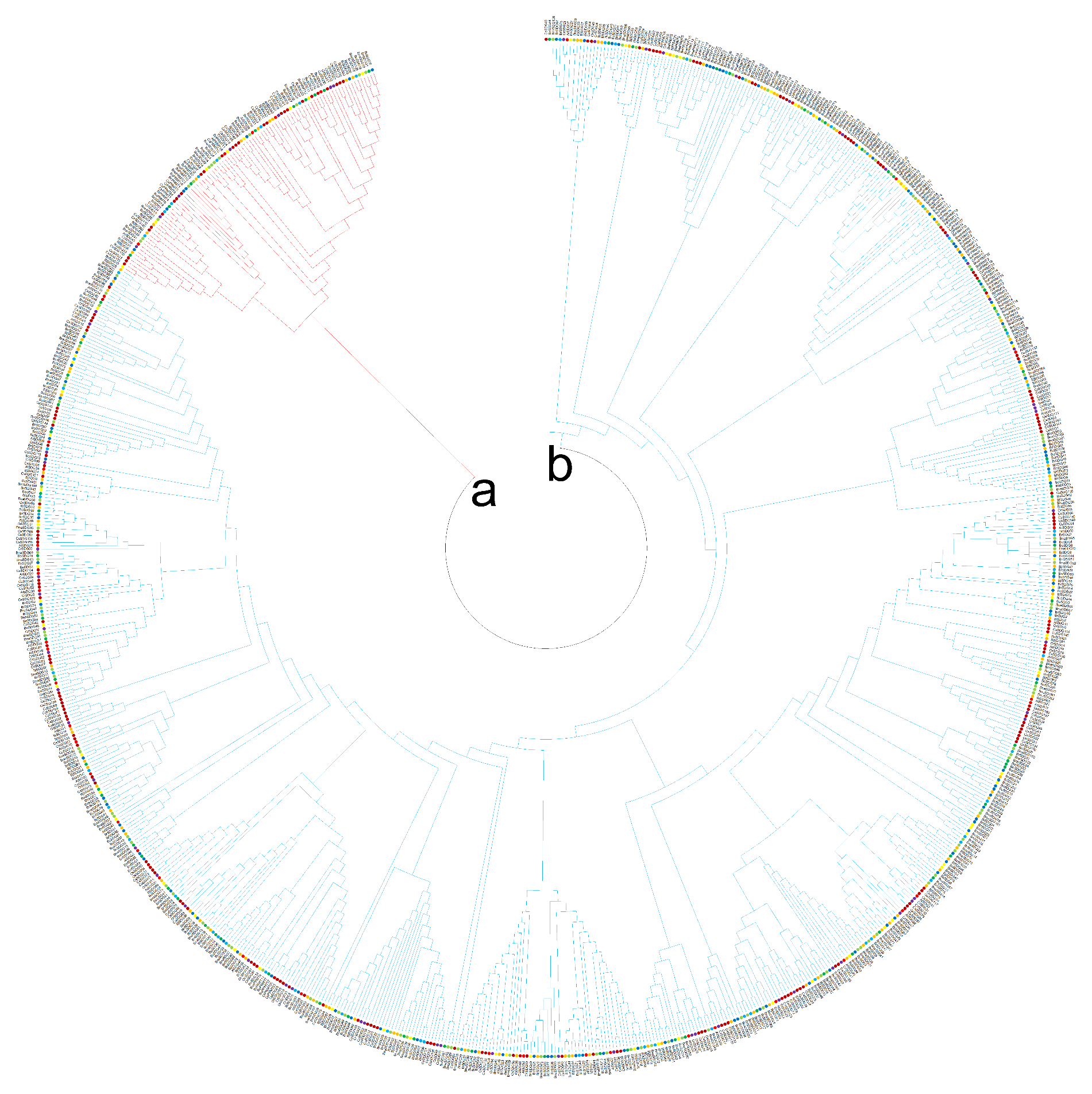

Supplement: Supplementary file 3 — Supplementary Material 3 [file 12870_2023_4256_MOESM3_ESM.docx]
