# Supplementary material for: Genome-wide identification of Brassicaceae histone modification genes and their responses to abiotic stresses in allotetraploid rapeseed
Source: BMC Plant Biol. 2023 May 11;23:248. doi: 10.1186/s12870-023-04256-1 (PMC10173674; doi:10.1186/s12870-023-04256-1)

**Supplemental Figure 4. Conserved domains, gene structure analysis of *HM* genes in nine Brassicacaea species**

**Fig. S4-1 Conserved domains and gene structure analysis of *HM* genes in *Arabidopsis thaliana***


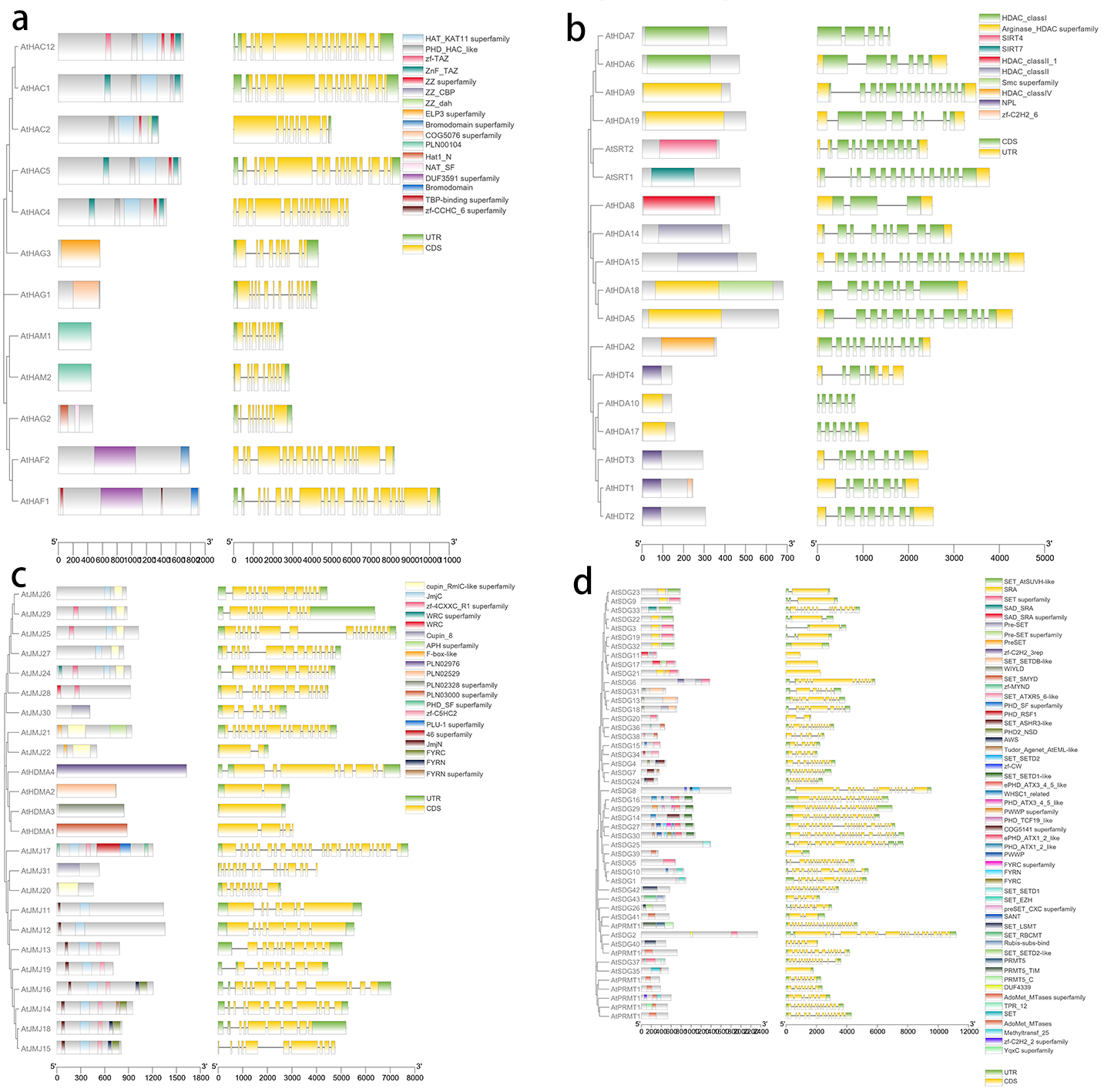


**Fig. S4-2 Conserved domains and gene structure analysis of *HM* genes in *Brassica***

***napus***
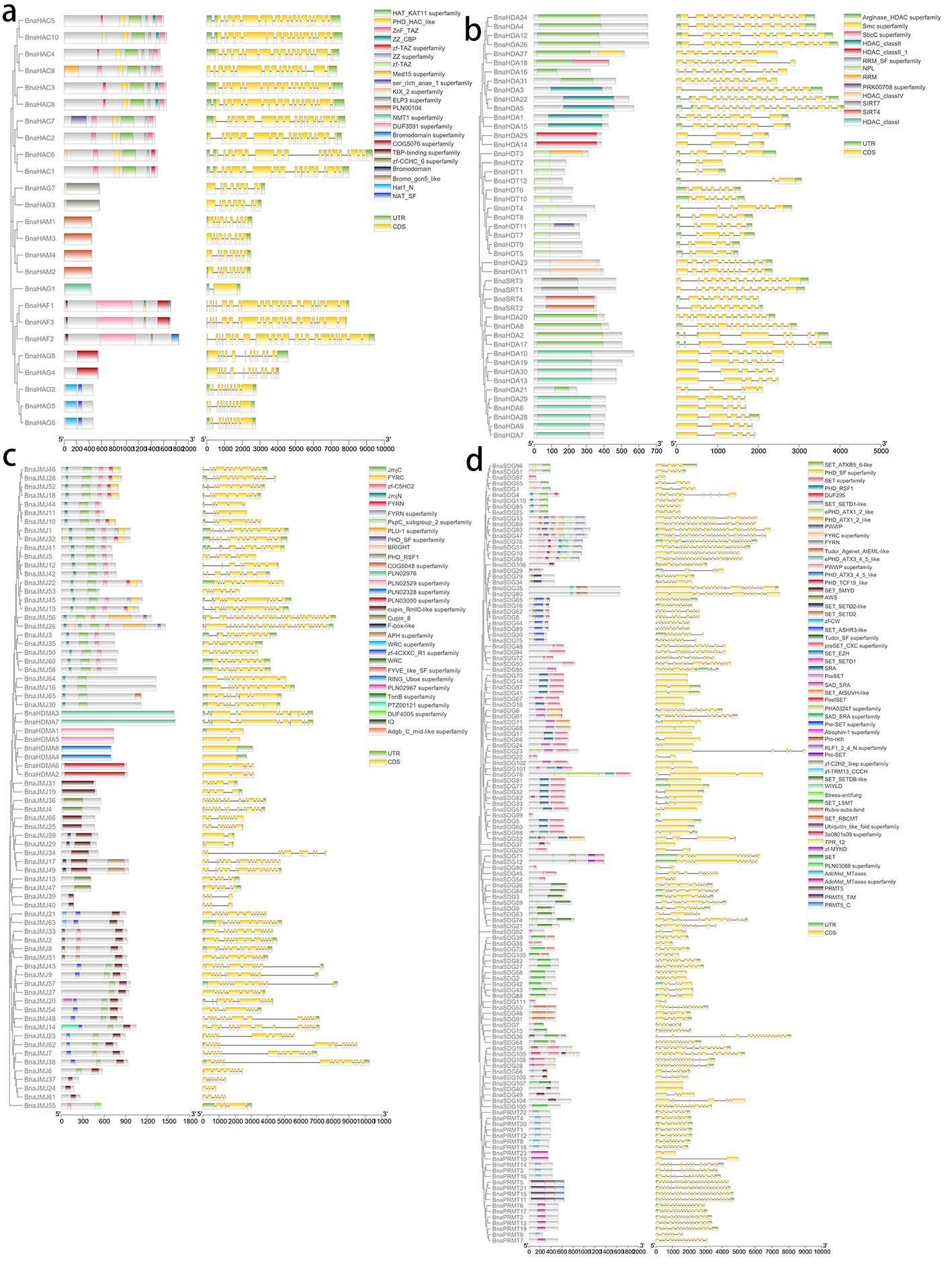


**Fig. S4-3 Conserved domains and gene structure analysis of *HM* genes in *Brassica oleracea***


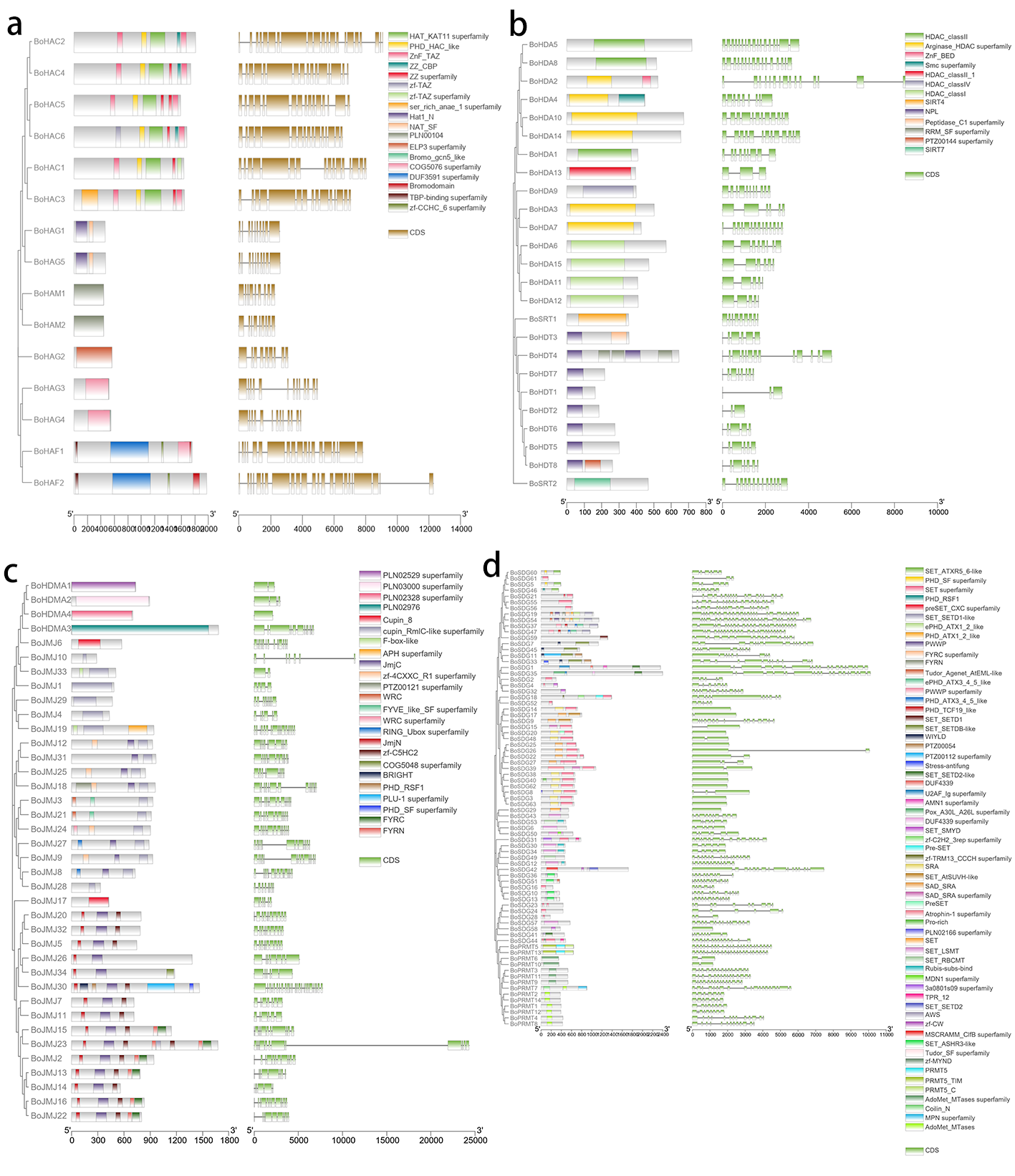


**Fig. S4-4 Conserved domains and gene structure analysis of *HM* genes in *Brassica rapa***


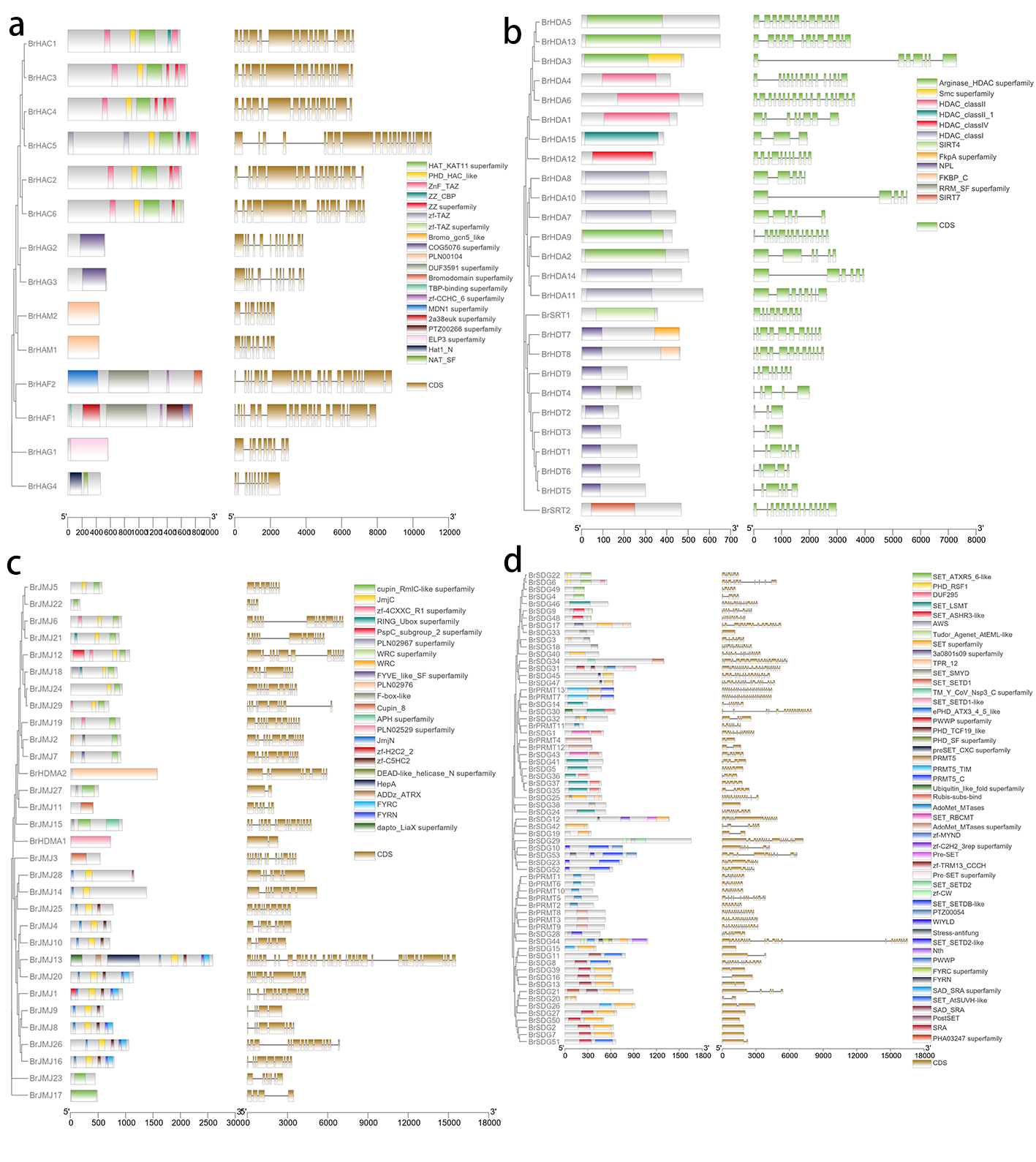


**Fig. S4-5 Conserved domains and gene structure analysis of *HM* genes in *Capsella rubella.***


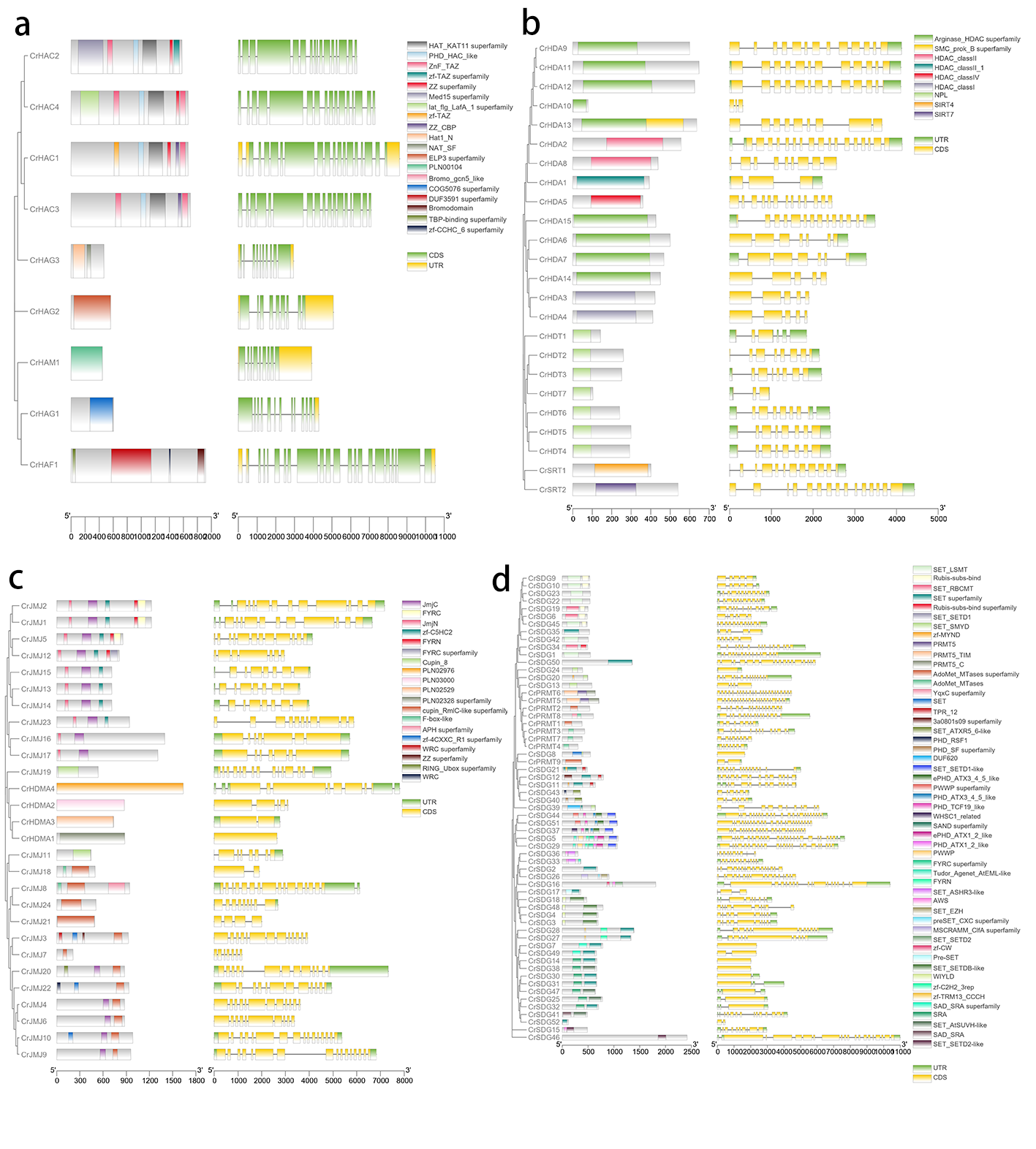


**Fig. S4-6** **Conserved domains and gene structure analysis of *HM* genes in *Camelina sativa.***


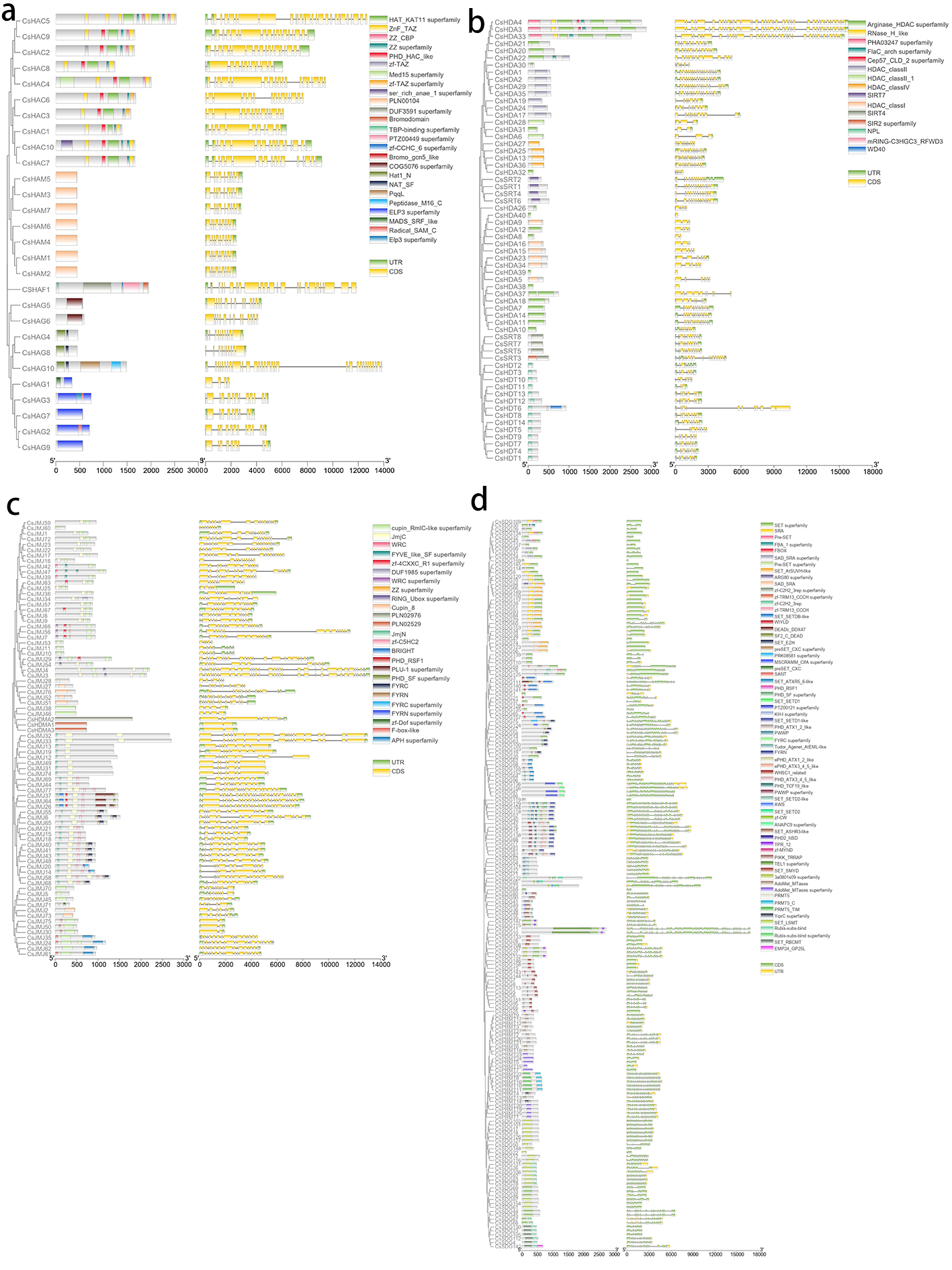


**Fig. S4-7** **Conserved domains and gene structure analysis of *HM* genes in *Brassica nigra.***


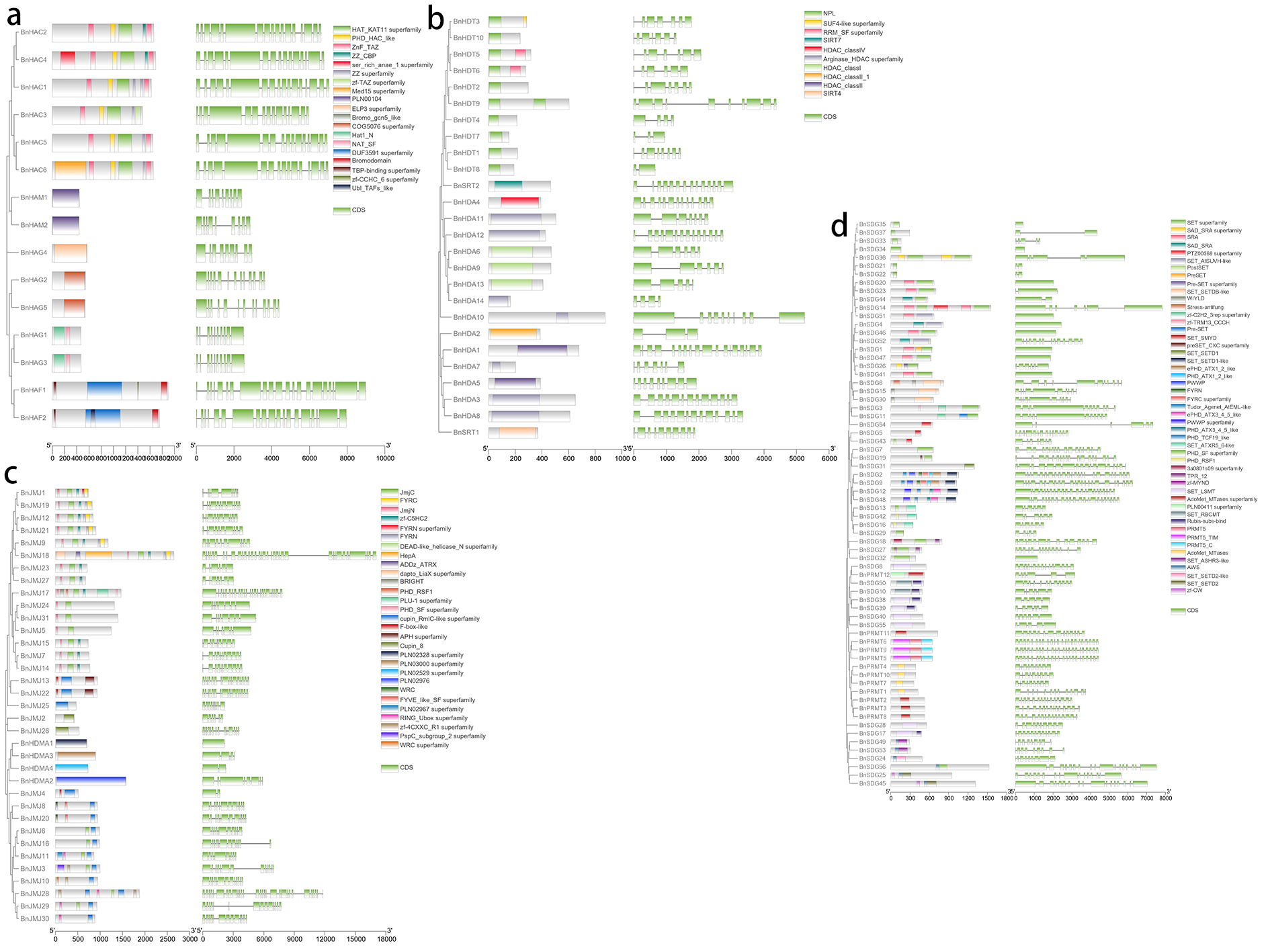


**Fig. S4-8** **Conserved domains and gene structure analysis of *HM* genes in *Brassica juncea.***


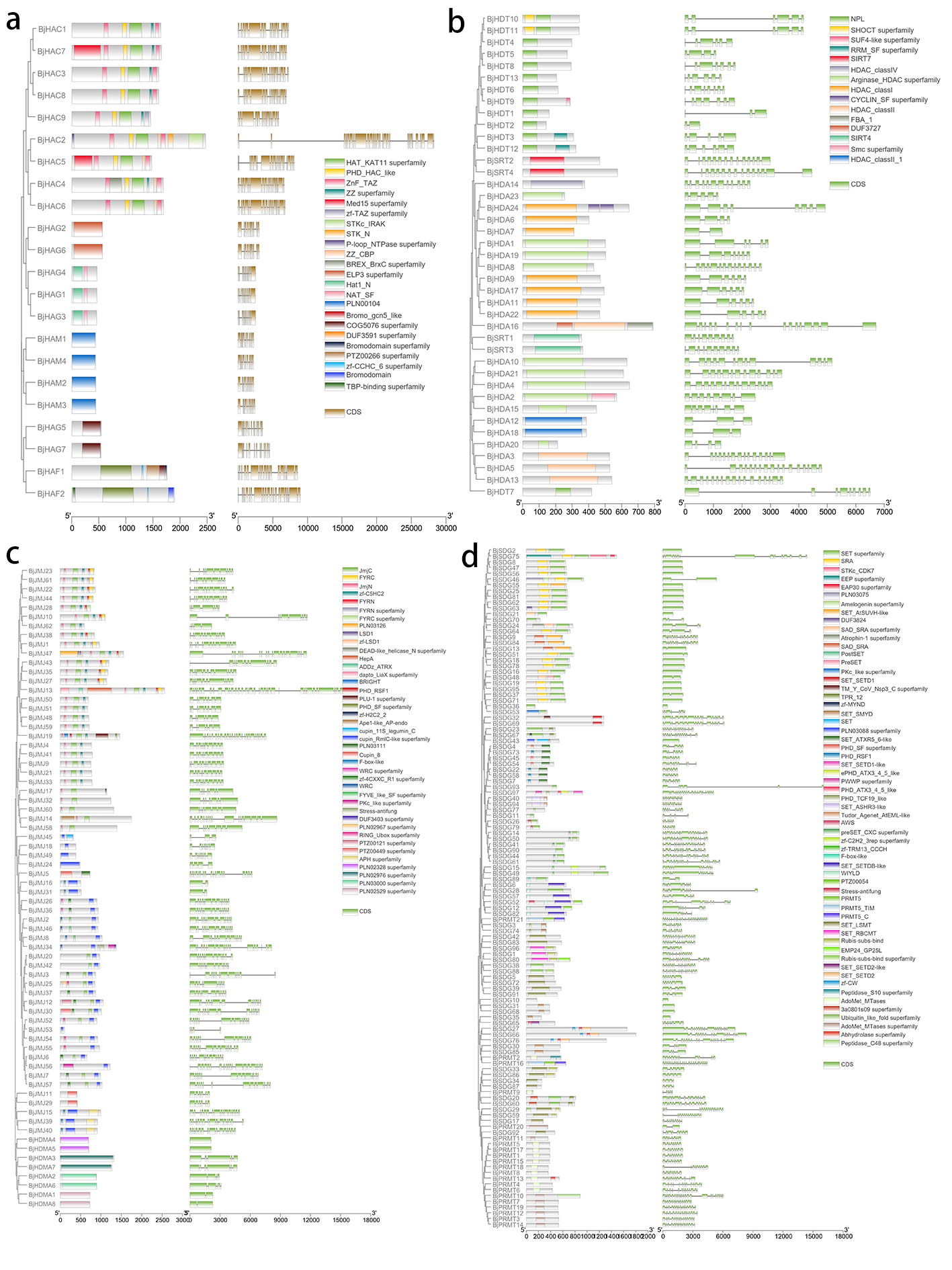


**Fig. S4-9** **Conserved domains and gene structure analysis of *HM* genes in *Brassica carinata.***


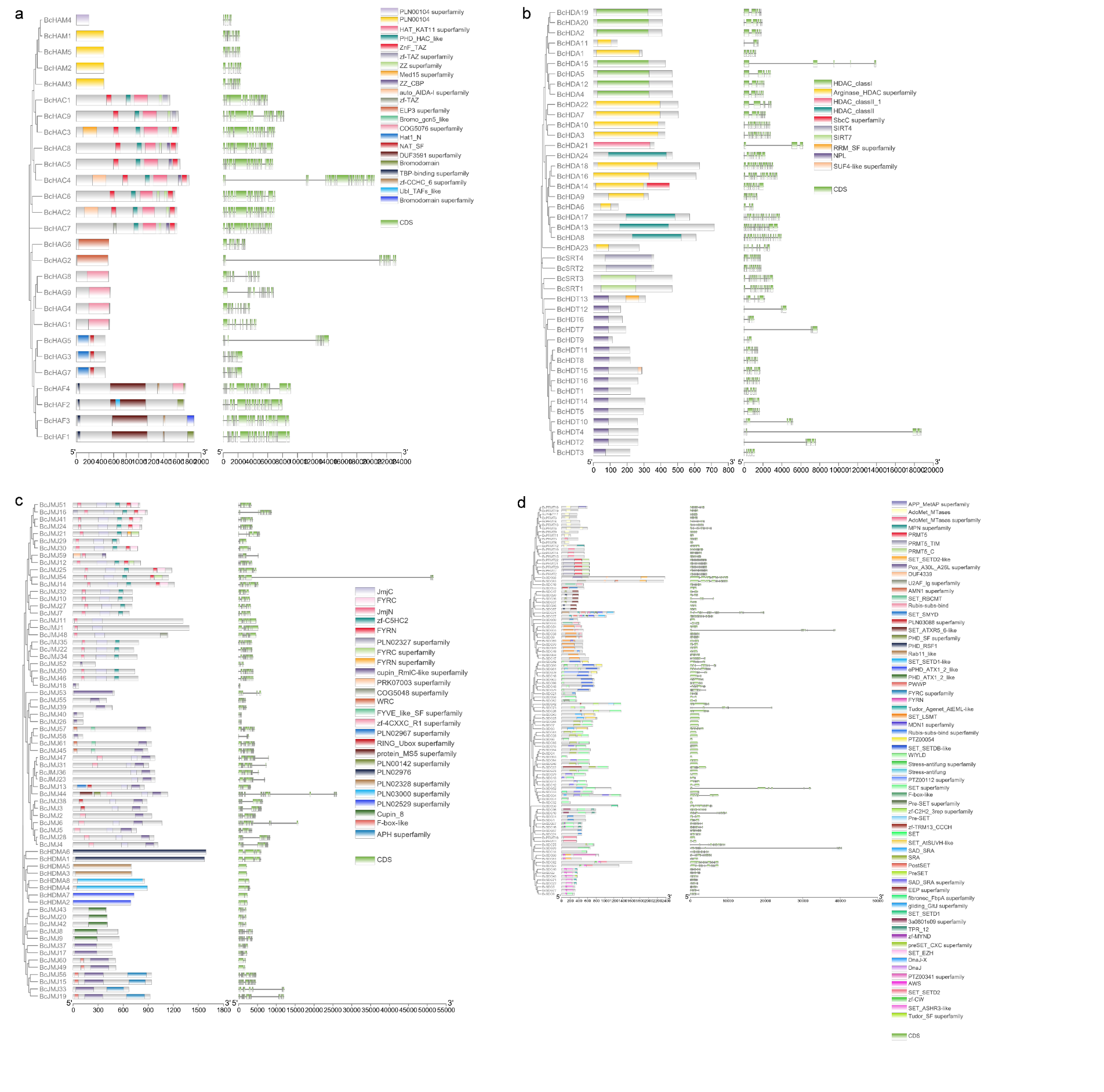

Supplement: Supplementary file 4 — Supplementary Material 4 [file 12870_2023_4256_MOESM4_ESM.docx]
