# Supplementary material for: Genome-wide identification of Brassicaceae histone modification genes and their responses to abiotic stresses in allotetraploid rapeseed
Source: BMC Plant Biol. 2023 May 11;23:248. doi: 10.1186/s12870-023-04256-1 (PMC10173674; doi:10.1186/s12870-023-04256-1)

**Supplemental Figure 5. Synteny analysis of *HM* genes in nine Brassicacaea species**

**Fig. S5-1 Synteny of *HM* genes in *Arabidopsis thaliana***


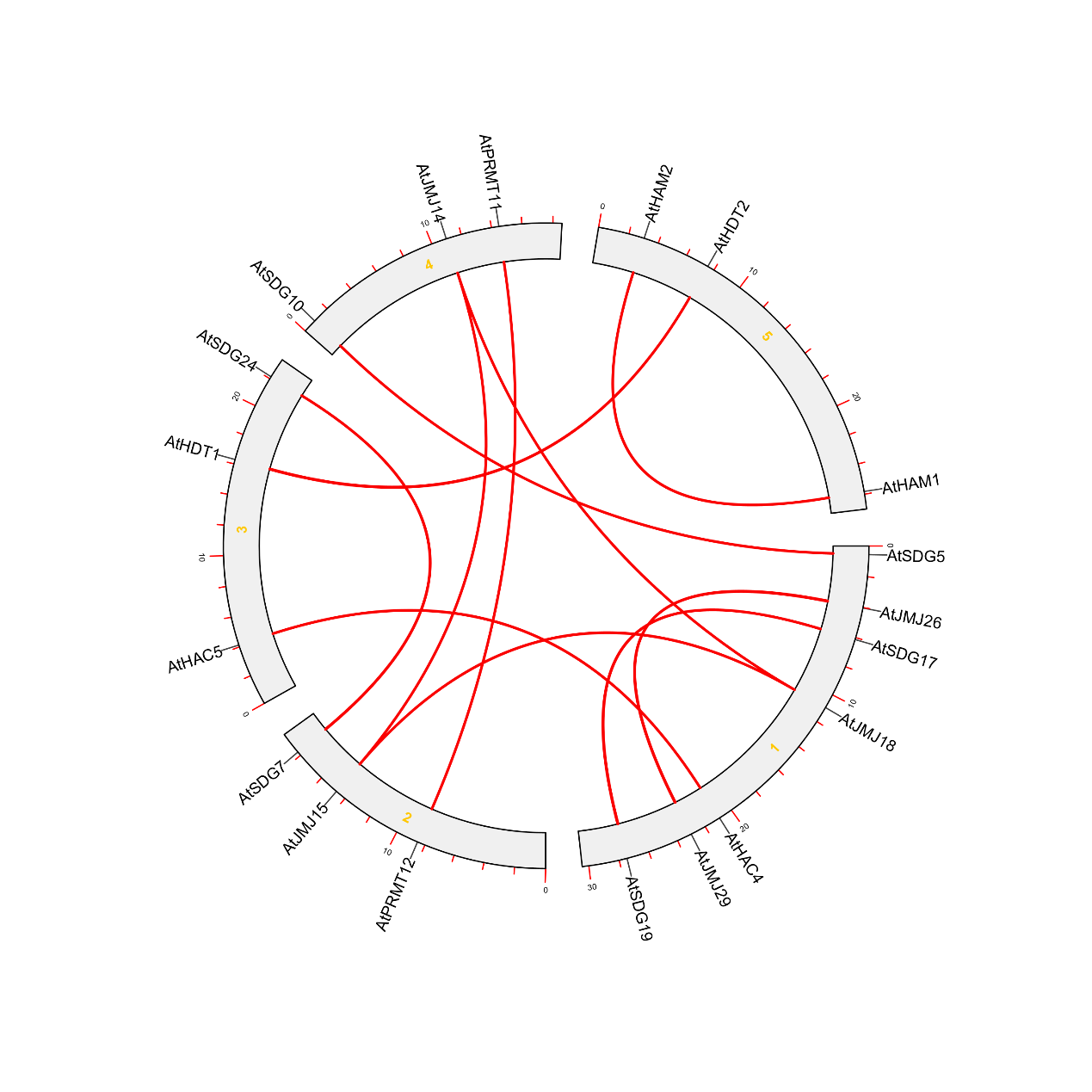


**Fig. S5-2 Synteny of *HM* genes in *Brassica napus***


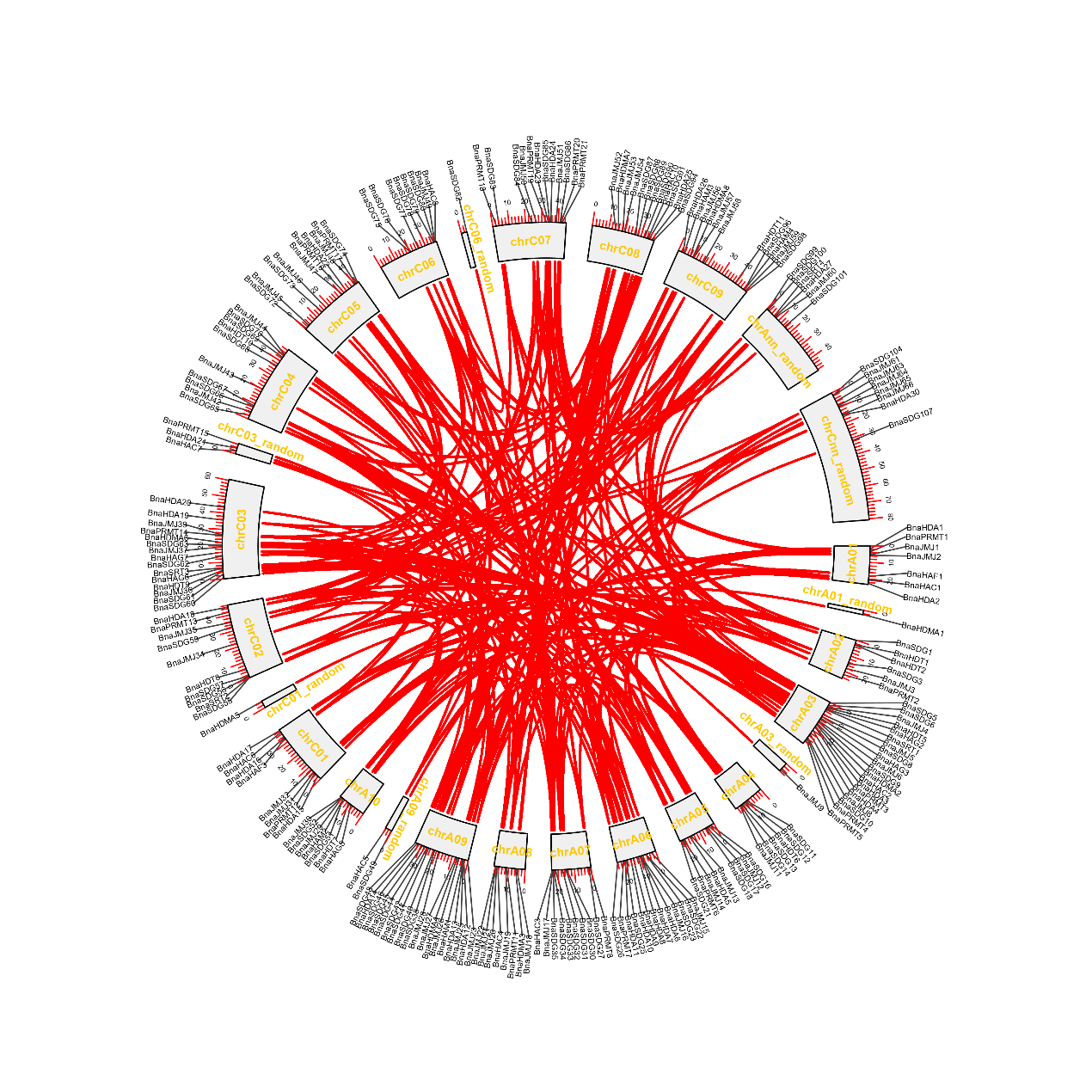


**Fig. S5-3 Synteny of *HM* genes in *Brassica carinata***


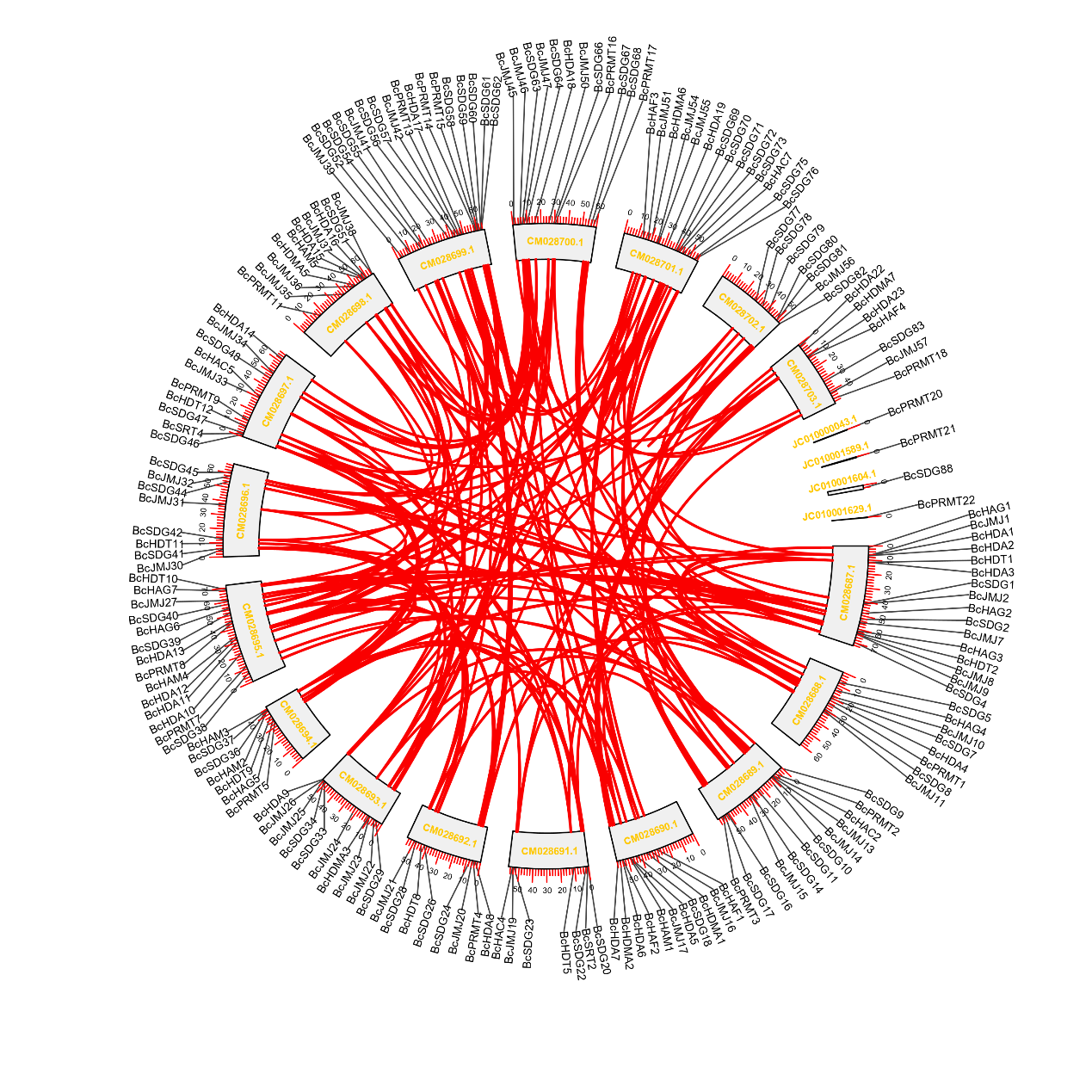


**Fig. S5-4 Synteny of *HM* genes in *Brassica juncea***


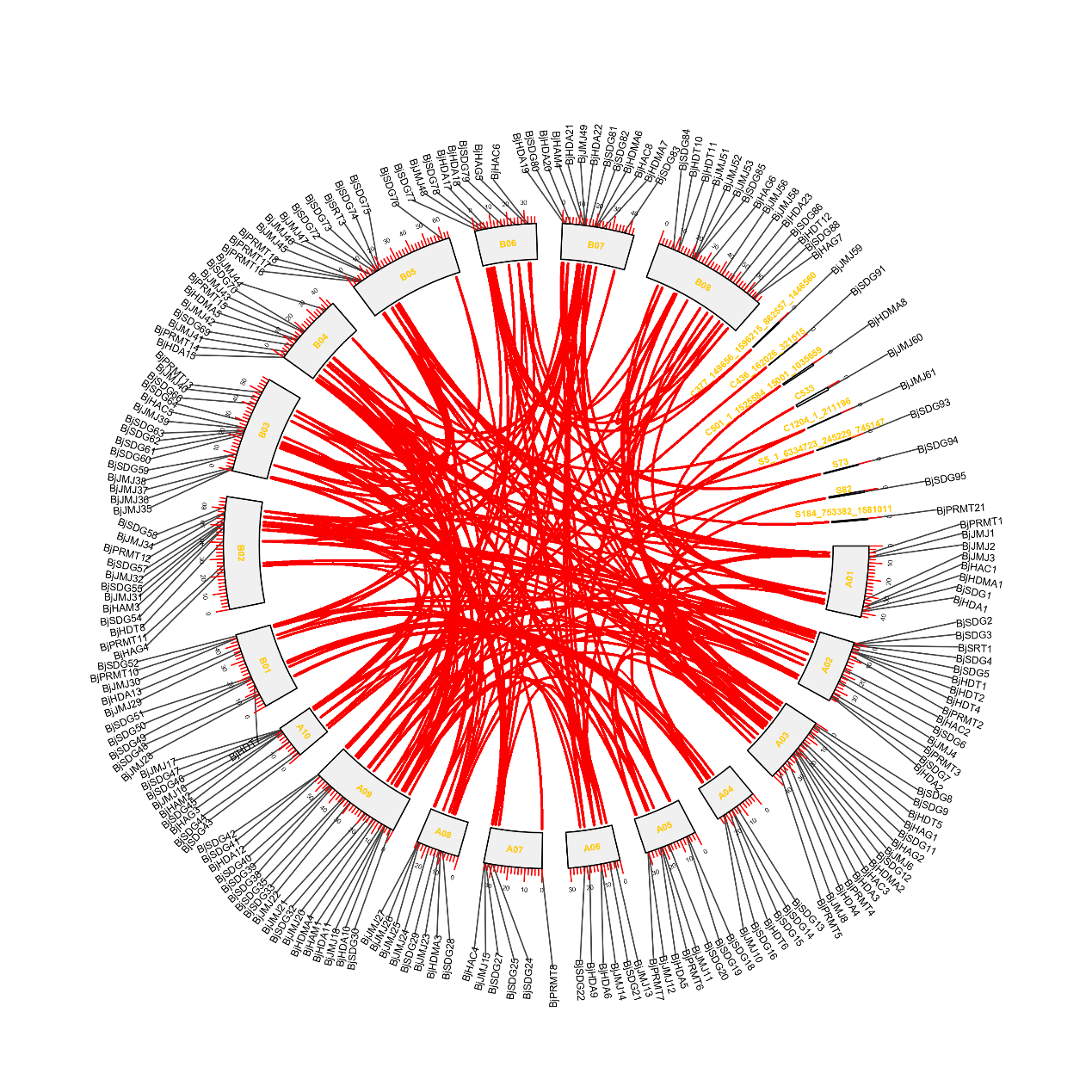


**Fig. S5-5 Synteny of *HM* genes in *Brassica nigra***


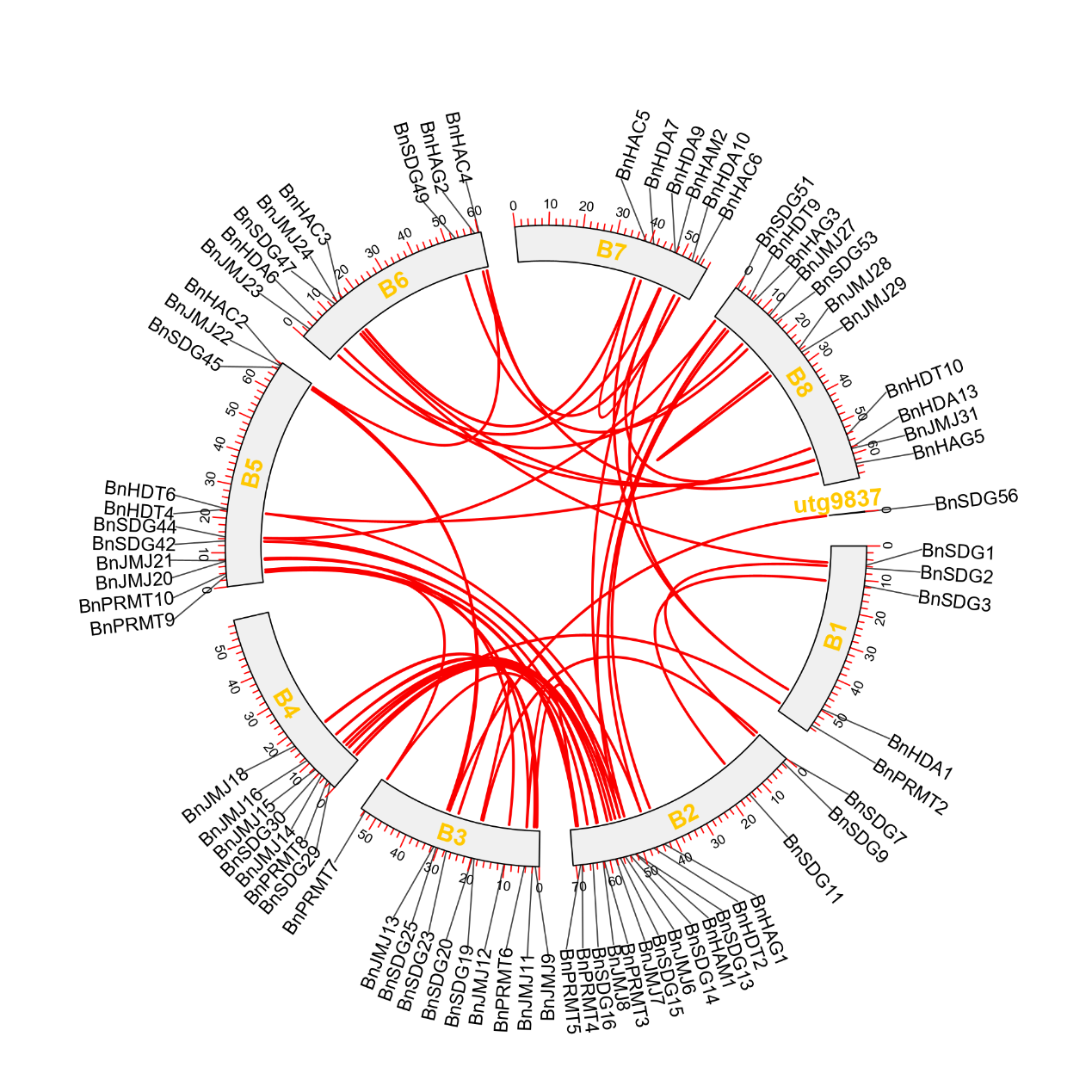


**Fig. S5-6 Synteny of *HM* genes in *Brassica oleracea***


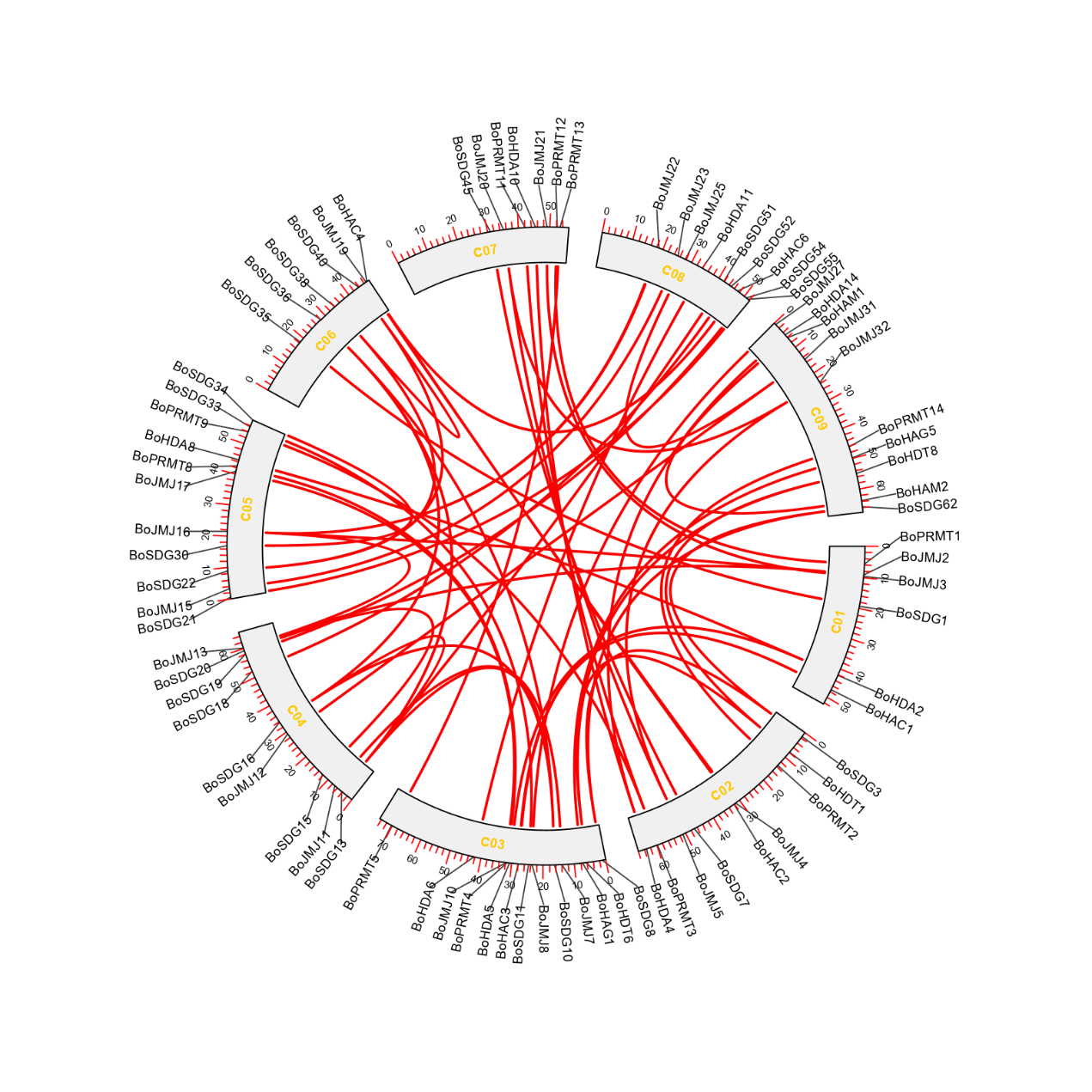


**Fig. S5-7 Synteny of *HM* genes in *Brassica rapa***


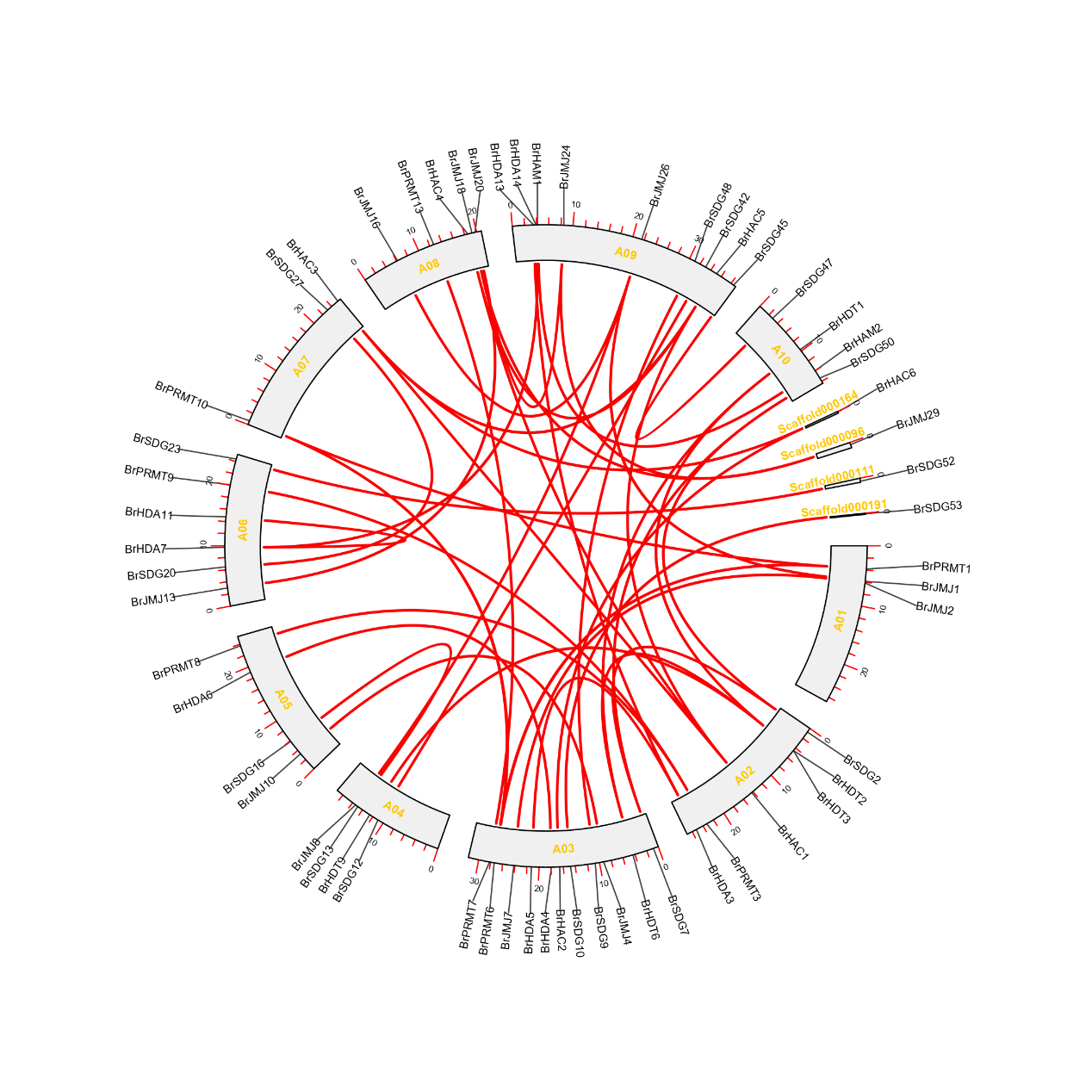


**Fig. S5-8 Synteny of *HM* genes in *Capsella rubella***


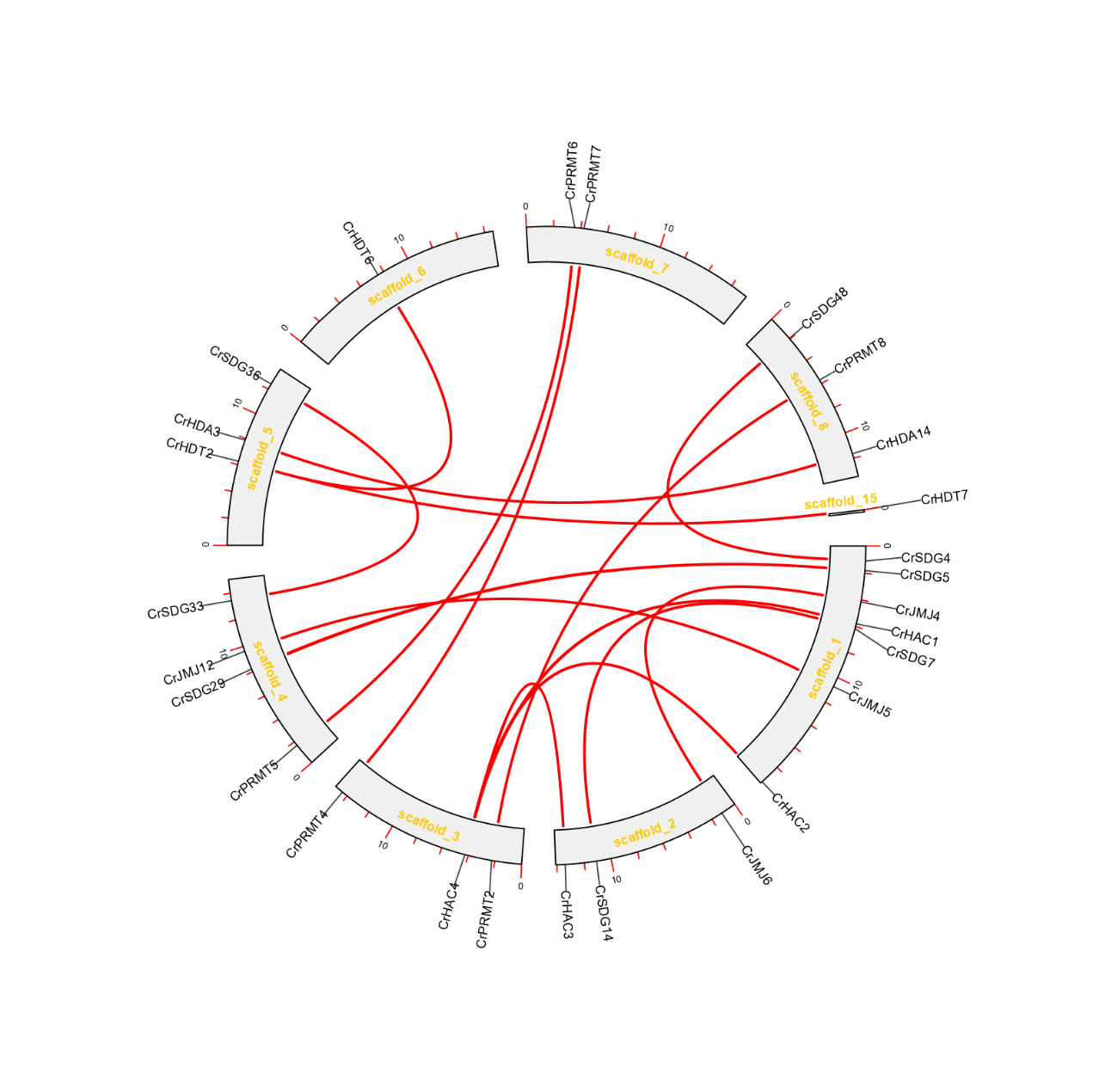


**Fig. S5-9 Synteny of *HM* genes in *Camelina sativa***


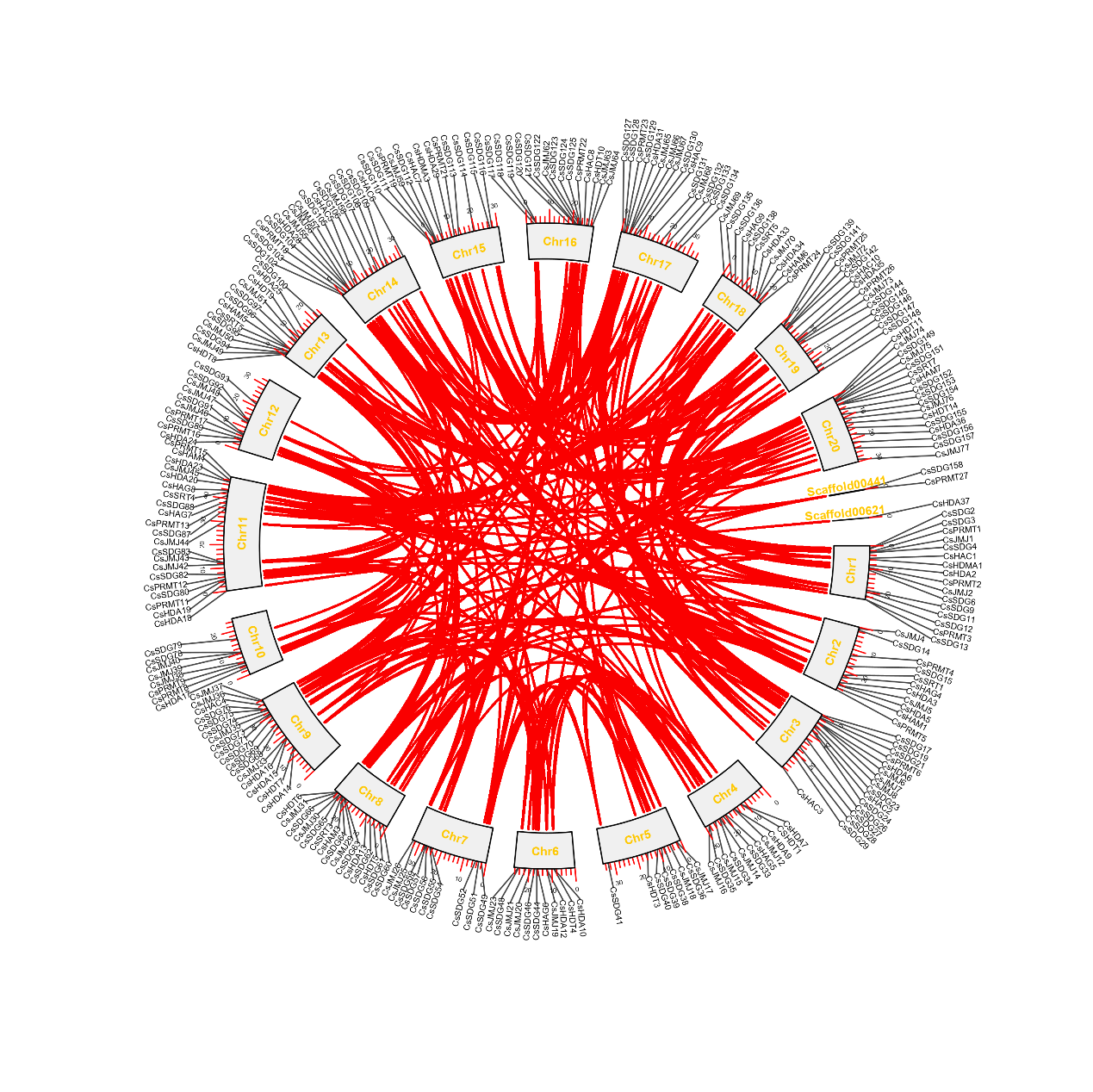

Supplement: Supplementary file 5 — Supplementary Material 5 [file 12870_2023_4256_MOESM5_ESM.docx]
