# Supplementary material for: Genome-wide identification of Brassicaceae histone modification genes and their responses to abiotic stresses in allotetraploid rapeseed
Source: BMC Plant Biol. 2023 May 11;23:248. doi: 10.1186/s12870-023-04256-1 (PMC10173674; doi:10.1186/s12870-023-04256-1)

**Supplemental Figure 6. Synteny analysis of *HM* genes between *Arabidopsis* and other Brassicacaea species**

**Fig. S6-1 Synteny of *HM* genes between *Arabidopsis* and *Brassica carinata.***


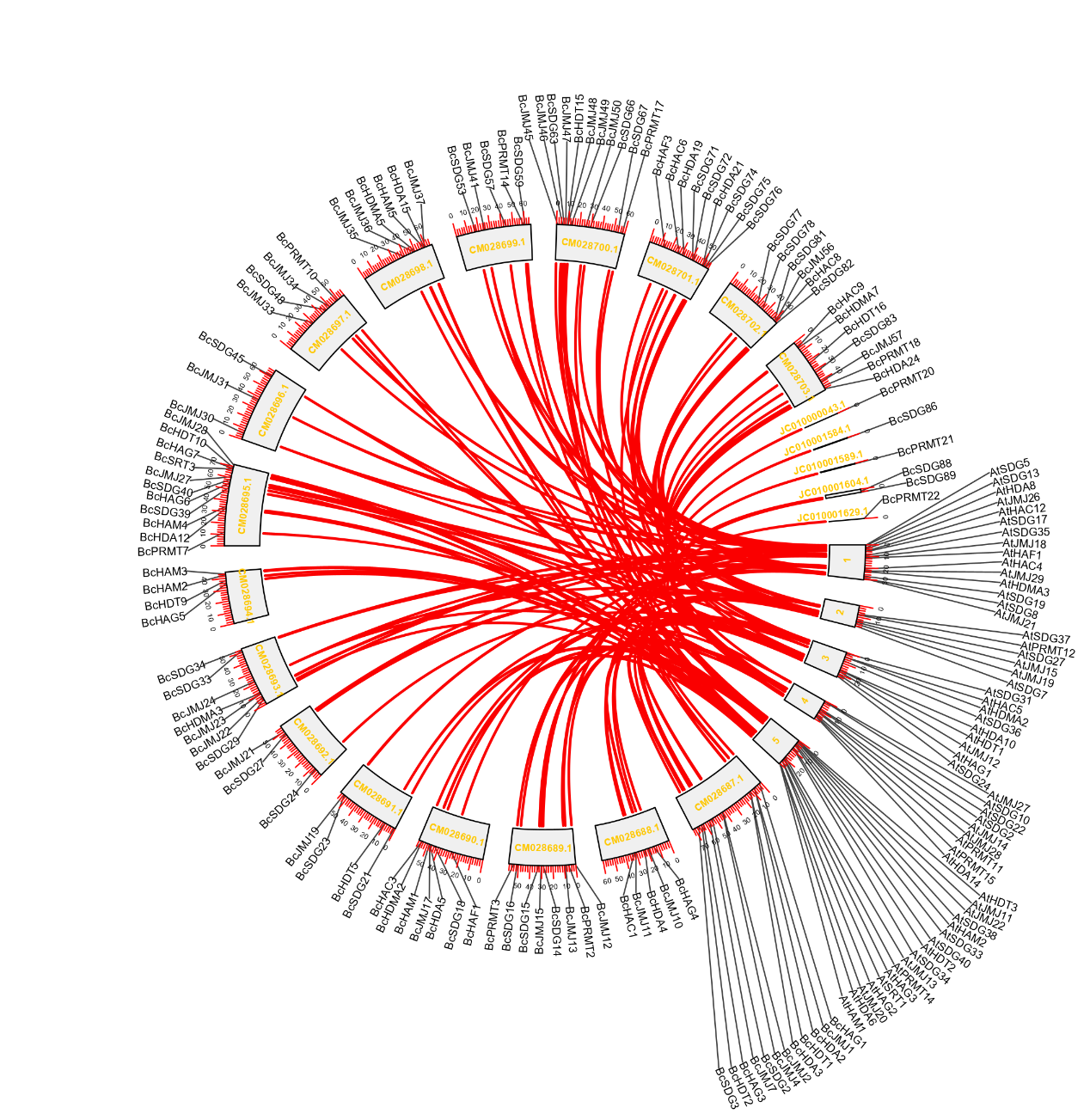


**Fig. S6-2 Synteny of *HM* genes between *Arabidopsis* and *Brassica juncea.***


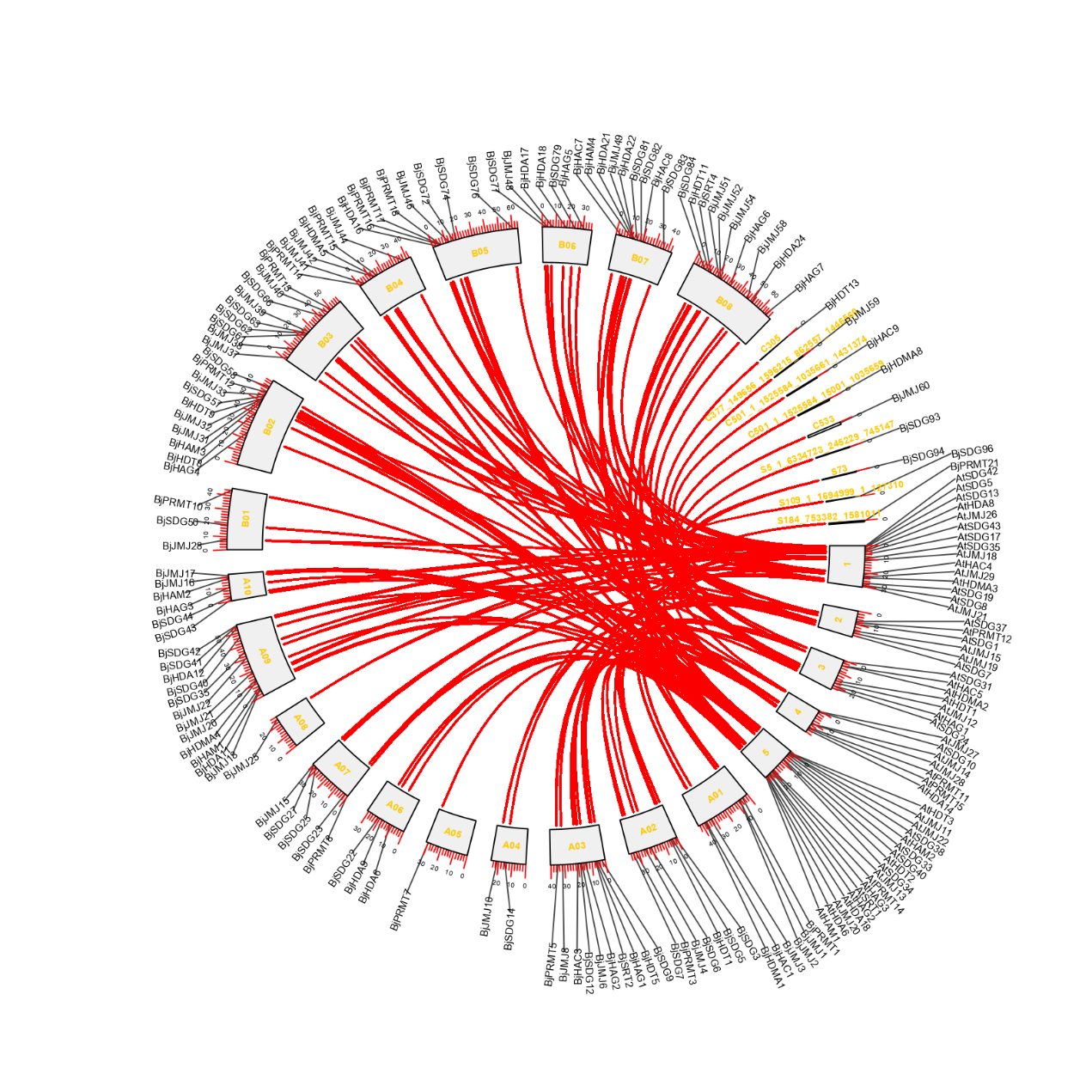


**Fig. S6-3 Synteny of *HM* genes between *Arabidopsis* and *Brassica napus.***


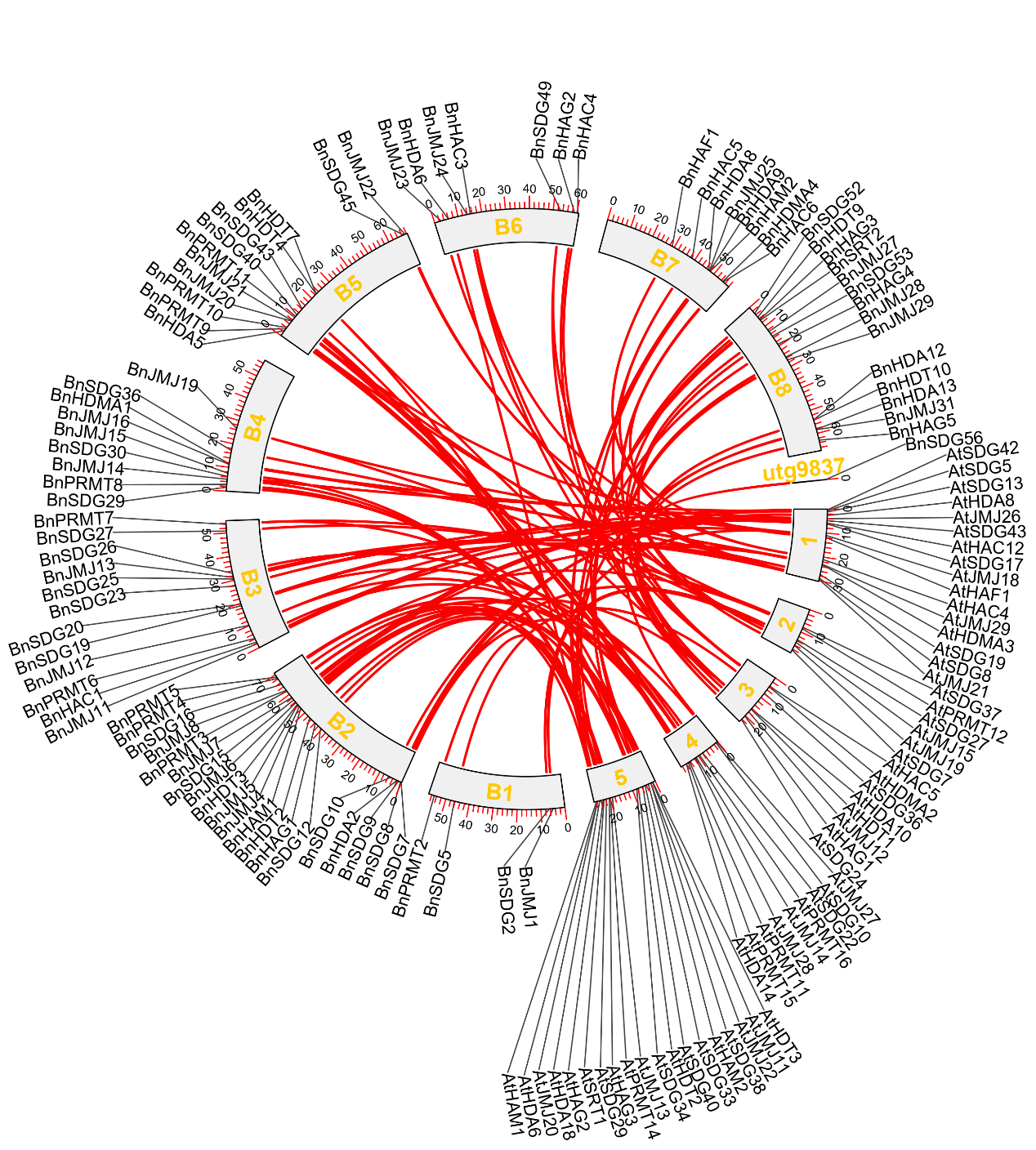


**Fig. S6-4 Synteny of *HM* genes between *Arabidopsis* and *Brassica oleracea.***


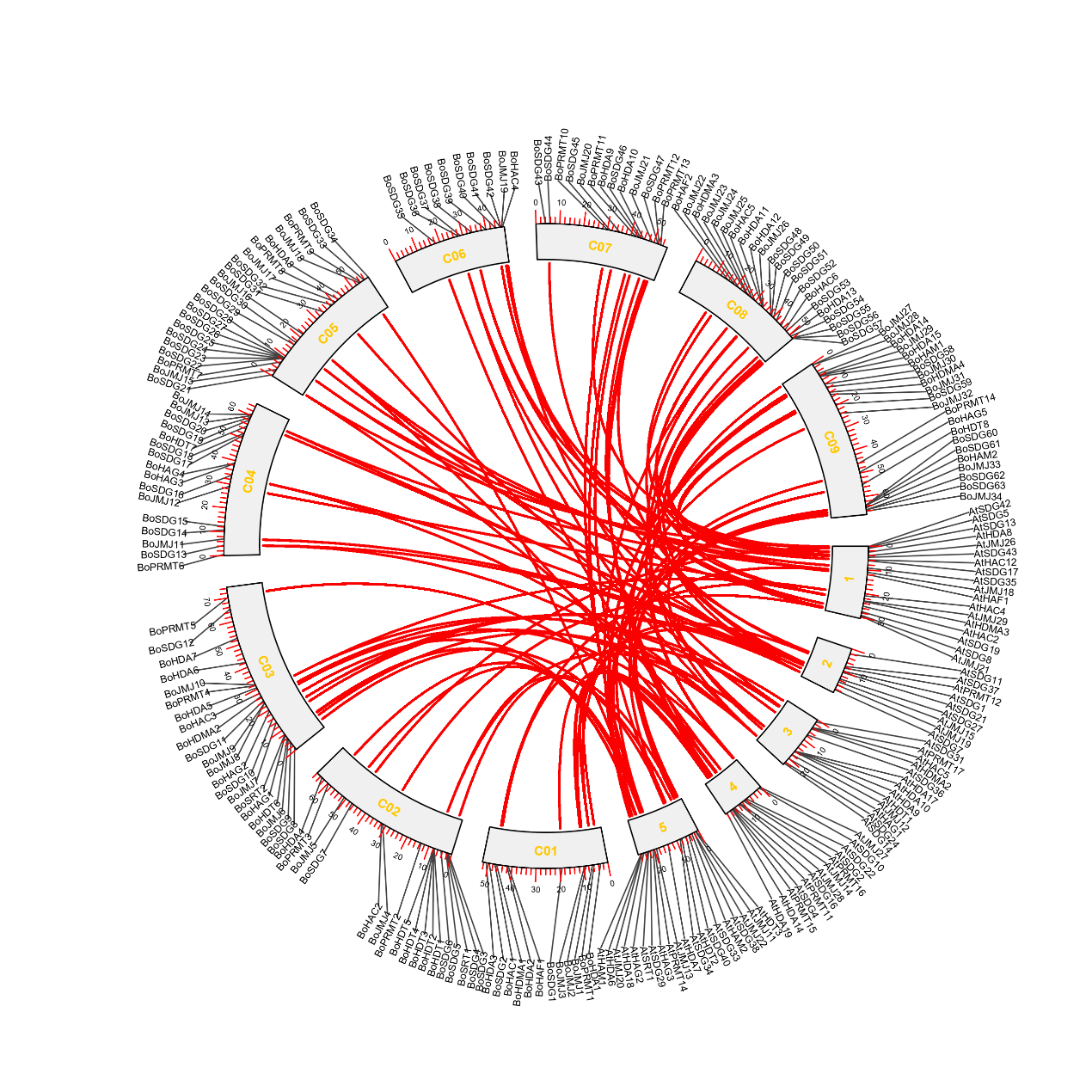


**Fig. S6-5 Synteny of *HM* genes between *Arabidopsis* and *Brassica rapa.***


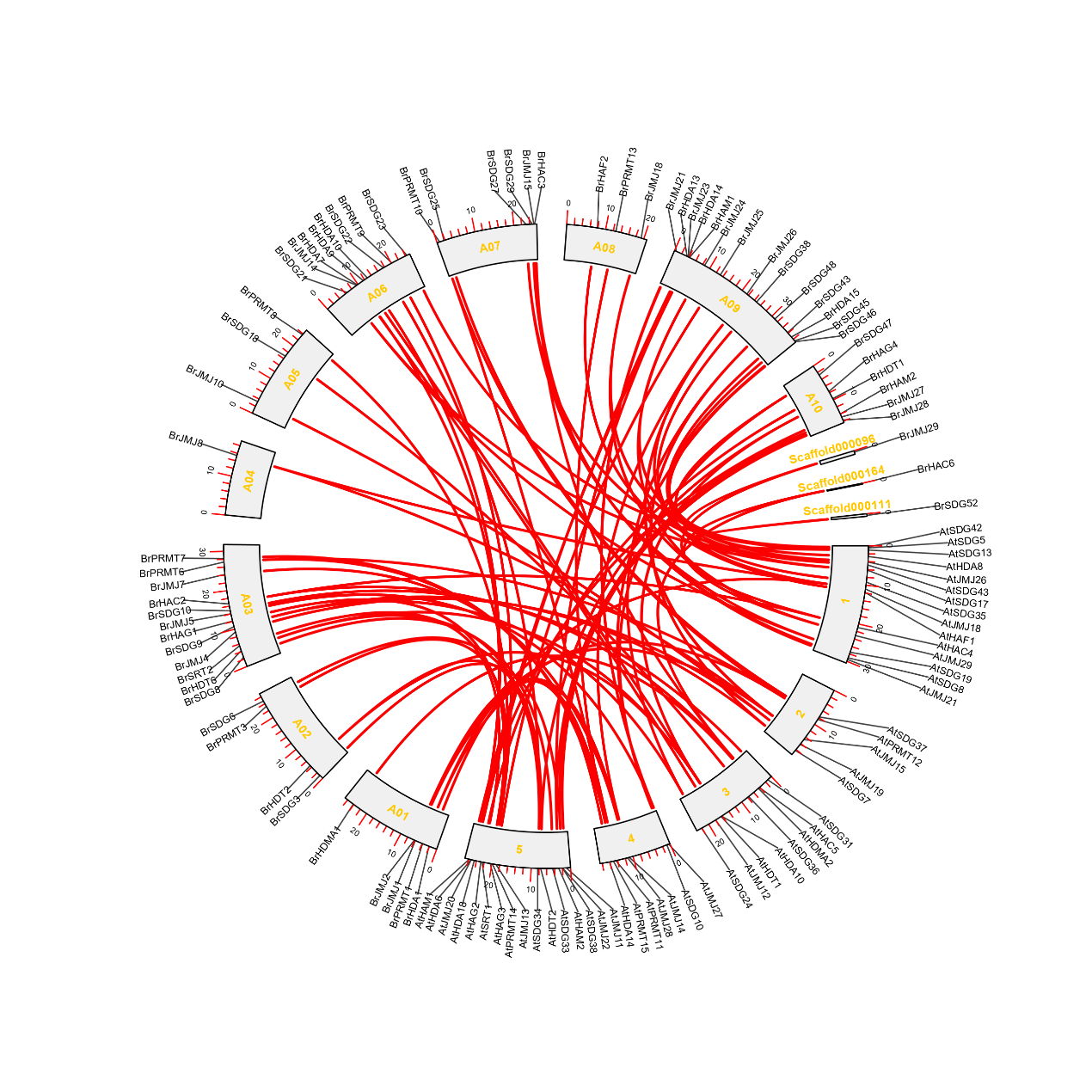


**Fig. S6-6 Synteny of *HM* genes between *Arabidopsis* and *Camelina sativa.***


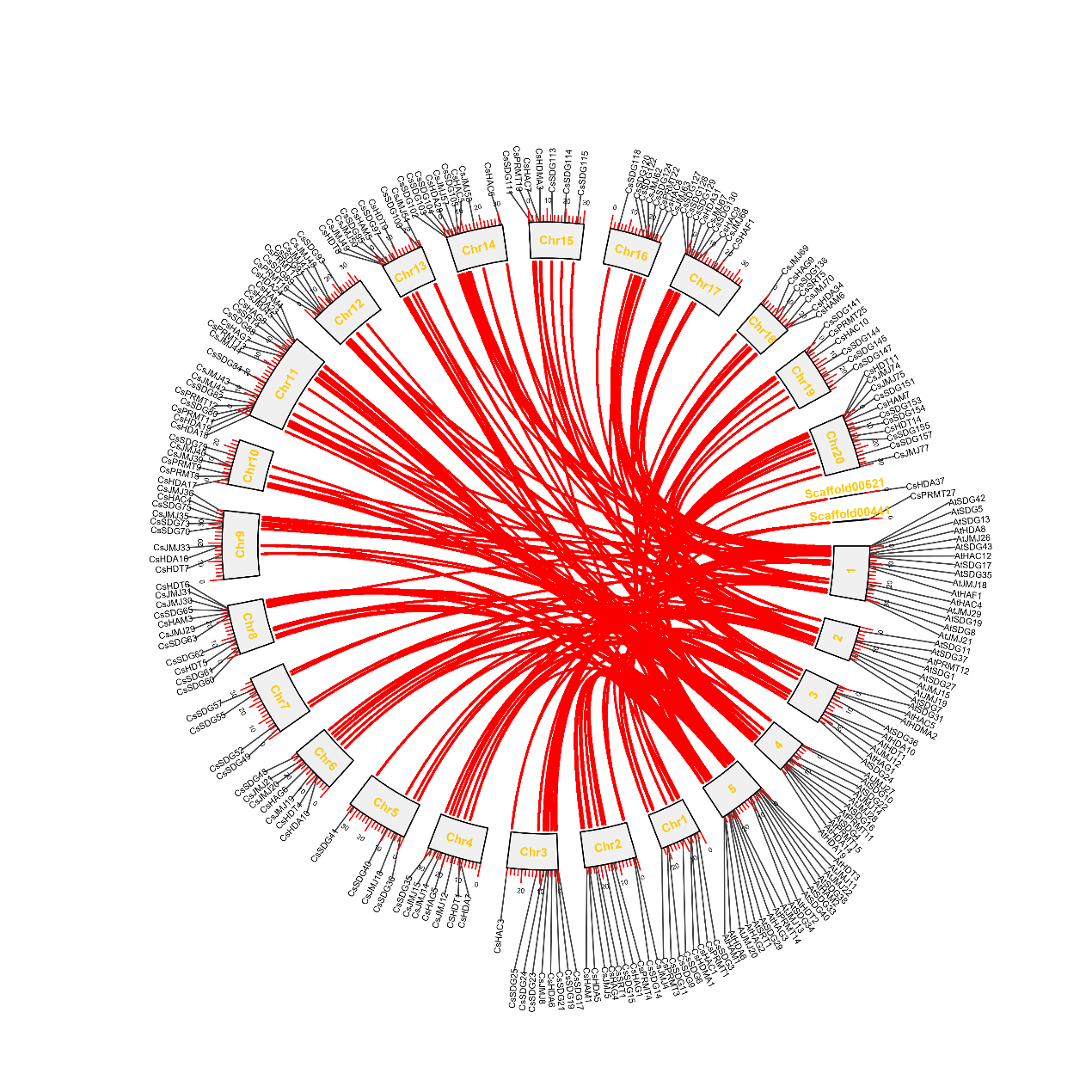


**Fig. S6-7 Synteny of *HM* genes between *Arabidopsis* and *Capsella rubella.***


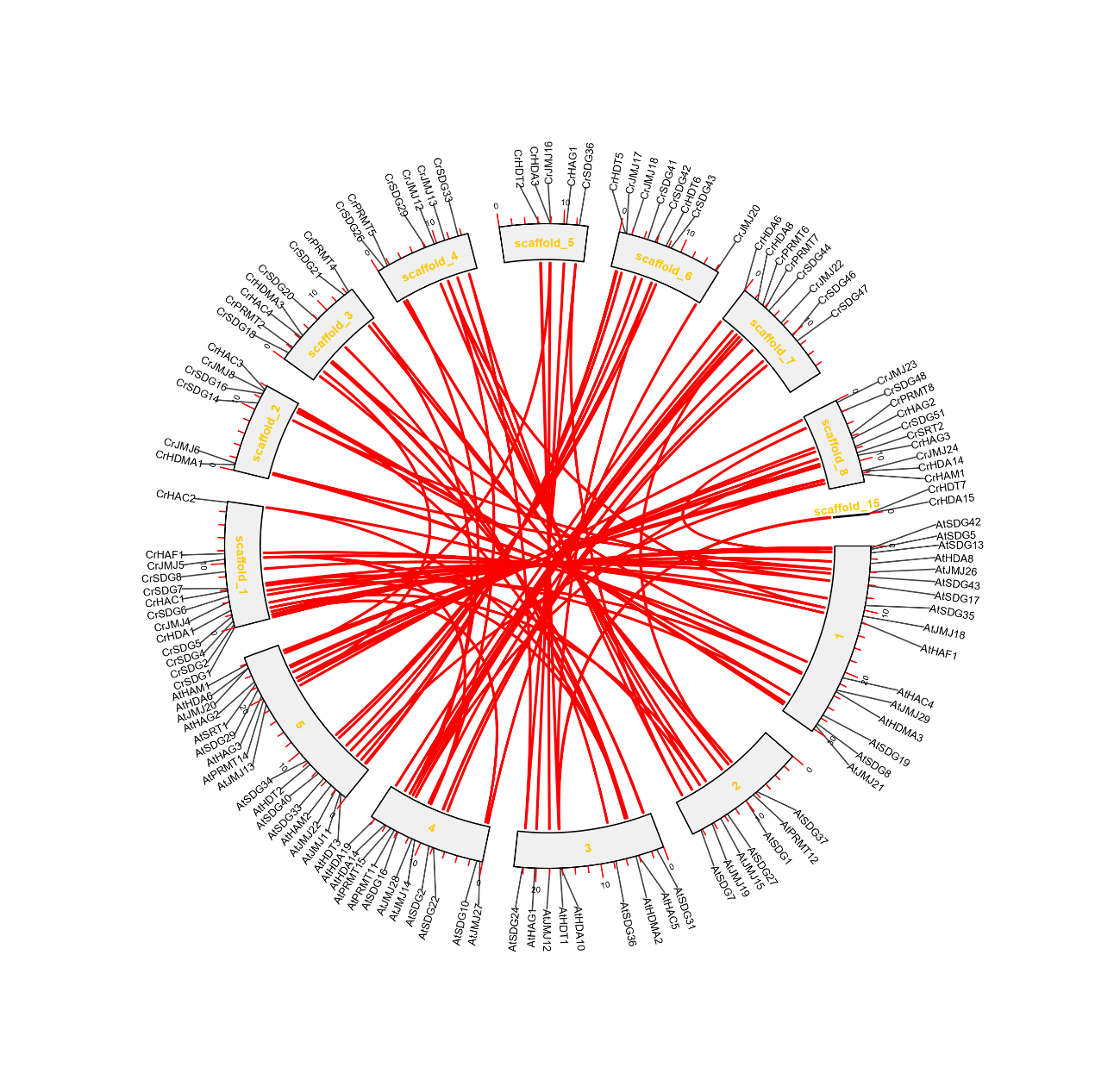


**Fig. S6-8 Synteny of *HM* genes between *Arabidopsis* and *Brassica nigra.***


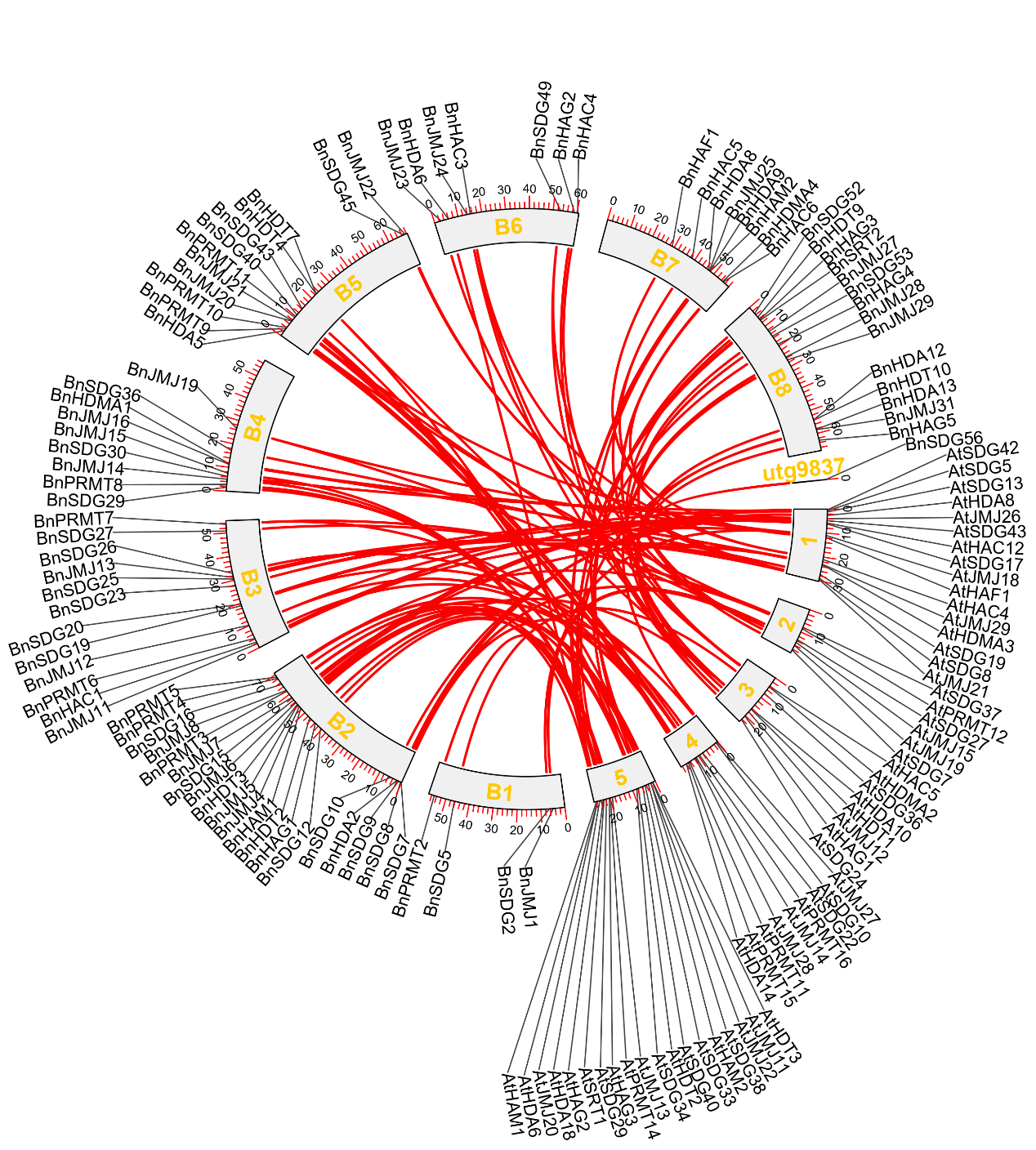

Supplement: Supplementary file 6 — Supplementary Material 6 [file 12870_2023_4256_MOESM6_ESM.docx]
